# Electrocatalytic water oxidation by Cu(ii) complexes: influence of ternarification and chelate ring size

**DOI:** 10.1039/d6ra01803c

**Published:** 2026-07-03

**Authors:** Pranjal Das, Swati Basak, Baisali Hazarika, Apurba Kalita

**Affiliations:** a Department of Chemistry, B. Borooah College Guwahati 781007 Assam India apurbakalitabbc@gmail.com; b Department of Chemistry, Gauhati University Guwahati 781014 Assam India

## Abstract

The electrocatalytic water oxidation activity of two mononuclear ternary copper(ii) complexes, 1 [Cu^II^(L_1_H)(L_2_)](ClO_4_)_2_ [L_1_H = *N*^1^-(2-aminoethyl)ethane-1,2-diamine and L_2_ = pyridin-2-ylmethanamine] and 2 [Cu^II^(L_1_H)(L_3_)](ClO_4_)_2_ [L_3_ = 2-(pyridin-2-yl)ethan-1-amine], has been explored. In a neutral phosphate buffer, complexes 1 and 2 catalyse water oxidation at overpotentials of 423 and 483 mV with turnover frequencies (TOFs) of 157 and 704 s^−1^, respectively. Electrochemical studies reveal that ligands capable of forming six-membered chelate rings enhance the catalytic activity of the copper complexes relative to ligands capable of forming five-membered chelate rings. Furthermore, the incorporation of a redox-active tridentate ligand together with a bidentate ligand capable of forming six-membered chelate rings in ternary copper complexes synergistically enhances the rate of water oxidation catalysis. These findings underscore an effective strategy for improving water oxidation catalysis through the incorporation of cooperative structural elements into synthetic ternary metal complexes.

## Abstract 2

The electrocatalytic water oxidation activity of two mononuclear ternary copper(ii) complexes, 1 and 2, was evaluated. A redox-active tridentate ligand synergistically facilitates electron transfer, enhancing water oxidation catalysis.

## Graphical abstract

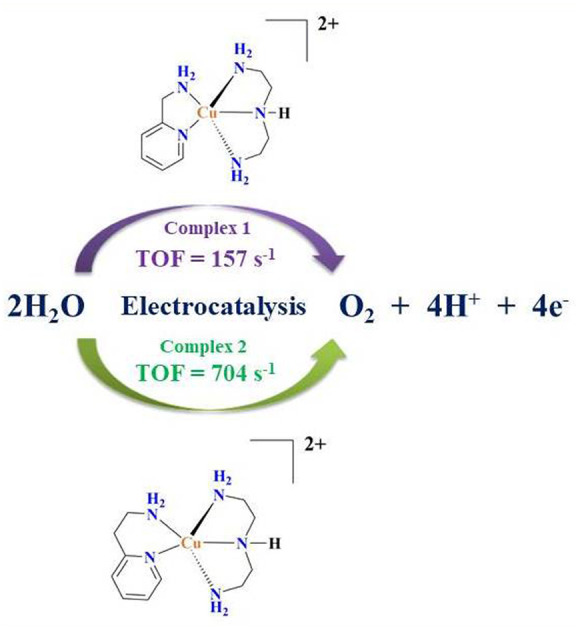

## 1 Introduction

The growing dependence on fossil fuels is becoming increasingly unsustainable because of the rapid depletion of these resources and their detrimental environmental consequences, including greenhouse gas emissions, global warming, and air pollution. Consequently, considerable research efforts have been directed toward the development of renewable and sustainable energy alternatives, among which hydrogen (H_2_) has emerged as a promising clean energy carrier.^1,2^ Among the available hydrogen production methods, water electrolysis is regarded as one of the most environmentally benign approaches.^3–5^ However, the overall efficiency of water electrolysis is largely limited by the sluggish kinetics of the water oxidation reaction (WOR), a complex multi-electron process that requires efficient catalysts to lower the associated energy barrier.

Transition-metal-based catalysts have therefore been extensively investigated for electrocatalytic water oxidation because of their versatile redox behaviour and coordination chemistry.^6–36^ In particular, copper-based homogeneous catalysts have attracted significant attention owing to their earth abundance, tunable redox properties, and structural diversity.^10–36^ The catalytic performance of copper complexes is strongly influenced by the ligand environment surrounding the metal centre. Even subtle modifications in the ligand architecture or coordination geometry can produce substantial changes in the redox behaviour and catalytic efficiency of analogous copper complexes.^20–24,29^ As a result, extensive efforts have been devoted toward understanding the structure–activity relationships of copper complexes toward electrocatalytic water oxidation. Copper complexes containing diverse ligand frameworks, including redox-active,^20,31^ oxidation-resistant,^10^ macrocyclic,^33,34^ peptide-based,^12,26,27^ and rigid aromatic ligands,^35,36^ have been explored under various reaction conditions in the search for efficient and practically viable water oxidation catalysts.

The water oxidation reaction is kinetically demanding because it involves the concerted removal of four electrons and four protons from two water molecules, accompanied by O–O bond formation and dioxygen evolution. In nature, this challenging transformation is efficiently performed by the oxygen-evolving complex (OEC) in Photosystem II.^37^ The exceptional catalytic efficiency of the OEC arises not only from its multinuclear metal core but also from the cooperative involvement of surrounding structural features, such as redox-active tyrosine residues,^38^ coordinated and pendant histidyl moieties,^39^ and other secondary coordination sphere interactions. These observations suggest that the incorporation of cooperative structural elements into synthetic systems may provide an effective strategy for enhancing water oxidation catalysis.

Inspired by such biological systems, the design of synthetic catalysts can benefit from ligand frameworks capable of modulating both the electronic structure and proton-transfer properties around the metal centre. In this context, ternary metal complexes represent attractive platforms because they allow the incorporation of multiple cooperative functionalities within a single coordination framework more effectively than simpler binary systems.^24^ Such a structural flexibility offers opportunities to fine-tune the electronic environment, proton-coupled electron transfer pathways, and overall catalytic efficiency of transition-metal complexes for water oxidation.

Herein, we report the electrocatalytic water oxidation activity of two analogous mononuclear ternary copper(ii) complexes, 1 [Cu^II^(L_1_H)(L_2_)](ClO_4_)_2_ and 2 [Cu^II^(L_1_H)(L_3_)](ClO_4_)_2_, where L_1_H = *N*^1^-(2-aminoethyl)ethane-1,2-diamine, L_2_ = pyridin-2-ylmethanamine, and L_3_ = 2-(pyridin-2-yl)ethan-1-amine. The two complexes differ primarily in the chelate ring size formed around the copper(ii) centre, enabling an evaluation of the influence of subtle structural variations on catalytic activity. Previously, our group reported the electrocatalytic properties of binary copper(ii) complexes containing the bidentate arylalkyl amine ligands L_2_ and L_3_.^29^ In comparison to these binary analogues, the newly synthesized ternary complexes exhibit significantly enhanced electrocatalytic activity upon the incorporation of a redox-active dipodal alkyl amine ligand (L_1_H) into the coordination sphere. The present study, therefore, highlights the important role of cooperative ligand design and subtle coordination environment modulation in tuning the efficiency of copper-based electrocatalysts for water oxidation.

## 2 Results and discussion

### 2.1 Synthesis and characterization

**Fig. 1:**
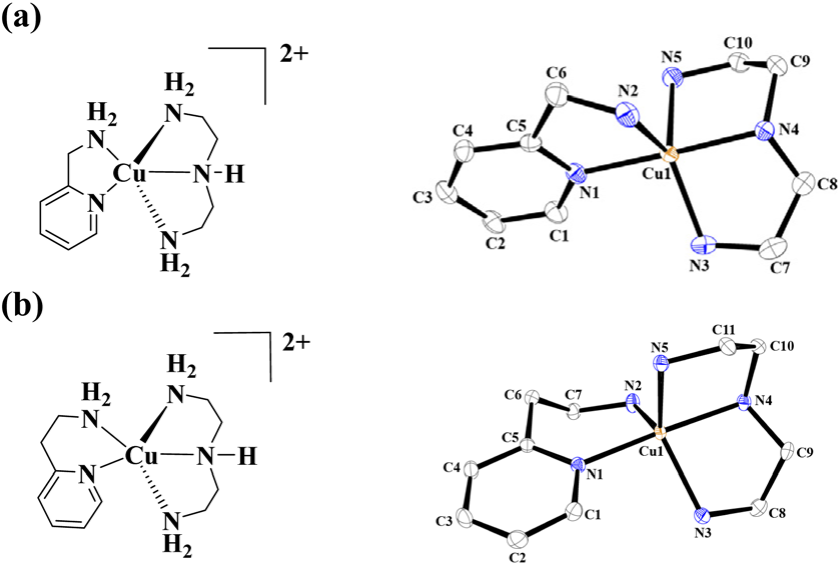
Structures and ORTEP diagrams (50% thermal ellipsoid plot) of (a) complex 1 and (b) complex 2. Hydrogen atoms and perchlorate ions are omitted in the ORTEP diagrams for clarity.

Ternary copper(ii) complexes 1 and 2 were synthesized as perchlorate salts using the tridentate ligand L_1_H (*N*^1^-(2-aminoethyl)ethane-1,2-diamine) together with the bidentate ligands L_2_ (pyridin-2-ylmethanamine) and L_3_ [2-(pyridin-2-yl)ethan-1-amine], respectively (see the Experimental section for details). Single crystals suitable for X-ray diffraction were obtained for both complexes. The single-crystal X-ray structures (Fig. 1) reveal that in both complexes, the copper centre is coordinated by five nitrogen donor atoms. The crystallographic parameters are summarized in Tables S1 and S2. In addition, the powder XRD patterns of the bulk samples closely match the simulated patterns (Fig. S4), confirming the formation of the ternary complexes.

In complex 1, ligand L_2_ forms a five-membered chelate ring with a bite angle of 80.79° around the central Cu(ii) ion, whereas ligand L_3_ forms a six-membered ring with a bite angle of 93.98° around the Cu(ii) ion in complex 2. In a neutral phosphate buffer, UV-Visible spectra show d–d transition bands at 621 nm (*ε* = 126 M^−1^ cm^−1^) and 636 nm (*ε* = 141 M^−1^ cm^−1^) for complexes 1 and 2, respectively (Fig. S5). In both water and neutral phosphate buffer media, the UV-Visible spectra of the complexes are identical (Fig. S6 and S7). Further, to explore the stability of the complexes in a neutral phosphate buffer medium, UV-Visible spectra were recorded at different time intervals, which remain unchanged for both the complexes (Fig. S8 and S9).

### 2.2 Redox properties

The redox properties of the complexes were investigated in a 0.1 M neutral phosphate buffer medium using cyclic voltammograms (CVs) and differential pulse voltammograms (DPVs). For recording CVs and DPVs, a single-compartment electrochemical cell fitted with a glassy carbon (GC) working electrode with an area of 0.07 cm^2^, an Ag/AgCl reference electrode and a platinum wire counter electrode was used. The peak potentials of CVs and DPVs are reported with respect to the NHE (normal hydrogen electrode), which was obtained by adding 0.197 V to the recorded potential (*vs.* Ag/AgCl).

**Fig. 2:**
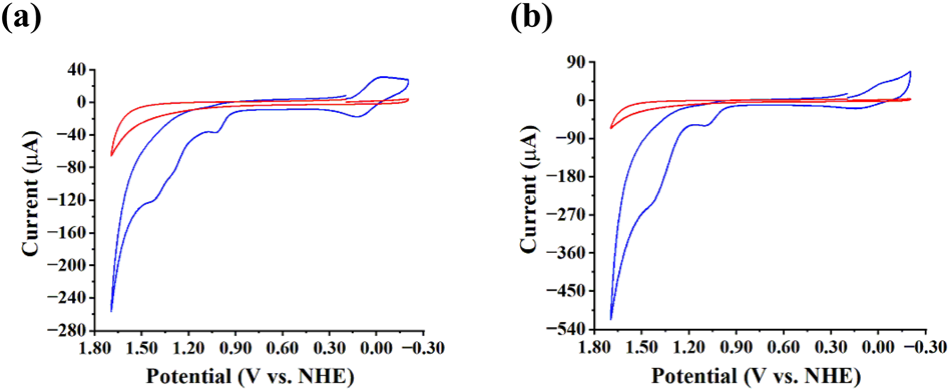
(a) CVs recorded at a scan rate of 100 mV s^−1^ with a 1 mM solution of complex 1 (blue line) and without complex 1 (red line) in a 0.1 M neutral phosphate buffer medium. (b) CVs recorded at a scan rate of 100 mV s^−1^ with a 1 mM solution of complex 2 (blue line) and without complex 2 (red line) in a 0.1 M neutral phosphate buffer medium.

The CVs of the complexes are shown in Fig. 2, and from the half-peak potential of the observed catalytic peak, the onset potentials of the complexes were calculated.^40^ The values of onset potentials at which water oxidation occurs are found to be 1.24 V and 1.30 V *vs.* NHE, corresponding to overpotentials of 423 mV and 483 mV for complexes 1 and 2, respectively (Fig. S10).

**Fig. 3:**
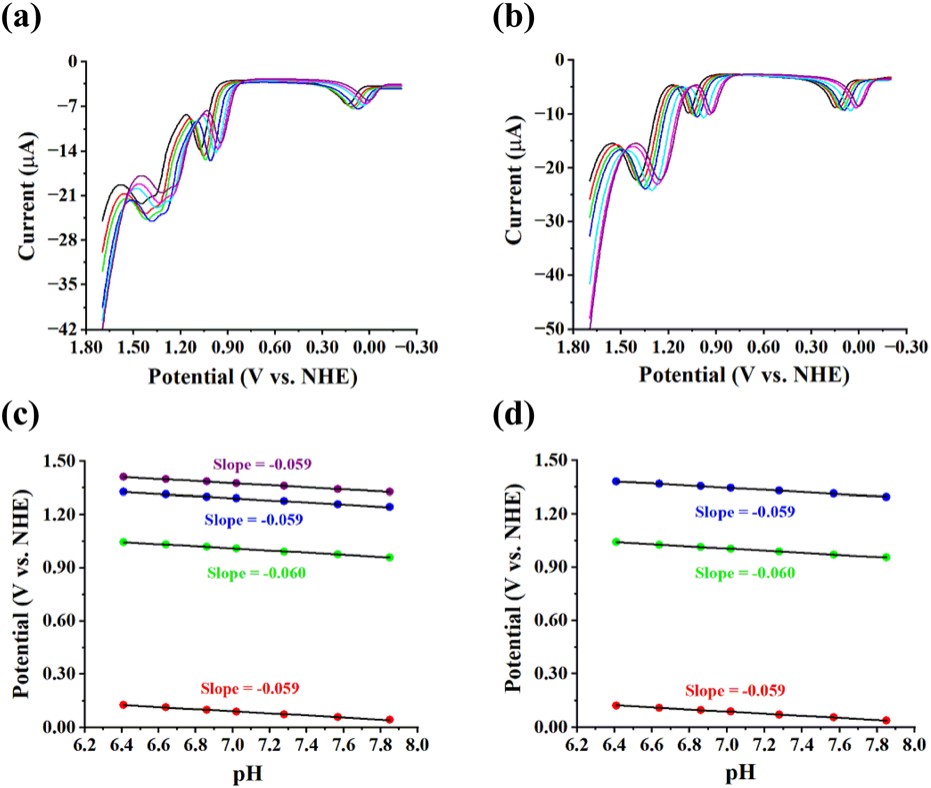
(a) DPV plots of a 0.5 mM solution of complex 1 in a 0.1 M phosphate buffer of pH 6.41 (black), 6.64 (red), 6.86 (green), 7.02 (blue), 7.28 (cyan), 7.57 (magenta) and 7.85 (purple). (b) DPV plots of a 0.5 mM solution of complex 2 in a 0.1 M phosphate buffer of pH 6.41 (black), 6.64 (red), 6.86 (green), 7.02 (blue), 7.28 (cyan), 7.57 (magenta) and 7.85 (purple). (c) Pourbaix diagram of complex 1 depicting the variation of the peak potential of the first (red dots), second (green dots), third (blue dots) and fourth (purple dots) anodic peaks with respect to the pH of the 0.1 M phosphate buffer. (d) Pourbaix diagram of complex 2 depicting the variation of the peak potential of the first (red dots), second (green dots) and third (blue dots) anodic peaks with respect to the pH of the 0.1 M phosphate buffer.

For complexes 1 and 2, DPVs show four and three anodic peaks, respectively [Fig. 3(a) and (b)]. The first anodic peak at 0.077 V and 0.097 V *vs.* NHE for complexes 1 and 2, respectively, is assigned to the Cu^II^/Cu^I^ couple and shows a pH-dependency of 0.059 V per pH unit.

The second anodic peak appears at around +1.009 V and +1.021 V *vs.* NHE for complexes 1 and 2, respectively. Analysis of the Pourbaix diagrams [Fig. 3(c) and (d)] and calculation using Laviron equations^41^ (Fig. S11 and S12) indicate the involvement of two electrons and two protons for complex 1 and one electron and one proton for complex 2 in this step. A comparison of the CVs and DPVs obtained for analogous Zn complexes (Fig. S13 and S14) and individual ligand moieties (Fig. S15) suggests the oxidation of the alkyl amine ligand (L_1_H) at this potential.^24^

The third anodic peak occurs at +1.264 V and +1.349 V *vs.* NHE for complexes 1 and 2, respectively. Analysis of the Pourbaix diagrams [Fig. 3(c) and (d)] and calculation using Laviron equations^41^ (Fig. S11 and S12) indicate the involvement of one electron and one proton for complex 1 and three electrons and three protons for complex 2 at this potential. Complex 1 further oxidizes at the potential of the fourth anodic peak (+1.386 V *vs.* NHE) and shows the involvement of one electron and one proton.

Computational calculations using density functional theory (DFT) were carried out using the Gaussian 09 package to get an insight into the catalytic cycle of water oxidation by the complexes.^42^ The B3LYP functional was used to optimise the most stable forms of all reactive intermediates (Fig. S16–S25). For the Cu atom, the LanL2DZ basis set was used, whereas for C, H, N and O atoms, the 6-31G** basis set was used to carry out the optimisation process. The SMD continuum solvation model was used to incorporate the solvation effects in the aqueous medium for all the intermediates.

In an aqueous medium at pH 7, the redox potential of each individual step was calculated using established protocols.^28^ At pH 7, the DFT-calculated redox potential for water oxidation is found to be 0.720 V *vs.* NHE, which gives a deviation of 0.097 V *vs.* NHE (2.237 kcal mol^−1^) from the experimentally obtained potential value (0.817 V *vs.* NHE). Accordingly, a correction factor of +0.097 V was applied to all DFT-computed redox potentials to obtain more reliable driving forces. This correction strategy has been widely employed in previous studies on water-oxidation catalysts.^28^

**Scheme 1:**
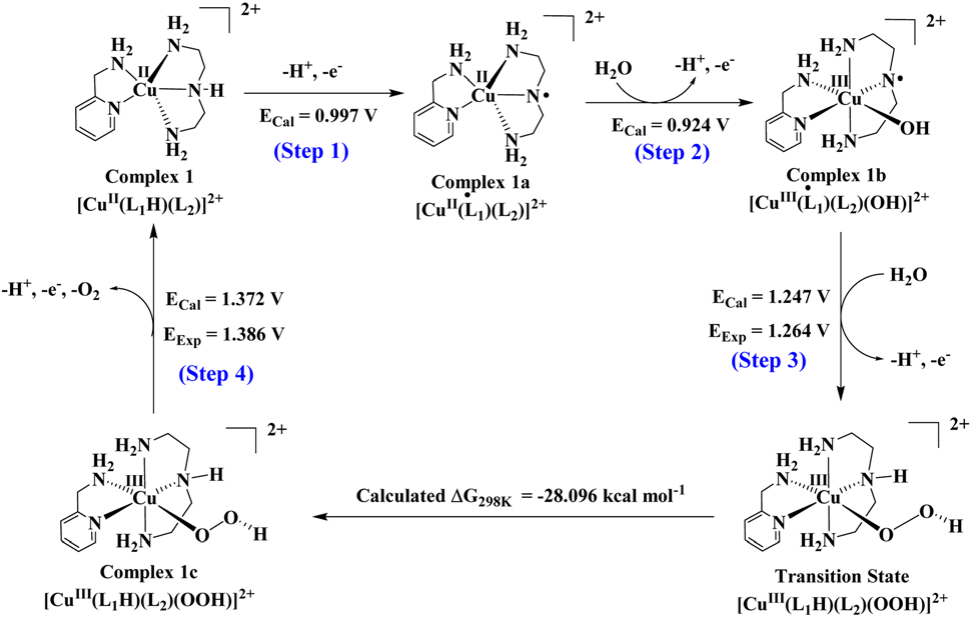
Proposed catalytic cycle of water oxidation catalysed by complex 1. *E*_Cal_ and *E*_Exp_ refer to the theoretically calculated and experimental redox potentials, respectively, *versus* the reference NHE at pH 7. The experimental redox potential for the formation of complex 1b from complex 1 is found to be 1.009 V *vs.* NHE.

Complex 1, [Cu^II^(L_1_H)(L_2_)]^2+^, is oxidized to complex 1a, [Cu^II^(L_1_˙)(L_2_)]^2+^, *via* a ligand-based proton-coupled electron transfer (PCET) step with a calculated redox potential of 0.997 V *vs.* NHE. Complex 1a then undergoes a reaction with a solvent water molecule and a subsequent PCET step to afford complex 1b, [Cu^III^(L_1_˙)(L_2_)(OH]^2+^. The redox potential for this step is calculated to be 0.924 V *vs.* NHE. Since the potential of this second step is lower than that of the first step, an experimental two electron-two proton direct oxidation step from complex 1 to complex 1b is possible (Fig. S28). The high oxidizing power that accumulates at complex 1b drives the reaction with another solvent water molecule, and it undergoes a one electron-one proton process to initiate O–O bond formation through a transition state (*E*_cal_ = 1.247 V *vs.* NHE) to afford complex 1c, [Cu^III^(L_1_H)(L_2_)(OOH)]^2+^ (Δ*G*_298 K_ = −28.096 kcal mol^−1^). Complex 1c then undergoes another PCET step to release molecular oxygen and regenerate complex 1. The redox potential calculated for the regeneration of complex 1 from complex 1c is 1.372 V *vs.* NHE. Based on these experimental and computational studies, a catalytic cycle for water oxidation catalysed by complex 1 is proposed, as shown in Scheme 1.

**Scheme 2:**
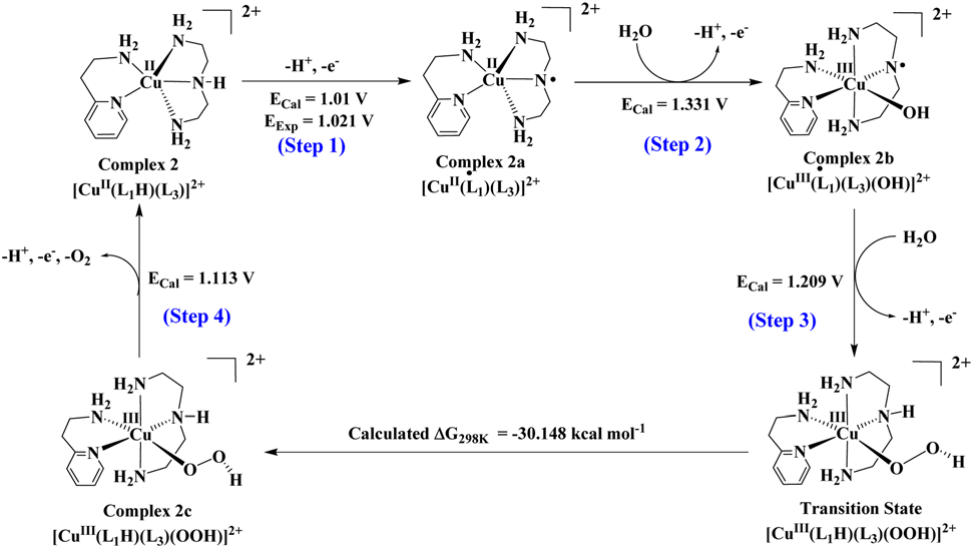
Proposed catalytic cycle of water oxidation catalysed by complex 2. *E*_Cal_ and *E*_Exp_ refer to the theoretically calculated and experimental redox potentials, respectively, *versus* the reference NHE at pH 7. The experimental redox potential for the release of molecular oxygen and regeneration of complex 2 from complex 2a is found to be 1.349 V *vs.* NHE.

DFT calculations suggest that complex 2, [Cu^II^(L_1_H)(L_3_)]^2+^, is oxidized to complex 2a, [Cu^II^(L_1_˙)(L_3_)]^2+^, *via* a ligand-based proton-coupled electron transfer (PCET) step. The redox potential for this step is calculated to be 1.01 V *vs.* NHE, which closely matches the experimental data (Fig. S29). Complex 2a further reacts with a solvent water molecule and undergoes a one electron-one proton process to afford complex 2b, [Cu^III^(L_1_˙)(L_3_)(OH)]^2+^. The redox potential calculated for this step is 1.331 V *vs.* NHE. The high oxidizing power that accumulates at complex 2b drives the reaction with another solvent water molecule, and it undergoes a one electron-one proton process to initiate O–O bond formation through a transition state (*E*_cal_ = 1.209 V *vs.* NHE) to afford complex 2c, [Cu^III^(L_1_H)(L_3_)(OOH)]^2+^ (Δ*G*_298 K_ = −30.148 kcal mol^−1^). Complex 2c then undergoes another PCET step to release molecular oxygen and regenerate complex 2. The redox potential calculated for the regeneration of complex 2 from complex 2c is 1.113 V *vs.* NHE. Here, it is interesting to note that the calculated redox potential for the fourth and third steps is lower than that for the second step, which suggests that the three electron-three proton direct oxidation of complex 2a to release molecular oxygen and regenerate the catalyst is possible. Based on these experimental and computational studies, a catalytic cycle for water oxidation catalysed by complex 2 is proposed, as shown in Scheme 2.

The overall energy profiles for water oxidation catalyzed by complexes 1 and 2 are shown in Fig. S26 and S27. The assignments of theoretically calculated redox potentials with experimentally observed anodic peak potentials for complexes 1 and 2 are given in Fig. S28 and S29. The Gibbs free energies of all intermediates in an aqueous solution are summarized in Tables S3 and S4 for complexes 1 and 2, respectively.

The formation of Cu(iii)-hydroperoxy intermediates and complexes 1c and 2c was also confirmed by the successive addition of horseradish peroxidase (HPR) and Ampliflu red (AR) reagents to 0.5 mM solutions of complexes 1 and 2 after 4 hours of controlled potential electrolysis (CPE). The blue colour of the solutions of complexes 1 and 2 turns pink upon the addition of HRP and AR during the CPE experiment (Fig. S30 and S31).^13,22,23^

### 2.3 Kinetics of electrocatalysis

To investigate the kinetics of the electrocatalytic process, CVs were recorded at different concentrations of complexes 1 and 2 at a scan rate of 100 mV s^−1^, which indicates that the catalytic current increases linearly with the concentration of both the complexes (Fig. S32 and S33). This suggests first-order kinetics as well as a single-site mechanism for water oxidation catalysis by complexes 1 and 2 and can be described by eqn (1) as follows:^43^

1 *i*_cat_ = *n*_cat_*FA*[*C*](*k*_cat_*D*_Cu_)^1/2^,

where *i*_cat_ is the catalytic peak current, *n*_cat_ (=4) is the total number of electrons involved in water oxidation, *F* is the Faraday constant, *A* is the area of the working electrode (0.07 cm^2^), [*C*] is the concentration of the catalyst, *k*_cat_ is the catalytic rate constant for water oxidation and *D*_Cu_ is the diffusion coefficient.

To determine the diffusion coefficient (*D*_Cu_) values, CVs were recorded at different scan rates for complexes 1 and 2. The anodic peak currents (*i*_d_) of the Cu^II^/Cu^I^ couple vary linearly with the scan rate for both the complexes. Using the Randles–Sevcik equation^28,43^ (eqn (2)) and from the plot of the anodic peak current (*i*_d_) of the Cu^II^/Cu^I^ couple *vs.* the square root of the scan rate (*ν*^1/2^), the values of *D*_Cu_ were calculated. For complexes 1 and 2, they are calculated to be 13.724 × 10^−6^ cm^2^ s^−1^ and 16.260 × 10^−6^ cm^2^ s^−1^, respectively (Fig. S34 and S35).

2 *i*_d_ = 0.446*α*^1/2^*nFA*[*C*]{(*nFνD*_Cu_)/RT}^1/2^,

where *i*_d_ is the anodic peak current of the Cu^II^/Cu^I^ couple, *α* (=0.5) is the transfer coefficient for the irreversible Cu^II^/Cu^I^ couple, *n* is the number of electrons involved with the Cu^II^/Cu^I^ couple (for complexes 1 and 2, *n* = 1), *ν* is the scan rate, *R* is the ideal gas constant and *T* is the temperature.

**Fig. 4:**
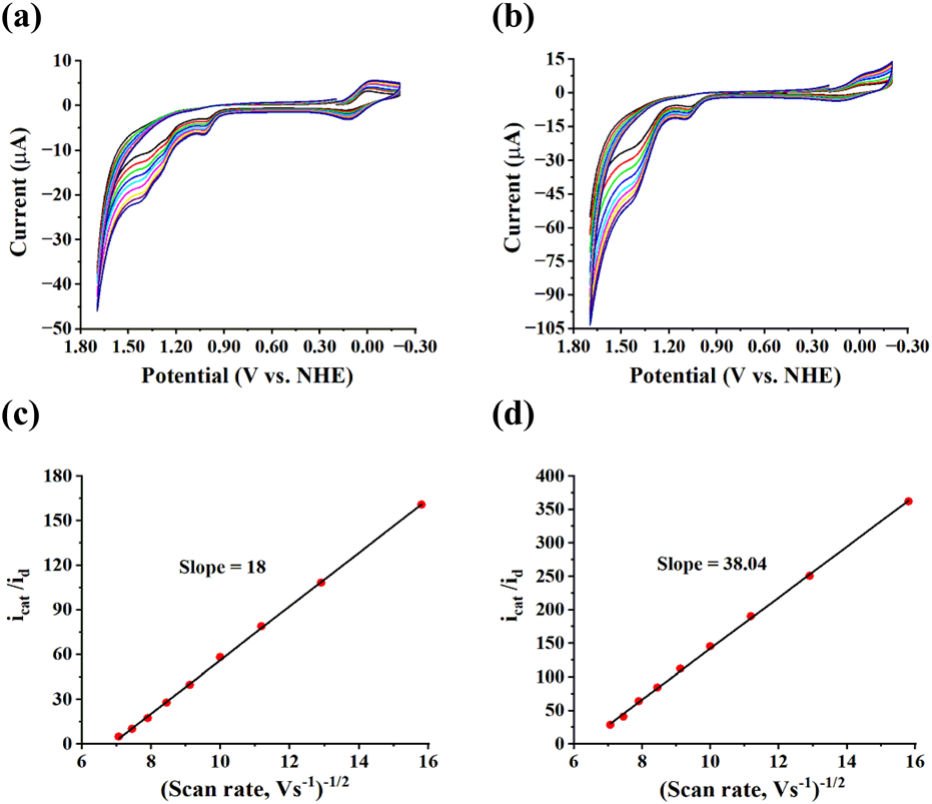
CV plots recorded at 4 (black), 6 (red), 8 (green), 10 (blue), 12 (cyan), 14 (magenta), 16 (yellow), 18 (purple) and 20 (royal) mV s^−1^ scan rates in a 0.1 M neutral phosphate buffer for (a) complex 1 and (b) complex 2. Plot of *i*_cat_/*i*_d_*vs. ν*^−1/2^ for (c) complex 1 and (d) complex 2.

The values of the catalytic rate constant (*k*_cat_) were obtained using eqn (3) and by recording CVs at different scan rates (4–20 mV s^−1^) [Fig. 4(a) and (b)] for both the complexes. Eqn (3) was obtained by combining eqn (1) and (2) as follows:

3 *i*_cat_/*i*_d_ = 2.242 (*n*_cat_/*n*)(RT/*nF*)^1/2^ (*k*_cat_)^1/2^*ν*^−1/2^,

Now, the values of *i*_cat_/*i*_d_ were plotted against the values of *ν*^−1/2^ [Fig. 4(c) and (d)], and from the slope of this linear plot, the values of *k*_cat_ were determined. For complexes 1 and 2, these values are found to be 157 s^−1^ and 704 s^−1^, respectively.

**Fig. 5:**
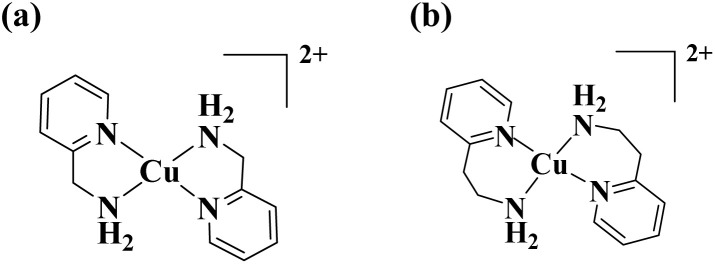
Structures of the binary complexes of (a) ligand L_2_ and (b) ligand L_3_.

The calculated values of the catalytic rate constant of these ternary complexes show significant improvement compared with binary complexes (Fig. 5) with ligands L_2_ and L_3_.^29^ This enhanced catalytic performance of complexes 1 and 2 relative to their binary analogues underscores the critical role of the tridentate flexible ligand L_1_H in catalytic efficiency. A comparison of the catalytic performance of complexes 1 and 2 with other reported pyridine-based and aliphatic amine ligand-containing copper complexes is given in Table S25.

### 2.4 Controlled potential electrolysis

**Fig. 6:**
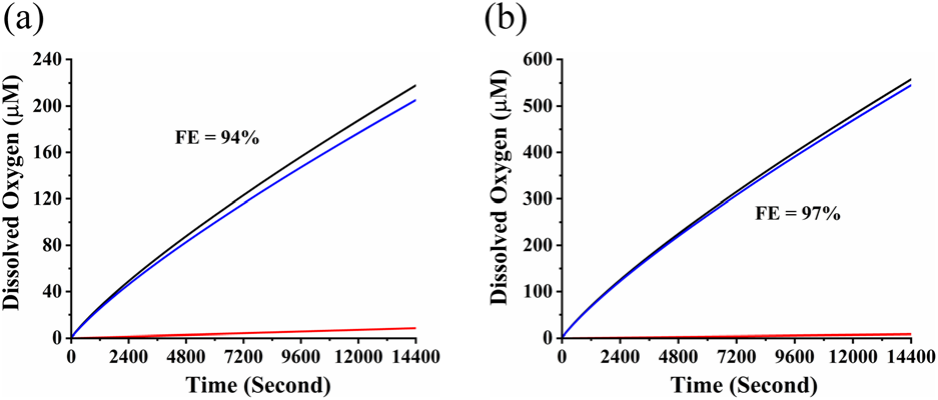
(a) Determination of the FE during 4 hours of the CPE experiment with complex 1 (blue line) and without the complex (red line). The black line indicates 100% FE, which is obtained from the amount of charge produced during the CPE experiment. (b) Determination of FE during 4 hours of the CPE experiment with complex 2 (blue line) and without the complex (red line). The black line indicates 100% FE, which is obtained from the amount of charge produced during the CPE experiment.

To investigate the oxygen evolution process, a controlled potential electrolysis (CPE) experiment was carried out in a gas-tight two-compartment cell using an ITO working electrode (surface area = 4 cm^2^). Oxygen evolved in the solution during the CPE experiment was measured using a calibrated Ocean Optics FOXY probe. For complexes 1 and 2, the CPE experiment was carried out at 1.38 V and 1.35 V *vs.* NHE, respectively (Fig. S36). After 4 hours of the CPE experiment, the Faradaic efficiency (FE) was estimated to be 94% and 97% (Fig. 6) for complexes 1 and 2, respectively. For complexes 1 and 2, the turnover number (TON) was calculated to be 0.41 and 1.09, respectively, during 4 hours of the CPE experiment.

To monitor the electrochemical stability of the complexes, the cyclic voltammograms and UV-Visible spectra of the complexes were recorded before and after the CPE experiment, which were almost identical (Fig. S37 and S38). CVs recorded before and after the CPE experiment with the ITO working electrode in a neutral phosphate buffer without complexes were essentially identical (Fig. S39). FE-SEM images and EDX data confirmed that no electroactive species were deposited on the ITO working electrode during 4 hours of the CPE experiment (Fig. S40). Moreover, no significant changes were observed in the peak currents and potentials of complexes 1 and 2 during consecutive CV scans (Fig. S41). The dynamic light scattering (DLS) size distribution analysis conducted on complexes 1 and 2 following consecutive cyclic voltammetry (CV) scans demonstrated that no new catalytically active nanoparticles were generated (Fig. S42 and S43).

**Fig. 7:**
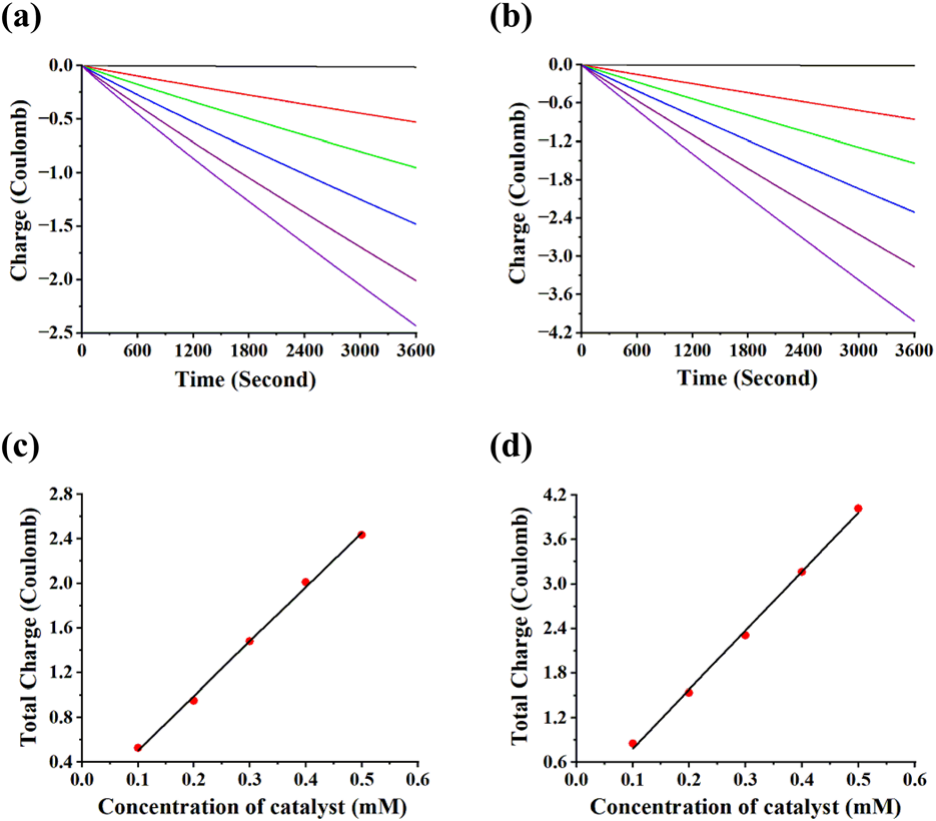
(a) Plot of the charge *vs.* time during 1 hour of the CPE experiment at 1.38 V *vs.* NHE for complex 1 at 0.0 mM (black), 0.1 mM (red), 0.2 mM (green), 0.3 mM (blue), 0.4 mM (navy) and 0.5 mM (purple) concentrations. (b) Plot of the charge *vs.* time during 1 hour of the CPE experiment at 1.35 V *vs.* NHE for complex 2 at 0.0 mM (black), 0.1 mM (red), 0.2 mM (green), 0.3 mM (blue), 0.4 mM (navy) and 0.5 mM (purple) concentrations. (c) Plot of the concentration of complex 1*vs.* total charge after 1 hour of the CPE experiment. (d) Plot of the concentration of complex 2*vs.* total charge after 1 hour of the CPE experiment.

The total charge passed after 1 hour of electrolysis under identical conditions showed a linear dependence on the initial concentration of complexes 1 and 2 (Fig. 7), without any observable induction period during the early stage of electrolysis. This behaviour provides strong evidence for homogeneous single-site water oxidation catalysis. Furthermore, the absence of any newly formed electroactive species, either adsorbed on the working electrode or in the electrolyte solution, collectively supports the homogeneous nature of the catalytic process.

## 3 Conclusions

In conclusion, ternary copper(ii) complexes 1 and 2 have been established as stable homogeneous electrocatalysts for water oxidation under neutral phosphate buffer conditions. Both complexes exhibit enhanced catalytic performance relative to their binary analogues, thereby underscoring the critical role of the ligand environment in dictating catalytic efficiency. Comprehensive electrochemical and computational investigations further demonstrate that complex 2, incorporating a six-membered chelate ring within its ligand framework, exhibits superior catalytic activity to complex 1, which is constrained by a five-membered chelate ring. This distinction highlights the significant influence of the chelate ring size on the overall catalytic proficiency of copper-based water oxidation catalysts.

## 4 Experimental section

### 4.1 Materials and methods

All reagents and solvents were purchased from commercial sources and used without further purification. The UV-Visible spectra and FT-IR spectra (KBr pellets) of the complexes were recorded on a Cary-60 UV-Visible spectrophotometer and a Cary 630 spectrophotometer, respectively. A Cambridge magnetic balance was used for the measurement of the magnetic moments of the complexes. An Eutech instrument CON 700 was used for the measurement of the conductivity of the complexes. A Thermo Scientific Flashmart analyzer was used for elemental analysis of the complexes. Electrochemical measurements were performed using a CHI 7035E bipotentiostat. For FE-SEM studies, a Carl Zeiss Supra 55 electron microscope was used after Au coating. To obtain EDX spectra, a 20-kV electron beam was used. A Litesizer 500 instrument was used for DLS measurements. A Bruker-D8 Advance X-ray diffractometer was used for collecting the powder X-ray diffraction (PXRD) data of the complexes. A Bruker Smart Apex Duo diffractometer with MoKα radiation (*λ* = 0.71073 Å) was used for collecting single-crystal X-ray diffraction (SCXRD) data.

### 4.2 Synthesis of complex 1, [Cu(L_1_H)(L_2_)](ClO_4_)_2_

To a methanolic solution containing 0.28 g (2.7 mmol) of ligand L_1_H [*N*^1^-(2-aminoethyl)ethane-1,2-diamine] and 0.30 g (2.7 mmol) of ligand L_2_ (pyridine-2-ylmethanamine), 15 mL of a methanolic solution containing 1.03 g (2.7 mmol) of copper(ii) perchlorate hexahydrate, [Cu(H_2_O)_6_](ClO_4_)_2_, was added dropwise in a 100-mL round-bottom flask. After stirring the resulting mixture for 1 hour at room temperature, a blue-coloured precipitate of complex 1 was obtained. The precipitate was filtered, and the residue was recrystallized in a methanol–water mixture to obtain blue-coloured crystals. Yield: 0.92 g (∼84%). Elemental analyses: Calcd for C_10_H_21_N_5_Cl_2_O_8_Cu (%): C, 25.35; H, 4.47; N, 14.78. Found (%): C, 25.32; H, 4.48; N, 14.75. FT-IR (KBr pellet): 3428, 3215, 3118, 2911, 1608, 1152, 1118 cm^−1^. Magnetic moment: 1.66 µB. The molar conductance of complex 1 in an acetonitrile solution was found to be 205 S cm^2^ mol^−1^.

### 4.3 Synthesis of complex 2, [Cu(L_1_H)(L_3_)](ClO_4_)_2_

To a methanolic solution containing 0.42 g (4.0 mmol) of ligand L_1_H [*N*^1^-(2-aminoethyl)ethane-1,2-diamine] and 0.50 g (4.0 mmol) of ligand L_3_ [2-(pyridin-2-yl)ethan-1-amine], 15 mL of a methanolic solution containing 1.51 g (4.0 mmol) of copper(ii) perchlorate hexahydrate, [Cu(H_2_O)_6_](ClO_4_)_2_, was added dropwise in a 100-mL round-bottom flask. After stirring the resulting mixture for 1 hour at room temperature, a blue-coloured precipitate of complex 2 was obtained. The precipitate was filtered, and the residue was recrystallized in a methanol–water mixture to obtain blue-coloured crystals. Yield: 1.28 g (∼88%). Elemental analyses: Calcd for C_11_H_23_N_5_Cl_2_O_8_Cu (%): C, 27.09; H, 4.75; N, 14.36. Found (%): C, 27.05; H, 4.73; N, 14.35. FT-IR (KBr pellet): 3435, 3221, 3111, 2925, 1622, 1111 cm^−1^. Magnetic moment: 1.68 µB. The molar conductance of complex 2 in an acetonitrile solution was found to be 208 S cm^2^ mol^−1^.

### 4.4 Synthesis of [Zn(L_1_H)(L_2_)](ClO_4_)_2_

To a methanolic solution containing 0.28 g (2.7 mmol) of ligand L_1_H [*N*^1^-(2-aminoethyl)ethane-1,2-diamine] and 0.30 g (2.7 mmol) of ligand L_2_ (pyridine-2-ylmethanamine), 15 mL of a methanolic solution containing 1.03 g (2.7 mmol) of zinc(ii) perchlorate hexahydrate, [Zn(H_2_O)_6_](ClO_4_)_2_, was added dropwise in a 100-mL round-bottom flask. After stirring the resulting mixture for 1 hour at room temperature, a white precipitate of [Zn(L_1_H)(L_2_)](ClO_4_)_2_ was obtained. The precipitate was filtered, and the residue was recrystallized in a methanol–water mixture to obtain colourless crystals. Yield: 0.86 g (∼81%). Elemental analyses: Calcd for C_10_H_21_N_5_Cl_2_O_8_Zn (%): C, 25.26; H, 4.45; N, 14.73. Found (%): C, 25.24; H, 4.44; N, 14.75. FT-IR (KBr pellet): 3431, 3234, 3119, 2915, 1612, 1380, 1109 cm^−1^.

### 4.5 Synthesis of [Zn(L_1_H)(L_3_)](ClO_4_)_2_

To a methanolic solution containing 0.25 g (2.5 mmol) of ligand L_1_H [*N*^1^-(2-aminoethyl)ethane-1,2-diamine] and 0.30 g (2.5 mmol) of ligand L_3_ [2-(pyridin-2-yl)ethan-1-amine], 15 mL of a methanolic solution containing 0.91 g (2.5 mmol) of zinc(ii) perchlorate hexahydrate, [Zn(H_2_O)_6_](ClO_4_)_2_, was added dropwise in a 100-mL round-bottom flask. After stirring the resulting mixture for 1 hour at room temperature, a white precipitate of [Zn(L_1_H)(L_3_)](ClO_4_)_2_ was obtained. The precipitate was filtered, and the residue was recrystallized in a methanol–water mixture to obtain colourless crystals. Yield: 0.78 g (∼84%). Elemental analyses: Calcd for C_11_H_23_N_5_Cl_2_O_8_Zn (%): C, 26.98; H, 4.74; N, 14.30. Found (%): C, 26.97; H, 4.72; N, 14.32. FT-IR (KBr pellet): 3486, 3221, 3112, 2909, 1591, 1442, 1116 cm^−1^.

## Conflicts of interest

There are no conflicts to declare.

## Supplementary Material

RA-016-D6RA01803C-s001

RA-016-D6RA01803C-s002

## Data Availability

CCDC 2512656–2512659 contain the supplementary crystallographic data for this paper.^44*a*–*d*^ The data supporting this article have been included as part of the supplementary information (SI). Supplementary information: experimental procedures, FT-IR, UV-Visible. See DOI: https://doi.org/10.1039/d6ra01803c.
