# Supplementary material for: Electrocatalytic water oxidation by Cu(ii) complexes: influence of ternarification and chelate ring size
Source: RSC Adv. 2026 Jul 3;16(35):36642–9. doi: 10.1039/d6ra01803c (PMC13330980; doi:10.1039/d6ra01803c)
Supplement: RA-016-D6RA01803C-s001 [file RA-016-D6RA01803C-s001.pdf]

Supplementary Information (SI) for RSC Advances.
This journal is © The Royal Society of Chemistry 2026

Supporting Information

For

Electrocatalytic water oxidation by Cu (II) complexes: Influence of ternarification and chelate ring size

Pranjal Das,[a,b] Swati Basak,[a,b] Baisali Hazarika[a,b], and Apurba Kalita*[a]

[a]*Department of Chemistry, B. Borooah College, Guwahati, Assam 781007, India*

[b]*Department of Chemistry, Gauhati University, Guwahati 781014, Assam, India*

Table of Contents:

Sl. No.	Contents		Page No.
**1**	Experimental Procedures		**10-11**
**2**	Electron count in redox peak using Laviron equations		**11**
**3**	Calculation of Faradic efficiency		**11-12**
**4**	Calculation of Turn-Over-Number (TON)		**12**
**5**	Computational Study (calculating redox potential)		**12-13**
**6**	Detection of peroxide intermediates generated during CPE experiment		**14**
**7**	**Fig. S1**	ORTEP diagram (50% thermal ellipsoid plot) of **(a)** complex **$[Zn(L_1H)(L_2)](ClO_4)_2$** and **(b)** complex **$[Zn(L_1H)(L_3)](ClO_4)_2$**. Counter ions, hydrogen atoms and solvent molecules are removed for clarity.	**15**
**8**	**Fig. S2**	FT-IR spectra of **(a)** complex **1** and **(b)** complex **2** in KBr.	**15**
**9**	**Fig. S3**	FT-IR spectra of **(a)** complex **$[Zn(L_1H)(L_2)](ClO_4)_2$** and **(b)** complex **$[Zn(L_1H)(L_3)](ClO_4)_2$** in KBr.	**15**
**10**	**Fig. S4**	PXRD patterns of **(a)** complex **1** and **(b)** complex **2**.	**16**
**11**	**Fig. S5**	**(a)** UV-visible spectra of 1 mM solution of complex **1** (red line) and complex **2** (blue line) in 0.1 M neutral phosphate buffer. **(b)** UV-visible spectra of 0.05 mM solution of complex **1** (red line) and complex **2** (blue line) in 0.1 M neutral phosphate buffer.	**16**
**12**	**Fig. S6**	**(a)** UV-visible spectra of 1 mM solution of complex **1** in water (red line) and in 0.1 M neutral phosphate buffer (blue line). **(b)** UV-visible spectra of 0.05 mM solution of complex **1** in water (red line) and in 0.1 M neutral phosphate buffer (blue line)	**17**
**13**	**Fig. S7**	**(a)** UV-visible spectra of 1 mM solution of complex **2** in water (red line) and in 0.1 M neutral phosphate buffer (blue line). **(b)** UV-visible spectra of 0.05 mM solution of complex **2** in water (red line) and in 0.1 M neutral phosphate buffer (blue line)	**17**
**14**	**Fig. S8**	**(a)** UV-visible spectra of 1 mM solution of complex **1** recorded at different time interval in 0.1 M neutral phosphate buffer. **(b)** UV-visible spectra of 0.05 mM solution of complex **1** recorded at different time interval in 0.1 M neutral phosphate buffer.	**18**

**15**	**Fig. S9**	**(a)** UV-visible spectra of 1 mM solution of complex **2** recorded at different time interval in 0.1 M neutral phosphate buffer. **(b)** UV-visible spectra of 0.05 mM solution of complex **2** recorded at different time interval in 0.1 M neutral phosphate buffer.	**18**
**16**	**Fig. S10**	Cyclic voltammogram of 1 mM solution of **(a)** complex **1** and **(b)** complex **2** in 0.1 M neutral phosphate at 100 mVs^{-1} scan rate. The onset potentials for water oxidation located near 1.24 V and 1.30 V vs. NHE for complexes **1** and **2**, respectively.	**19**
**17**	**Fig. S11**	**(a)** Cyclic voltamograms of complex **1** at 20 (black), 30 (red), 40 (green), 50 (blue), 60 (cyan), 70 (magenta), 80 (purple), 90 (royal) and 100 (violet) mVs^{-1} scan rates in 0.1 M neutral phosphate buffer. **(b)** Plot of –ln υ vs. potential of complex **1** for cathodic current of Cu(II)-Cu(I) couple (red dot), anodic current of Cu(II)-Cu(I) couple (green dot), 2nd anodic peak (blue dot), 3rd anodic peak (cyan dot) and 4th anodic peak (purple dot).	**19**
**18**	**Fig. S12**	**(a)** Cyclic voltamograms of complex **2** at 20 (black), 30 (red), 40 (green), 50 (blue), 60 (cyan), 70 (magenta), 80 (purple), 90 (royal) and 100 (violet) mVs^{-1} scan rates in 0.1 M neutral phosphate buffer. **(b)** Plot of –ln υ vs. potential of complex **2** for cathodic current of Cu(II)-Cu(I) couple (red dot), anodic current of Cu(II)-Cu(I) couple (green dot), 2nd anodic peak (blue dot) and 3rd anodic peak (cyan dot).	**20**
**19**	**Fig. S13**	**(a)** Cyclic voltammograms and **(b)** Differential Pulse voltammograms of complex **1** (blue), and analogous Zn complex, **$[Zn(L_1H)(L_2)](ClO_4)_2$** (red) in 0.1 M neutral phosphate buffer recorded with a glassy carbon (GC) working electrode, a Ag/AgCl reference electrode and a Pt counter electrode, scan rate 100 mVs^{-1}.	**20**
**20**	**Fig. S14**	**(a)** Cyclic voltammograms and **(b)** Differential Pulse voltammograms of complex **2** (blue), and analogous Zn complex, **$[Zn(L_1H)(L_3)](ClO_4)_2$** (red) in 0.1 M neutral phosphate buffer recorded with a glassy carbon (GC) working electrode, a Ag/AgCl reference electrode and a Pt counter electrode, scan rate	**21**

		100 mVs^{-1}.	
**21**	**Fig. S15**	**(a)** Cyclic voltammograms and **(b)** Differential Pulse voltammograms of ligand **L_1H**, N^1-(2-aminoethyl)ethane-1,2-diamine (black), ligand **L_2**, Pyridin-2-ylmethanamine (red) and ligand **L_3**, 2-(Pyridin-2-yl)ethan-1-amine (blue) in 0.1 M neutral phosphate buffer recorded with a glassy carbon (GC) working electrode, a Ag/AgCl reference electrode and a Pt counter electrode, scan rate 100 mVs^{-1}.	**21**
**22**	**Fig. S16**	DFT optimized structure of **(a)** complex **1** in doublet state, **(b)** complex **1** in quartet state (unimportant hydrogen atoms are not shown for clarity).	**22**
**23**	**Fig. S17**	DFT optimized structure of **(a)** complex **1a** in singlet state, **(b)** complex **1a** in triplet state (unimportant hydrogen atoms are not shown for clarity).	**22**
**24**	**Fig. S18**	DFT optimized structure of **(a)** complex **1b** in doublet state, **(b)** complex **1b** in quartet state (unimportant hydrogen atoms are not shown for clarity).	**23**
**25**	**Fig. S19**	DFT optimized structure of Transition State (**TS**) of complex **1** **(a)** in singlet state, **(b)** in triplet state (unimportant hydrogen atoms are not shown for clarity).	**23**
**26**	**Fig. S20**	DFT optimized structure of **(a)** complex **1c** in singlet state, **(b)** complex **1c** in triplet state (unimportant hydrogen atoms are not shown for clarity).	**24**
**27**	**Fig. S21**	DFT optimized structure of **(a)** complex **2** in doublet state, **(b)** complex **2** in quartet state (unimportant hydrogen atoms are not shown for clarity).	**24**
**28**	**Fig. S22**	DFT optimized structure of **(a)** complex **2a** in singlet state, **(b)** complex **2a** in triplet state (unimportant hydrogen atoms are not shown for clarity).	**25**
**29**	**Fig. S23**	DFT optimized structure of **(a)** complex **2b** in doublet state, **(b)** complex **2b** in quartet state (unimportant hydrogen atoms are not shown for clarity).	**25**
**30**	**Fig. S24**	DFT optimized structure of Transition State (**TS**) of complex **2**	**26**

		**(a)** in singlet state, **(b)** in triplet state (unimportant hydrogen atoms are not shown for clarity).	
**31**	**Fig. S25**	DFT optimized structure of **(a)** complex **2c** in singlet state, **(b)** complex **2c** in triplet state (unimportant hydrogen atoms are not shown for clarity).	**26**
**32**	**Fig. S26**	Energy diagram (ΔG_{298K} in kcal/mol) for water oxidation catalysed by complex **1**. The reference potential of 0.9 V is used to set up the thermodynamics. To construct the energy diagram for the full catalytic cycle, the total exergonicity of 39.018 $kcalmol^{-1}$ derived from the experimental over-potential (onsite potential of 1.24 V, over-potential 0.423 V at pH 7) was used.	**27**
**33**	**Fig. S27**	Energy diagram (ΔG_{298K} in kcal/mol) for water oxidation catalysed by complex **2**. The reference potential of 0.9 V is used to set up the thermodynamics. To construct the energy diagram for the full catalytic cycle, the total exergonicity of 44.552 $kcalmol^{-1}$ derived from the experimental over-potential (onsite potential of 1.30 V, over-potential 0.483 V at pH 7) was used.	**28**
**34**	**Fig. S28**	**(a)** Differential pulse voltammogram (DPV) of complex **1** in 0.1 M neutral phosphate buffer, **(b)** Proposed catalytic cycle of complex **1**, **(c)** Assignment of experimental and theoretical redox potentials to the proposed electrochemical steps in the catalytic cycle of complex **1**.	**29**
**35**	**Fig. S29**	**(a)** Differential pulse voltammogram (DPV) of complex **2** in 0.1 M neutral phosphate buffer, **(b)** Proposed catalytic cycle of complex **2**, **(c)** Assignment of experimental and theoretical redox potentials to the proposed electrochemical steps in the catalytic cycle of complex **2**.	**30**
**36**	**Fig. S30**	UV-visible spectra obtained after **(a)** addition of HPR and AR on buffer solution after electrolysis, **(b)** addition of HPR and AR on fresh solution of complex **1**, **(c)** addition of HPR and AR on solution of complex **1** after electrolysis, **(d)** addition of HPR and AR on buffer solution containing 100 μL of H_2O_2.	**31**
**37**	**Fig. S31**	UV-visible spectra obtained after **(a)** addition of HPR and AR on	**32**

		buffer solution after electrolysis, **(b)** addition of HPR and AR on fresh solution of complex **2**, **(c)** addition of HPR and AR on solution of complex **2** after electrolysis, **(d)** addition of HPR and AR on buffer solution containing 100 μL of H_2O_2.	
**38**	**Fig. S32**	**(a)** Cyclic voltammograms of complex **1** at 0.08 (black), 0.16 (red), 0.24 (green), 0.32 (blue), 0.40 (cyan), 0.48 (magenta), 0.56 (yellow), 0.64 (navy), 0.72 (purple), 0.80 (royal) and 0.88 mM (violet) concentration in 0.1 M neutral phosphate buffer. **(b)** Catalytic current at 1.38 V vs. NHE for complex **1** as a function of the catalyst concentration from 0.08 mM to 0.88 mM in 0.1 M neutral phosphate buffer.	**33**
**39**	**Fig. S33**	**(a)** Cyclic voltammograms of complex **2** at 0.08 (black), 0.16 (red), 0.24 (green), 0.32 (blue), 0.40 (cyan), 0.48 (magenta), 0.56 (yellow), 0.64 (navy), 0.72 (purple), 0.80 (royal) and 0.88 mM (violet) concentration in 0.1 M neutral phosphate buffer. **(b)** Catalytic current at 1.35 V vs. NHE for complex **2** as a function of the catalyst concentration from 0.08 mM to 0.88 mM in 0.1 M neutral phosphate buffer.	**33**
**40**	**Fig. S34**	**(a)** Cyclic voltammograms of complex **1** in the range 0.28 to -0.21 V vs. NHE at 20 (black), 30 (red), 40 (green), 50 (blue), 60 (cyan), 70 (magenta), 80 (purple), 90 (royal) and 100 (violet) mVs^{-1} scan rate. **(b)** Dependence of the peak current for the Cu^{II}/Cu^{I} couple of complex **1** on the square root of scan rate with standard three electrode system in 0.1 M neutral phosphate buffer. D_{Cu} = 6.86 x 10^{-6} cm^2/s.	**34**
**41**	**Fig. S35**	**(a)** Cyclic voltammograms of complex **2** in the range 0.36 to -0.24 V vs. NHE at 20 (black), 30 (red), 40 (green), 50 (blue), 60 (cyan), 70 (magenta), 80 (purple), 90 (royal) and 100 (violet) mVs^{-1} scan rate. **(b)** Dependence of the peak current for the Cu^{II}/Cu^{I} couple of complex **2** on the square root of scan rate with standard three electrode system in 0.1 M neutral phosphate buffer. D_{Cu} = 8.13 x 10^{-6} cm^2/s.	**34**
**42**	**Fig. S36**	Plot of current density vs. time recorded during 4 hour of bulk	**35**

		electrolysis with (blue line) and without (red line) **(a)** complex **1** and **(b)** complex **2** in 0.1 M neutral phosphate buffer using ITO working electrode (area 4 cm^2), Ag/AgCl reference electrode and Pt counter electrode at 1.38 V vs. NHE and 1.35 V vs. NHE respectively.	
**43**	**Fig. S37**	Cyclic voltammograms of **(a)** complex **1** and **(b)** complex **2** recorded before (blue) and after (red) 4 hour of bulk electrolysis at 1.38 V and 1.35 V vs. NHE respectively in 0.1 M neutral phosphate buffer using glassy carbon (GC) as working electrode (area 0.07 cm^2), Ag/AgCl as reference electrode and Pt as counter electrode. Scan rate, 100 mVs^{-1}.	**35**
**44**	**Fig. S38**	UV-visible spectra of **(a)** complex **1** and **(b)** complex **2** recorded before (blue) and after (red) 4 hour of bulk electrolysis at 1.38 V and 1.35 V vs. NHE respectively in 0.1 M neutral phosphate buffer.	**36**
**45**	**Fig. S39**	Cyclic voltammograms recorded in 0.1 M neutral phosphate buffer in the absence of **(a)** complex **1** and **(b)** complex **2** with fresh (blue) and used (red) ITO working electrode (area 4 cm^2), a Ag/AgCl reference electrode and a Pt counter electrode, scan rate. 100 mVs^{-1}.	**36**
**46**	**Fig. S40**	FE-SEM and EDX plot of **(a)** fresh ITO working electrode **(b)** ITO working electrode after 4 hour of bulk electrolysis of complex **1** and **(c)** ITO working electrode after 4 hour of bulk electrolysis of complex **2** in 0.1 M neutral phosphate buffer.	**37**
**47**	**Fig. S41**	Consecutive cyclic voltamograms of **(a)** complex **1** and **(b)** complex **2** recorded using glassy carbon working electrode (area 0.07 cm^2) at 100 mVs^{-1} scan rates in 0.1 M neutral phosphate buffer.	**37**
**48**	**Fig. S42**	Dynamic Light Scattering (DLS) size distribution of complex **1** measured in 0.1 M neutral phosphate buffer **(a)** before and **(b)** after 500 consecutive cycle cyclic voltammetry experiment.	**38**
**49**	**Fig. S43**	Dynamic Light Scattering (DLS) size distribution of complex **2** measured in 0.1 M neutral phosphate buffer **(a)** before and **(b)**	**39**

		after 500 consecutive cycle cyclic voltammetry experiment.	
**50**	**Table S1**	Crystal data and structure refinement parameters for complexes **1** and **2**.	**40**
**51**	**Table S2**	Crystal data and structure refinement parameters for complexes **$[Zn(L_1H)(L_2)](ClO_4)_2$** and **$[Zn(L_1H)(L_3)](ClO_4)_2$**.	**41**
**52**	**Table S3**	Gibbs free energies of intermediates and transition state of complex **1** in aqueous solution.	**42**
**53**	**Table S4**	Gibbs free energies of intermediates and transition state of complex **2** in aqueous solution.	**43**
**54**	**Table S5**	Coordinate file of the DFT optimized structure of complex **1** in doublet state.	**44**
**55**	**Table S6**	Coordinate file of the DFT optimized structure of complex **1** in quartet state.	**45**
**56**	**Table S7**	Coordinate file of the DFT optimized structure of complex **1a** in singlet state.	**46**
**57**	**Table S8**	Coordinate file of the DFT optimized structure of complex **1a** in triplet state.	**47**
**58**	**Table S9**	Coordinate file of the DFT optimized structure of complex **1b** in doublet state.	**48**
**59**	**Table S10**	Coordinate file of the DFT optimized structure of complex **1b** in quartet state.	**49**
**60**	**Table S11**	Coordinate file of the DFT optimized structure of Transition State (**TS**) of complex **1** in singlet state.	**50**
**61**	**Table S12**	Coordinate file of the DFT optimized structure of Transition State (**TS**) of complex **1** in triplet state.	**51**
**62**	**Table S13**	Coordinate file of the DFT optimized structure of complex **1c** in singlet state.	**52**
**63**	**Table S14**	Coordinate file of the DFT optimized structure of complex **1c** in triplet state.	**53**
**64**	**Table S15**	Coordinate file of the DFT optimized structure of complex **2** in doublet state.	**54**
**65**	**Table S16**	Coordinate file of the DFT optimized structure of complex **2** in quartet state.	**55**

**66**	**Table S17**	Coordinate file of the DFT optimized structure of complex **2a** in singlet state.	**56**
**67**	**Table S18**	Coordinate file of the DFT optimized structure of complex **2a** in triplet state.	**57**
**68**	**Table S19**	Coordinate file of the DFT optimized structure of complex **2b** in doublet state.	**58**
**69**	**Table S20**	Coordinate file of the DFT optimized structure of complex **2b** in quartet state.	**59**
**70**	**Table S21**	Coordinate file of the DFT optimized structure of Transition State (**TS**) of complex **2** in singlet state.	**60**
**71**	**Table S22**	Coordinate file of the DFT optimized structure of Transition State (**TS**) of complex **2** in triplet state.	**61**
**72**	**Table S23**	Coordinate file of the DFT optimized structure of complex **2c** in singlet state.	**62**
**73**	**Table S24**	Coordinate file of the DFT optimized structure of complex **2c** in triplet state.	**63**
**74**	**Table S25**	Catalytic performance comparison between ternary complexes in this work and the reported pyridine based and aliphatic amine ligand containing copper complexes for water oxidation.	**64-68**
**75**	**References**		**69-73**

Experimental Procedures:

All reagents and solvents were purchased from commercial sources and were of reagent grade. UV-visible spectra were recorded on Cary-60 UV-Visible spectrophotometer. FT-IR spectra were recorded on a Cary 630 spectrophotometer with sample prepared as KBr pellets. The magnetic moment of the complex were measured on a Cambridge magnetic balance. Conductivity measurements were recorded using a Eutech instrument CON 700. Elemental analyses were carried out on a Thermo Scientific Flashmart Analyzer. Electrochemical measurements were made using CHI 7035E bipotentiostat. Glassy carbon working electrode, Pt wire auxiliary electrode, and Ag/AgCl reference electrode were used in a three-electrode configuration. DLS measurements were recorded in Litesizer 500 instrument. A Carl Zeiss Supra 55 electron microscope was used for Field Emission Scanning Electron Microscope (FE-SEM) studies after Au coating. A 20 kV electron beam used for collection of EDX spectra and atomic mapping images. Powder X-ray diffraction (PXRD) data were collected from 5 to 50^0 2Θ using a Bruker-D8 Advance X-ray dffractometer.

The single crystal data were collected on a Bruker Smart Apex Duo diffractometer, utilizing MoK_α radiation (λ = 0.71073 Å). The structures were initially solved with the direct method and further refined by employing full-matrix least squares based on F^2, using SHELXL-2014/7 software integrated into Apex 3 suite.[S1] All the hydrogen positions were initially located in the difference Fourier maps, and for the final refinement, the hydrogen atoms were placed in geometrically ideal positions and refined in the riding mode.[S2] Final refinement included atomic positions for all the atoms, anisotropic thermal parameters for all the non-hydrogen atoms, and isotropic thermal parameters for all the hydrogen atoms were carried out using Olex2 1.2 package of programs.[S3,S4] Structural illustrations have been drawn with ORTEP-3 for Windows. CCDC: 2512656, 2512657, 2512658, and 2512659 contains the

crystallographic data for this paper. These data can be obtained free of charge from The Cambridge Crystallographic Data Centre (CCDC) via www.ccdc.cam.ac.uk/data_request/cif.

Electron count in redox peak using Laviron equations:

To obtain the number of electrons transferred in a redox peak, Laviron equations are used. The Laviron equations are given as below:

$$E_{p,c} = E^{\Theta} - (RT/\alpha nF)\ \ln(\alpha n v/RTk) = C - (RT/\alpha nF)\ \ln(v) \quad (1)$$

$$E_{p,a} = E^{\Theta} + [RT/(1-\alpha)nF]\ \ln[(1-\alpha)nv/RTk] = C + [RT/(1-\alpha)nF]\ \ln(v) \quad (2)$$

$$E_{p/2} = C + [RT/(1-\alpha)nF]\ \ln(v) \quad (3)$$

Where, E^{Θ} is the standard potential, $E_{p,c}$is the potential for cathodic peak, $E_{p,a}$is the potential for anodic peak, R is the ideal gas constant, T is temperature, F is Faraday constant, k is the rate constant of the electrochemical reaction (s^{-1}) and C is the constant.

The α value is calculated using equation (1) and equation (2) for a reversible couple from the slope of $E_{p,a}$vs. -ln ν and $E_{p,c}$ vs. -ln ν. With the calculated value of α, number of electron (n) can be calculated. For the irreversible peak, number of electron (n) can be calculated using equation (3) from the slope of $E_{1/2}$ vs. -ln ν. The value of $E_{p,a}$, $E_{p,c}$ and $E_{1/2}$ are obtained from the cyclic voltammograms of the complexes at different scan rates.

Calculation of Faradic efficiency:

Bulk electrolysis was performed in a two-compartment, three-electrode cell (the volume for each cell is 90 mL). The counter electrode compartment, containing a platinum mesh electrode, was separated from the working electrode compartment by Nafion N-117 membrane. An Ag/AgCl reference electrode (sat. KCl) was placed in the working electrode compartment. The O_2 product analysis was determined using a calibrated Ocean Optics FOXY probe. The electrolyte was degassed by bubbling with high purity of argon for 1 hour. After recording the partial pressure of O_2 for 4 hour in the absence of an applied potential,

electrolysis was initiated at 1.38 V and 1.35 V vs. NHE for complexes **1** and **2** respectively. Electrolysis with O_2 sensing was continued for 4 hour.

% Faradic efficiency = [(Actual amount of oxygen produced) / (Theoretically calculated amount of oxygen)] X 100

The theoretically calculated amount of oxygen during Bulk Electrolysis (BE) experiment can be obtained using the following relation:

$$\text{Theoretical yield of oxygen (in mol L}^{-1}\text{)} = (Q / 4FV)$$

Where,

Q = Total charge passed during Bulk Electrolysis (BE) experiment in coulomb.

F = Faraday constant

V= Volume of the complex solution used for bulk electrolysis (BE) experiment in Litter

Calculation of Turn-Over-Number (TON):

The Turn-Over-Number (TON) during Control Potential Electrolysis (CPE) experiment can be calculated using the following relation:

$$\mathbf{TON} = \frac{\textbf{No. of moles } \mathbf{O_2} \textbf{ generated during CPE Experiment}}{\textbf{No. of moles of catalyst used}}$$

Computational Study (Calculating redox potentials):

The geometry optimizations in the present study were performed using using the B3LYP functional, as implemented in the Gaussian 09 package.[S5] The LanL2DZ basis set was employed for Cu atoms, while the 6-31G** basis set was used for C, H, N, and O atoms. Solvation effects in aqueous solution were modeled using the SMD continuum solvation model.

Free energy of proton in solution, $G_s(H^+)$, is calculated from the following relation:

$$G_s(H^+) = G_g(H^+) + \Delta G_{solv}(H^+) + \Delta G^*$$

Where, $G_g(H^+)$ is the gas-phase free energy of proton (-6.3 $kcalmol^{-1}$) and $\Delta G_{solv}(H^+)$ is the solvation free energy of proton [in water $\Delta G_{solv}(H^+)$ = -265.9 $kcalmol^{-1}$]. ΔG^* is the free energy change associated with the conversion from a standard state of 1 M in the solvent phase and 1 atm in gas phase, to the desired concentration in both phases. ΔG^* values are derived from the following expression:

$$\Delta G^* = RT \ln(24.4*c)$$

Where, R is the universal gas constant (1.987 cal mol^{-1} K^{-1}), T is the temperature in Kelvin and c the concentration in $mol \cdot L^{-1}$.

The standard Gibbs free energy of any species '*i*' in aqueous solution, $G^o_{aq}(i)$, is expressed as

$$G^o_{aq}(i) = G^o_g(i) + G^o_{solv}(i) + \Delta G^*$$

Where, $G^o_g(i)$ is the gas-phase free energy of the species '*i*', $G^o_{solv}(i)$ is the solvation free energy of transferring the solute from the gas phase (1M) to a 1M aqueous solution at 298.15 K.

Redox potentials relative to a reference electrode is calculated by:

$$E^o = (-\Delta G^o / F) - E_{ref}$$

Where, ΔG^o is the standard free energy change associated with the redox reaction, F is the Faraday constant (23.061 $kcal \cdot mol^{-1}$). In the standard protocol for the calculation of one electron redox potential, the experimental absolute redox potential of SHE is used (E_{ref} = 4.281 V). At experimental conditions, the measured redox potentials are related to E^o (standard conditions, pH = 0) through the Nernst equation:

$$E = E^o - (0.059*p^H)$$

To construct the energy diagrams for the full catalytic cycle, the total exergonicity of 39.018 kcal/mol and 44.552 kcal/mol derived from the experimental over-potential (onset potentials of 1.24 V and 1.30 V, over-potentials 0.423 V and 0.483 V vs. NHE at pH 7.0) were used for complexes **1** and **2** respectively.

Detection of peroxide intermediates generated during CPE experiment:

Ampliflu Red (AR) was dissolved in dimethyl sulfoxide (DMSO) to prepare a 0.4 mg mL^{-1} stock solution. Horseradish peroxidase (HRP) was prepared separately in 0.1 M neutral phosphate buffer solution at the same concentration.

Controlled potential electrolysis (CPE) was carried out using complexes **1** and **2** (0.5 mM) in neutral phosphate buffer solution at applied potential of 1.38 V and 1.35 V vs. NHE respectively. Electrolysis was performed in a two-compartment electrochemical cell separated by a Nafion membrane, employing an indium tin oxide (ITO) electrode (4 cm^2) as the working electrode.

After 4 hour of electrolysis under constant stirring, 0.3 mL of the electrolyte was withdrawn and treated sequentially with HRP solution (0.5 mL) followed by AR solution (0.5 mL). In the presence of peroxide intermediates generated during electrolysis, HRP catalyses the oxidation of non-fluorescent AR to the strongly coloured and fluorescent Resorufin, resulting in an immediate colour change from blue to pink.

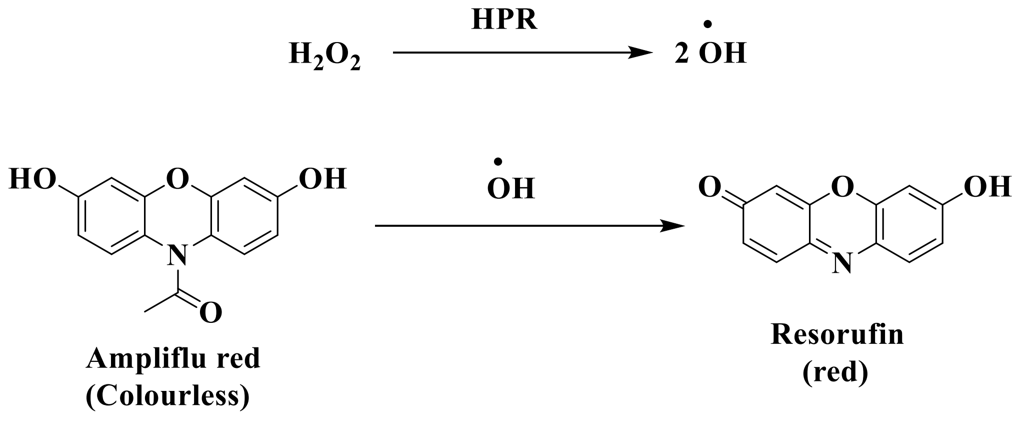

(a) (b)

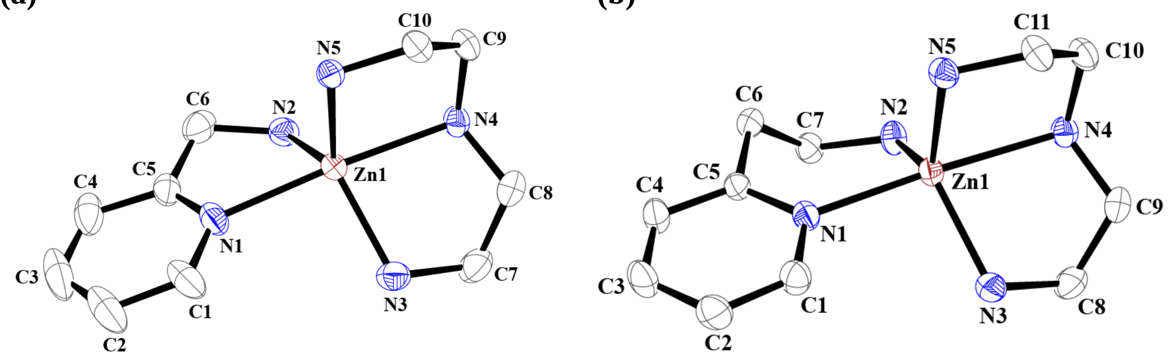

Fig. S1 ORTEP diagram (50% thermal ellipsoid plot) of **(a)** complex **[Zn(L$_1$H)(L$_2$)](ClO$_4$)$_2$** and **(b)** complex **[Zn(L$_1$H)(L$_3$)](ClO$_4$)$_2$**. Counter ions, hydrogen atoms and solvent molecules are removed for clarity.

(a) (b)

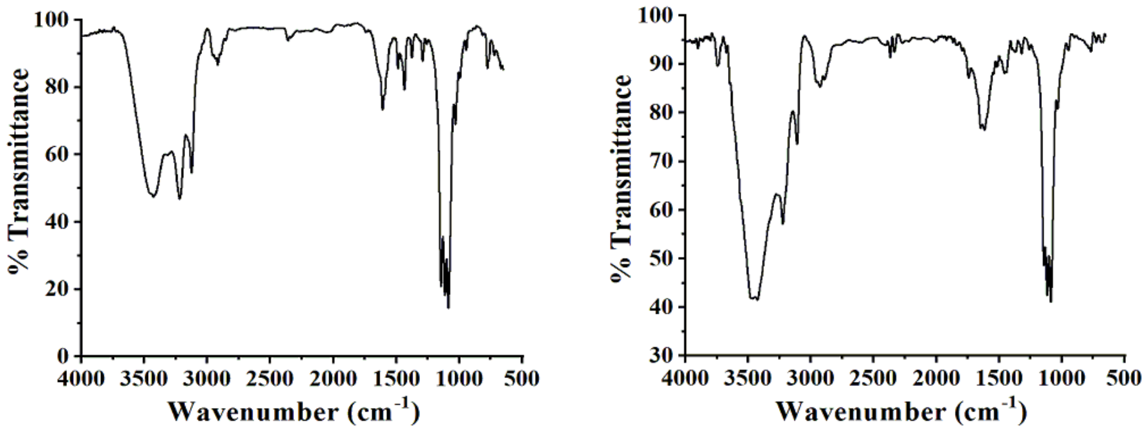

Fig. S2 FT-IR spectra of **(a)** complex **1** and **(b)** complex **2** in KBr.

(a) (b)

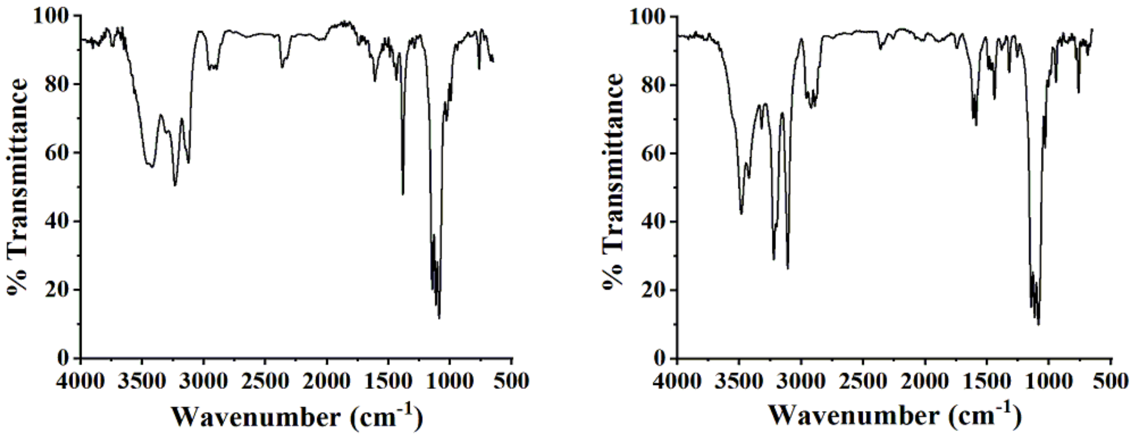

Fig. S3 FT-IR spectra of **(a)** complex **[Zn(L$_1$H)(L$_2$)](ClO$_4$)$_2$** and **(b)** complex **[Zn(L$_1$H)(L$_3$)](ClO$_4$)$_2$** in KBr.

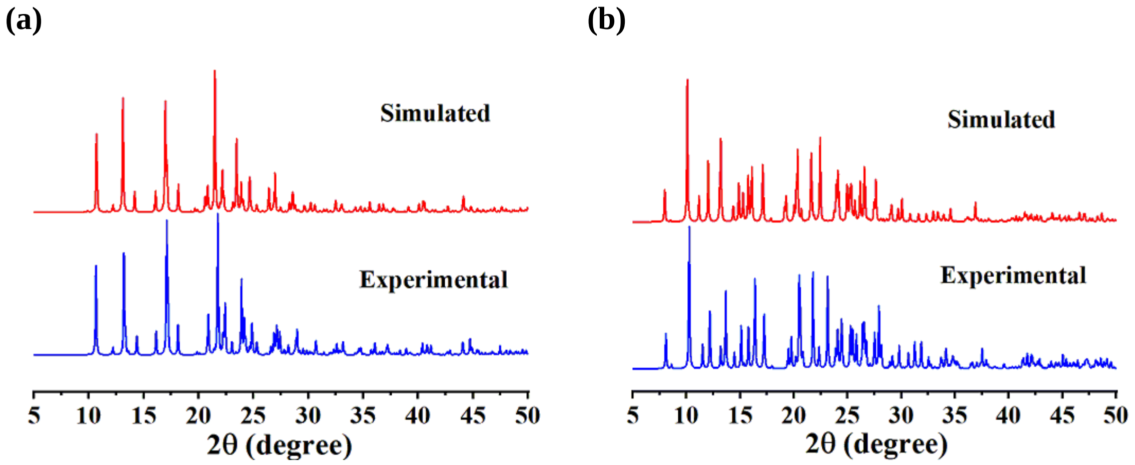

Fig. S4 PXRD patterns of **(a)** complex **1** and **(b)** complex **2**.

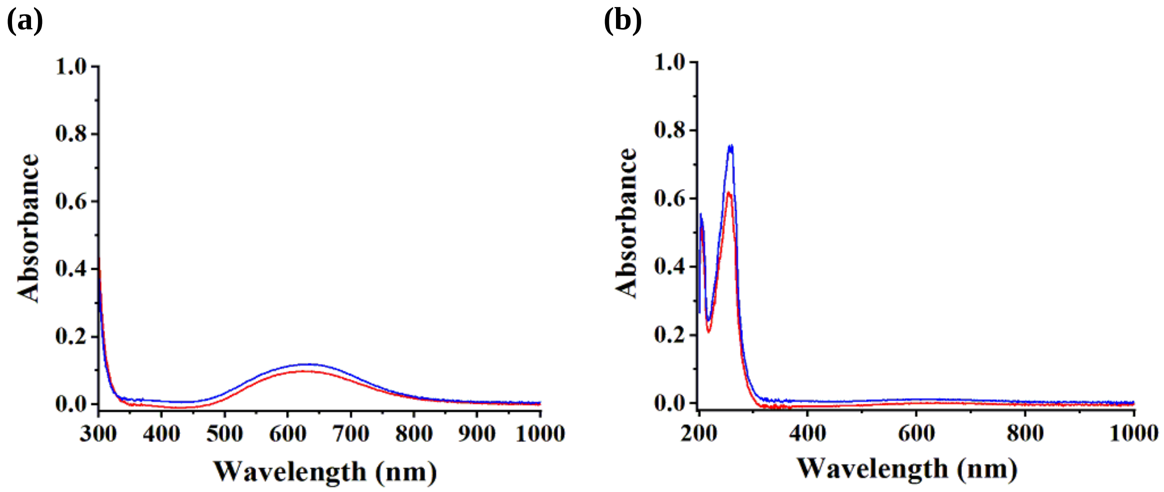

Fig. S5 **(a)** UV-visible spectra of 1 mM solution of complex **1** (red line**)** and complex **2** (blue line) in 0.1 M neutral phosphate buffer. **(b)** UV-visible spectra of 0.05 mM solution of complex **1** **(**red line**)** and complex **2** (blue line) in 0.1 M neutral phosphate buffer.

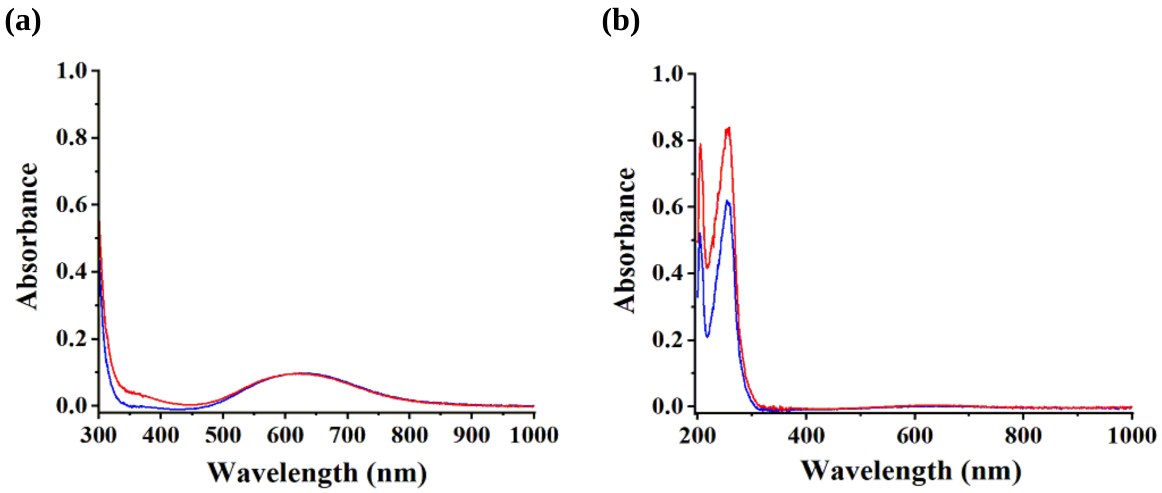

Fig. S6 **(a)** UV-visible spectra of 1 mM solution of complex **1** in water (red line) and in 0.1 M neutral phosphate buffer (blue line). **(b)** UV-visible spectra of 0.05 mM solution of complex **1** in water (red line) and in 0.1 M neutral phosphate buffer (blue line)

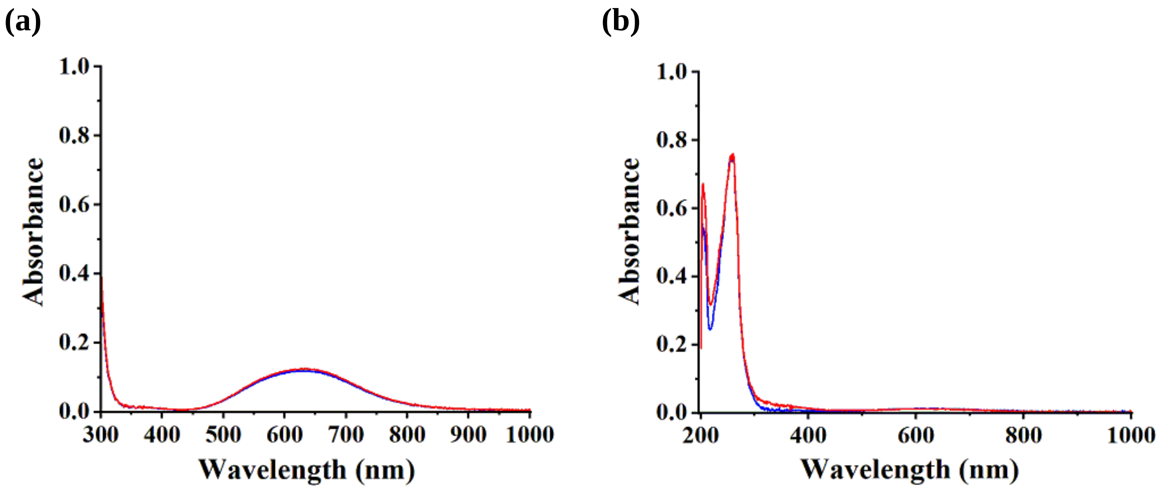

Fig. S7 **(a)** UV-visible spectra of 1 mM solution of complex **2** in water (red line) and in 0.1 M neutral phosphate buffer (blue line). **(b)** UV-visible spectra of 0.05 mM solution of complex **2** in water (red line) and in 0.1 M neutral phosphate buffer (blue line)

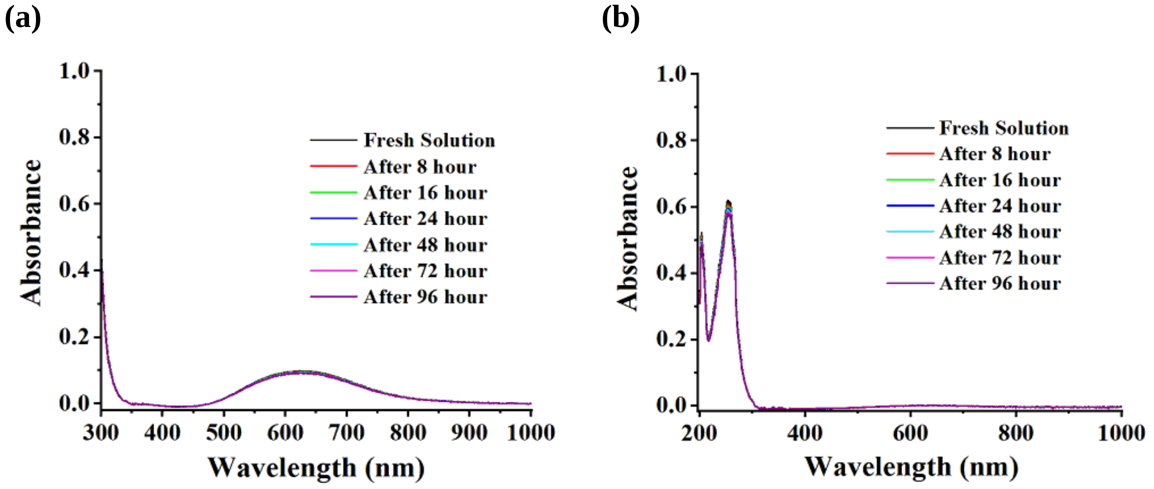

Fig. S8 **(a)** UV-visible spectra of 1 mM solution of complex **1** recorded at different time interval in 0.1 M neutral phosphate buffer. **(b)** UV-visible spectra of 0.05 mM solution of complex **1** recorded at different time interval in 0.1 M neutral phosphate buffer.

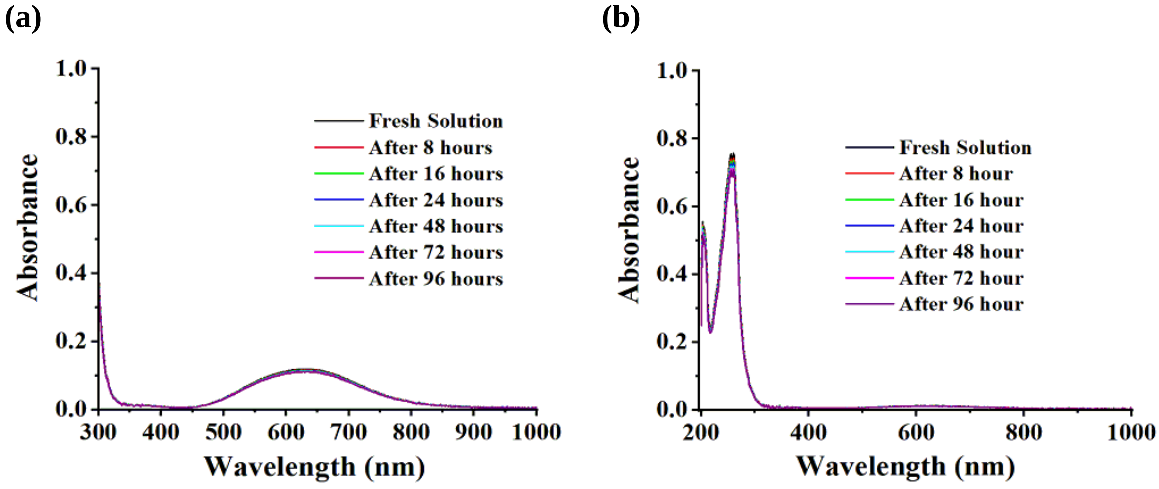

Fig. S9 **(a)** UV-visible spectra of 1 mM solution of complex **2** recorded at different time interval in 0.1 M neutral phosphate buffer. **(b)** UV-visible spectra of 0.05 mM solution of complex **2** recorded at different time interval in 0.1 M neutral phosphate buffer.

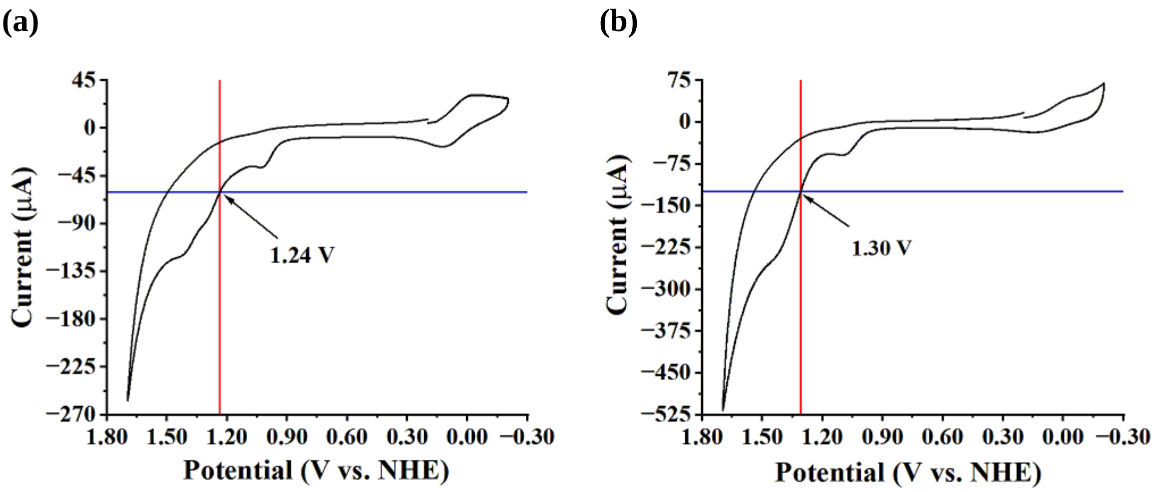

Fig. S10 Cyclic voltammogram of 1 mM solution of **(a)** complex **1** and **(b)** complex **2** in 0.1 M neutral phosphate at 100 mVs^{-1} scan rate. The onset potentials for water oxidation located near 1.24 V and 1.30 V vs. NHE for complexes **1** and **2**, respectively.

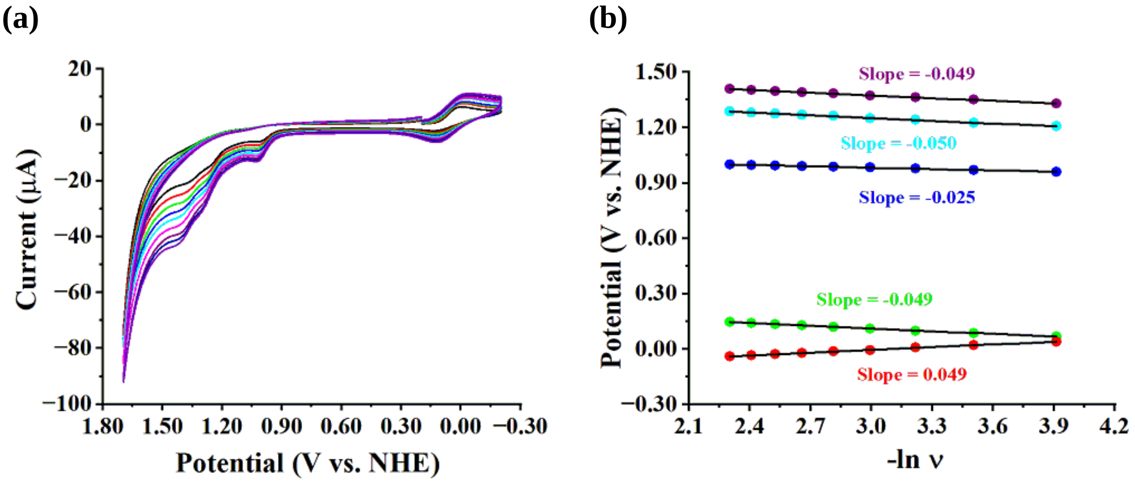

Fig. S11 **(a)** Cyclic voltamograms of complex **1** at 20 (black), 30 (red), 40 (green), 50 (blue), 60 (cyan), 70 (magenta), 80 (purple), 90 (royal) and 100 (violet) mVs^{-1} scan rates in 0.1 M neutral phosphate buffer. **(b)** Plot of –ln υ vs. potential of complex **1** for cathodic current of Cu(II)-Cu(I) couple (red dot), anodic current of Cu(II)-Cu(I) couple (green dot), 2nd anodic peak (blue dot), 3rd anodic peak (cyan dot) and 4th anodic peak (purple dot).

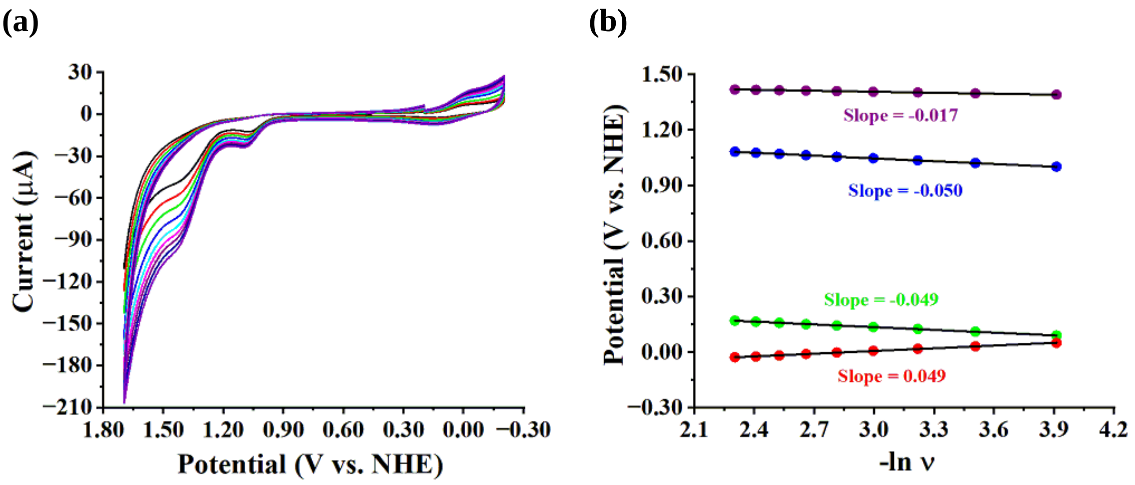

Fig. S12 **(a)** Cyclic voltamograms of complex **2** at 20 (black), 30 (red), 40 (green), 50 (blue), 60 (cyan), 70 (magenta), 80 (purple), 90 (royal) and 100 (violet) mVs^{-1} scan rates in 0.1 M neutral phosphate buffer. **(b)** Plot of –ln υ vs. potential of complex **2** for cathodic current of Cu(II)-Cu(I) couple (red dot), anodic current of Cu(II)-Cu(I) couple (green dot), 2nd anodic peak (blue dot) and 3rd anodic peak (cyan dot).

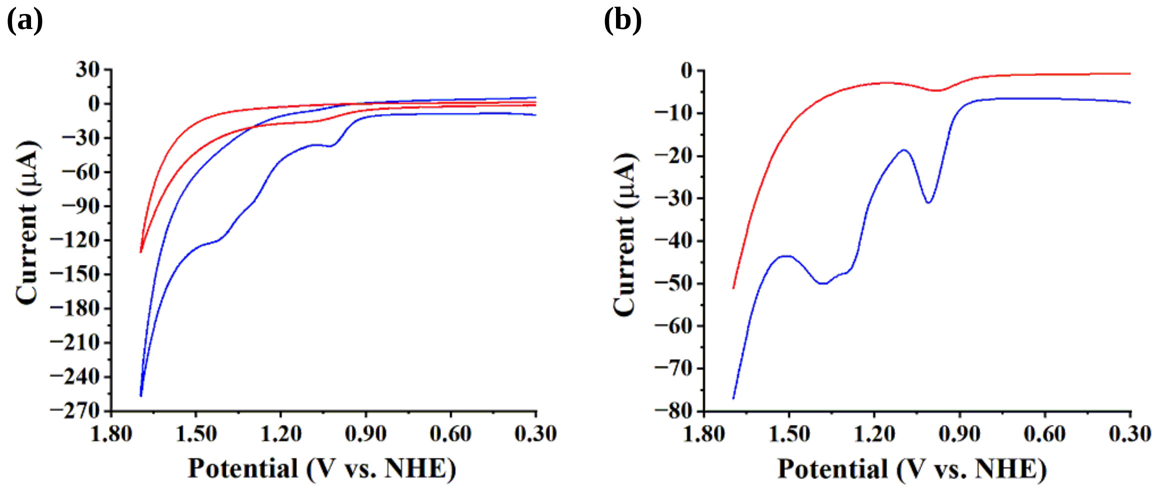

Fig. S13 **(a)** Cyclic voltammograms and **(b)** Differential Pulse voltammograms of complex **1** (blue), and analogous Zn complex, **$[Zn(L_1H)(L_2)](ClO_4)_2$** (red) in 0.1 M neutral phosphate buffer recorded with a glassy carbon (GC) working electrode, a Ag/AgCl reference electrode and a Pt counter electrode, scan rate 100 mVs^{-1}.

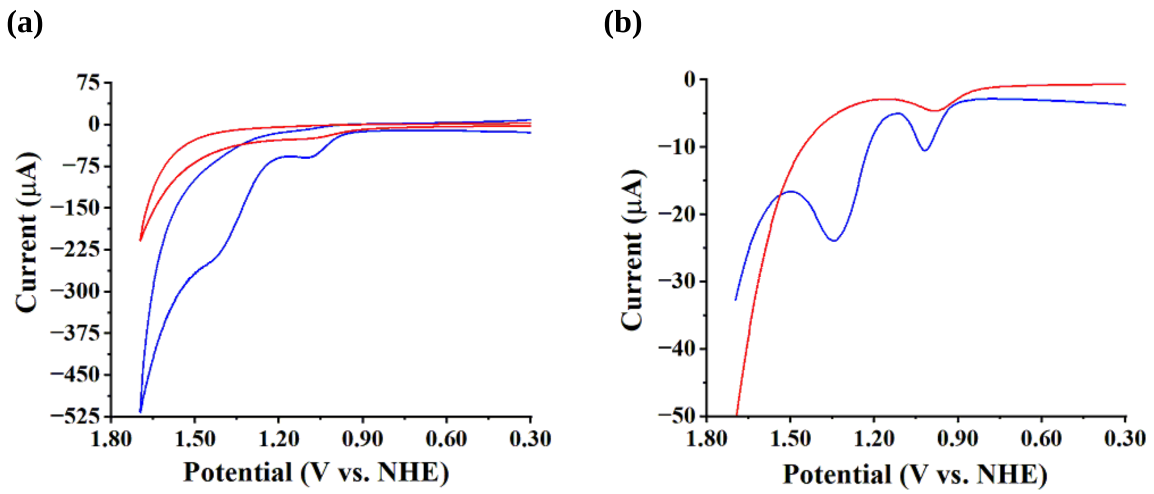

Fig. S14 **(a)** Cyclic voltammograms and **(b)** Differential Pulse voltammograms of complex **2** (blue), and analogous Zn complex, **$[Zn(L_1H)(L_3)](ClO_4)_2$** (red) in 0.1 M neutral phosphate buffer recorded with a glassy carbon (GC) working electrode, a Ag/AgCl reference electrode and a Pt counter electrode, scan rate 100 mVs^{-1}.

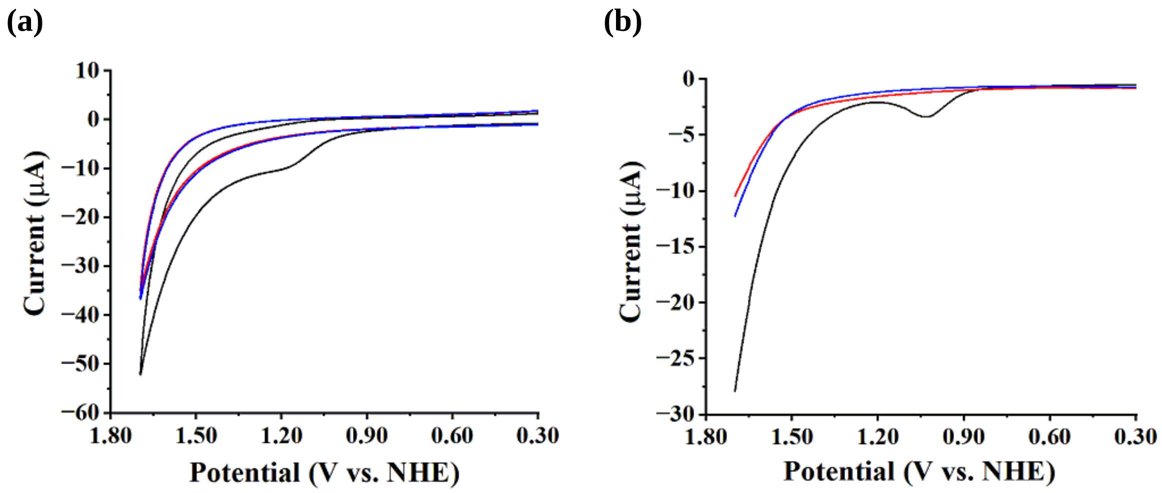

Fig. S15 **(a)** Cyclic voltammograms and **(b)** Differential Pulse voltammograms of ligand **L_1H**, N^1-(2-aminoethyl)ethane-1,2-diamine (black), ligand **L_2**, Pyridin-2-ylmethanamine (red) and ligand **L_3**, 2-(Pyridin-2-yl)ethan-1-amine (blue) in 0.1 M neutral phosphate buffer recorded with a glassy carbon (GC) working electrode, a Ag/AgCl reference electrode and a Pt counter electrode, scan rate 100 mVs^{-1}.

(a) **(b)**

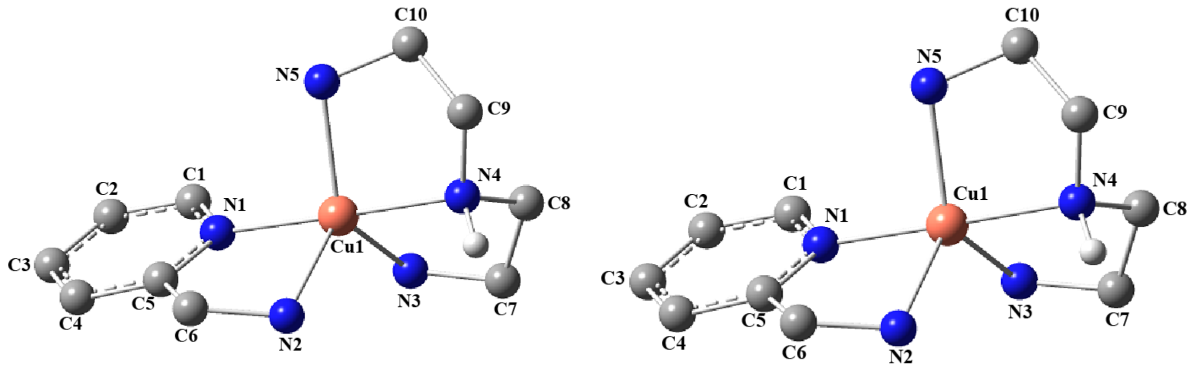

Spin Density at
Cu1: 0.63755, N1: 0.10279, N4: 0.14484

Spin Density at
Cu1: 0.58343, N1: 0.36709, C1: 0.46504,
C2: 0.24428, C3: 0.37665, C4: 0.30147,
C5: 0.36709

Fig. S16 DFT optimized structure of **(a)** complex **1** in doublet state, **(b)** complex **1** in quartet state (unimportant hydrogen atoms are not shown for clarity).

(a) **(b)**

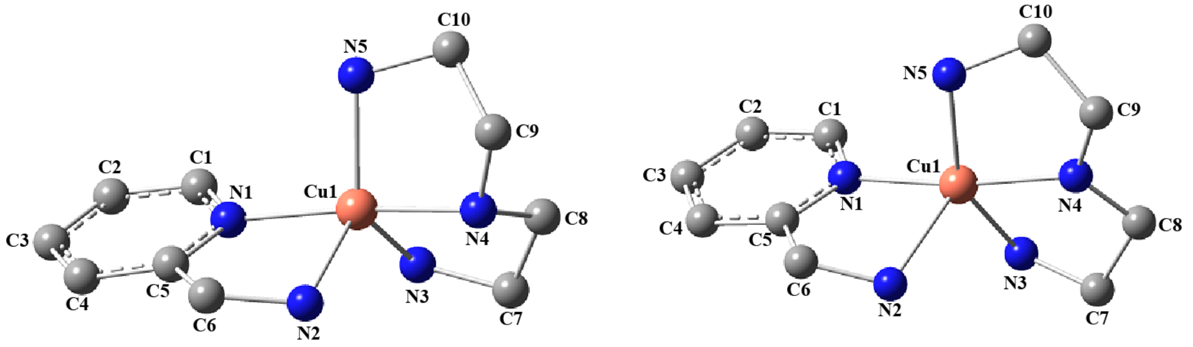

Spin Density at
Cu1: 0.57358, N1: 0.08989, N3: 0.10225,
N4: 0.98666, N5: 0.10803

Fig. S17 DFT optimized structure of **(a)** complex **1a** in singlet state, **(b)** complex **1a** in triplet state (unimportant hydrogen atoms are not shown for clarity).

(a) (b)

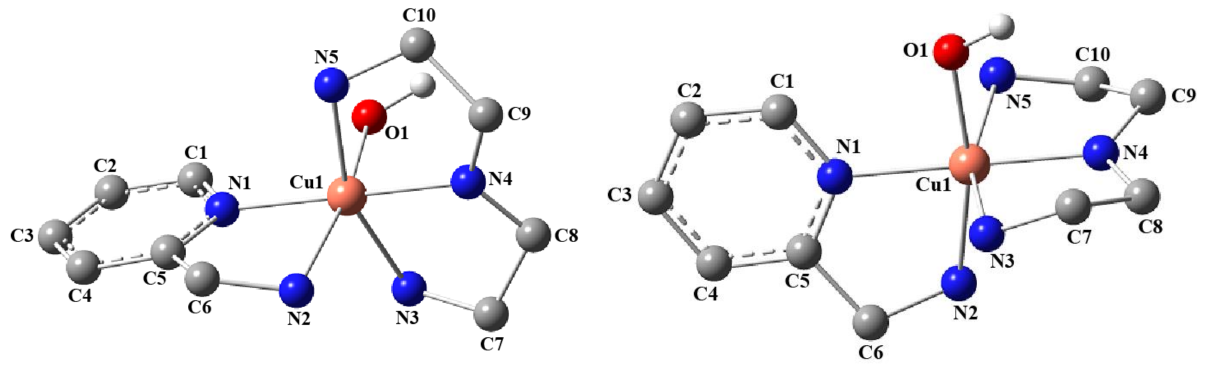

Spin Density at
Cu1: 0.55004, N1: 0.11902, N3: 0.14404,
N4: 0.15025, N5: 0.13770

Spin Density at
Cu1: 0.63505, N1: 0.12443, N2: 0.12860,
N3: 0.19237, N4: 1.05784, N5: 0.22120,
O1: 0.50326

Fig. S18 DFT optimized structure of **(a)** complex **1b** in doublet state, **(b)** complex **1b** in quartet state (unimportant hydrogen atoms are not shown for clarity).

(a) (b)

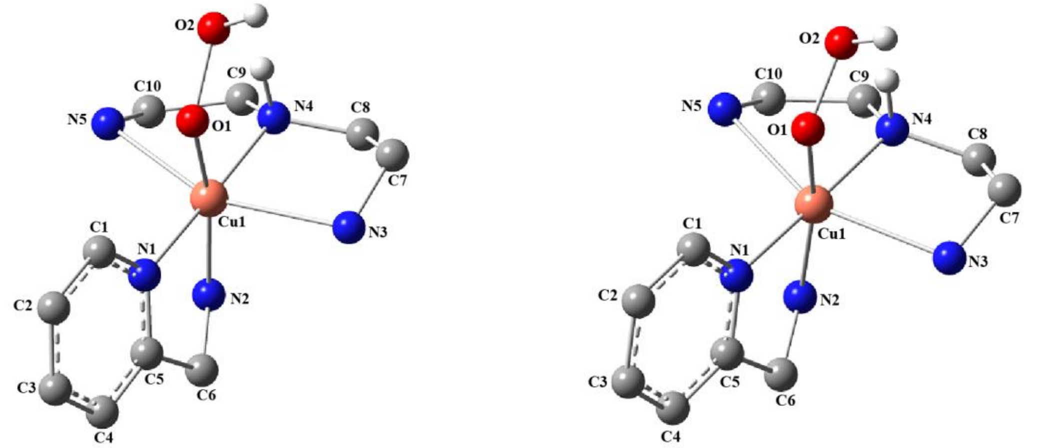

Spin Density at
Cu1: 0.59966, N1: 0.11809, N2: 0.08926,
N3: 0.18213, N4: 0.20295, N5: 0.12403,
O1: 0.48153, O2: 0.18335

Fig. S19 DFT optimized structure of Transition State (**TS**) of complex **1** **(a)** in singlet state, **(b)** in triplet state (unimportant hydrogen atoms are not shown for clarity).

(a) **(b)**

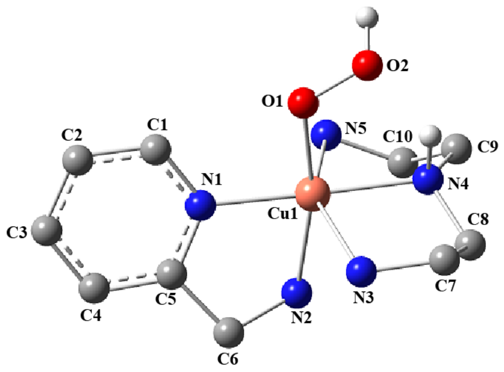

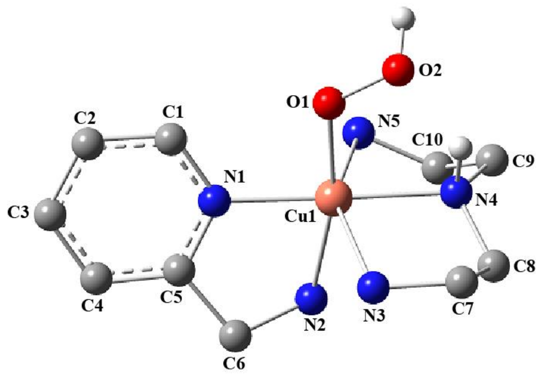

Spin Density at
Cu1: 0.60449, N1: 0.12214, N2: 0.08247,
N3: 0.11461, N4: 0.19570, N5: 0.18530,
O1: 0.48535, O2: 0.19579

Fig. S20 DFT optimized structure of **(a)** complex **1c** in singlet state, **(b)** complex **1c** in triplet state (unimportant hydrogen atoms are not shown for clarity).

(a) **(b)**

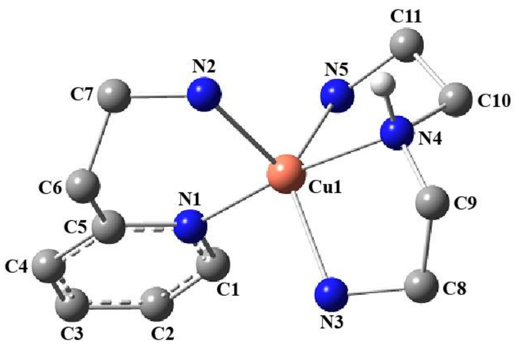

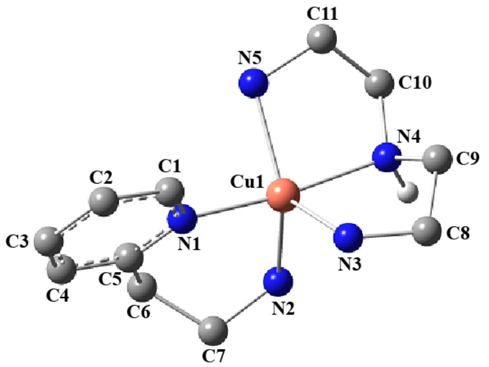

Spin Density at
Cu1: 0.57526, N1: 0.10382, N2: 0.04656,
N3: 0.07575, N4: 0.14684

Spin Density at
Cu1: 0.57848, N1: 0.37476, N2: 0.05117,
N4: 0.13177, C1: 0.43941, C2: 0.25384,
C3: 0.37424, C4: 0.29316, C5: 0.40294

Fig. S21 DFT optimized structure of **(a)** complex **2** in doublet state, **(b)** complex **2** in quartet state (unimportant hydrogen atoms are not shown for clarity).

(a) (b)

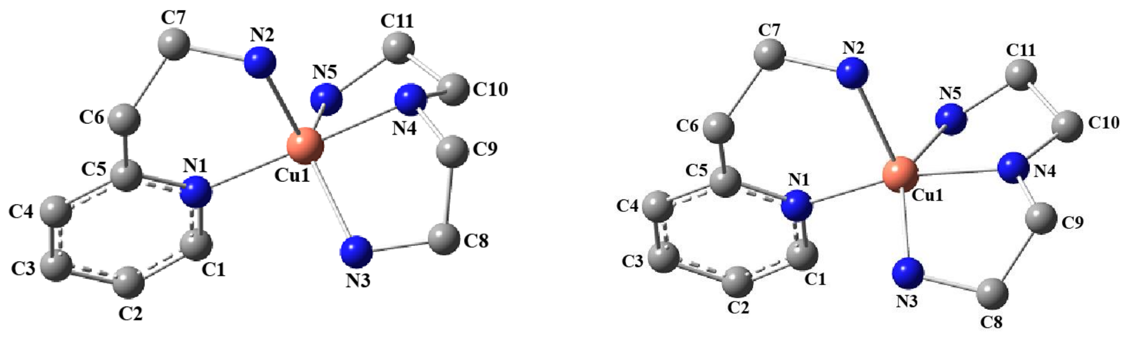

Spin Density at
Cu1: 0.57026, N1: 0.09440, N2: 0.02981,
N3: 0.09357, N4: 0.99275, N5: 0.09605

Fig. S22 DFT optimized structure of **(a)** complex **2a** in singlet state, **(b)** complex **2a** in triplet state (unimportant hydrogen atoms are not shown for clarity).

(a) (b)

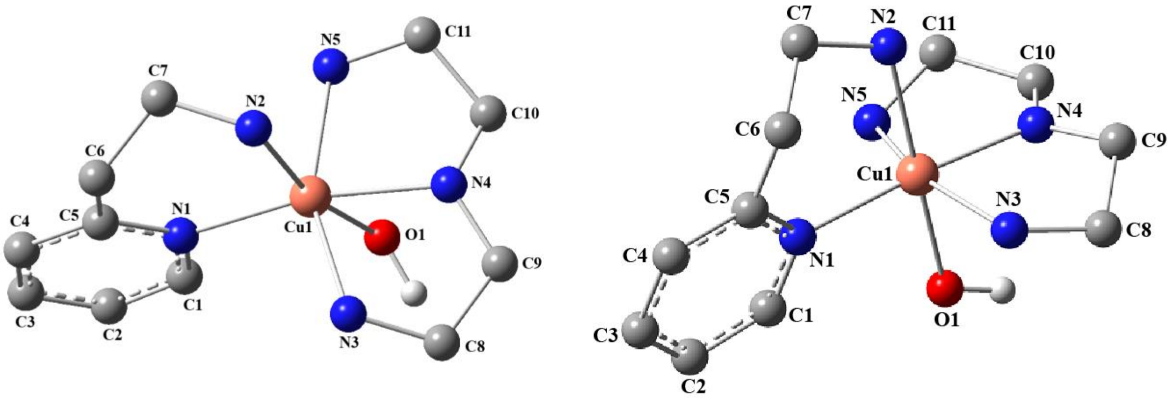

Spin Density at
Cu1: 0.295341, N3: 0.09631, N4: 0.86422,
N5: 0.10726, O1: 0.33469

Spin Density at
Cu1: 0.59421, N1: 0.15373, N2: 0.10329,
N3: 0.17631, N4: 1.26722, N5: 0.10716,
O1: 1.37479

Fig. S23 DFT optimized structure of **(a)** complex **2b** in doublet state, **(b)** complex **2b** in quartet state (unimportant hydrogen atoms are not shown for clarity).

(a) **(b)**

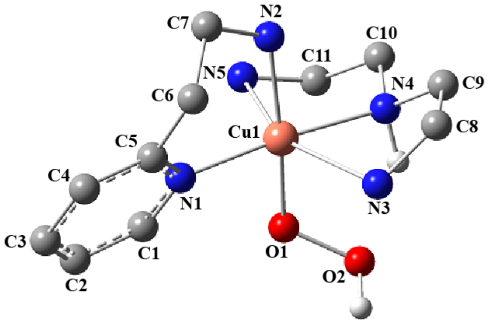

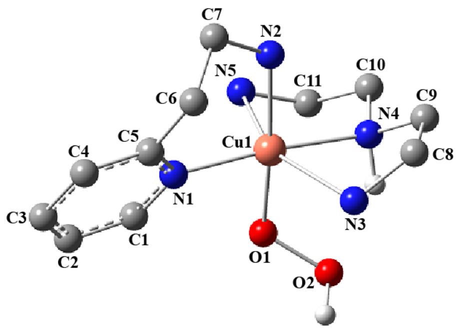

Spin Density at
Cu1: 0.58716, N1: 0.11366, N2: 0.09495,
N3: 0.10299, N4: 0.22487, N5: 0.17794,
O1: 0.48542, O2: 0.18941

Fig. S24 DFT optimized structure of Transition State (**TS**) of complex **2** **(a)** in singlet state, **(b)** in triplet state (unimportant hydrogen atoms are not shown for clarity).

(a) **(b)**

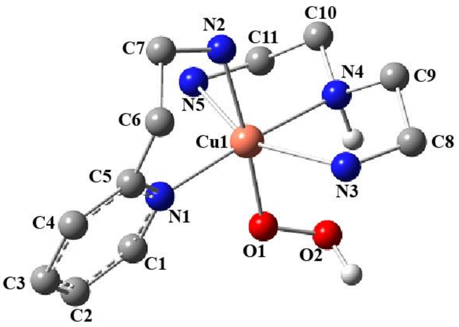

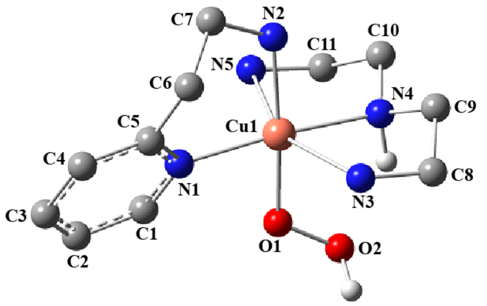

Spin Density at
Cu1: 0.58980, N1: 0.11172, N2: 0.09700,
N3: 0.17213, N4: 0.21380, N5: 0.09061,
O1: 0.50152, O2: 0.20133

Fig. S25 DFT optimized structure of **(a)** complex **2c** in singlet state, **(b)** complex **2c** in triplet state (unimportant hydrogen atoms are not shown for clarity).

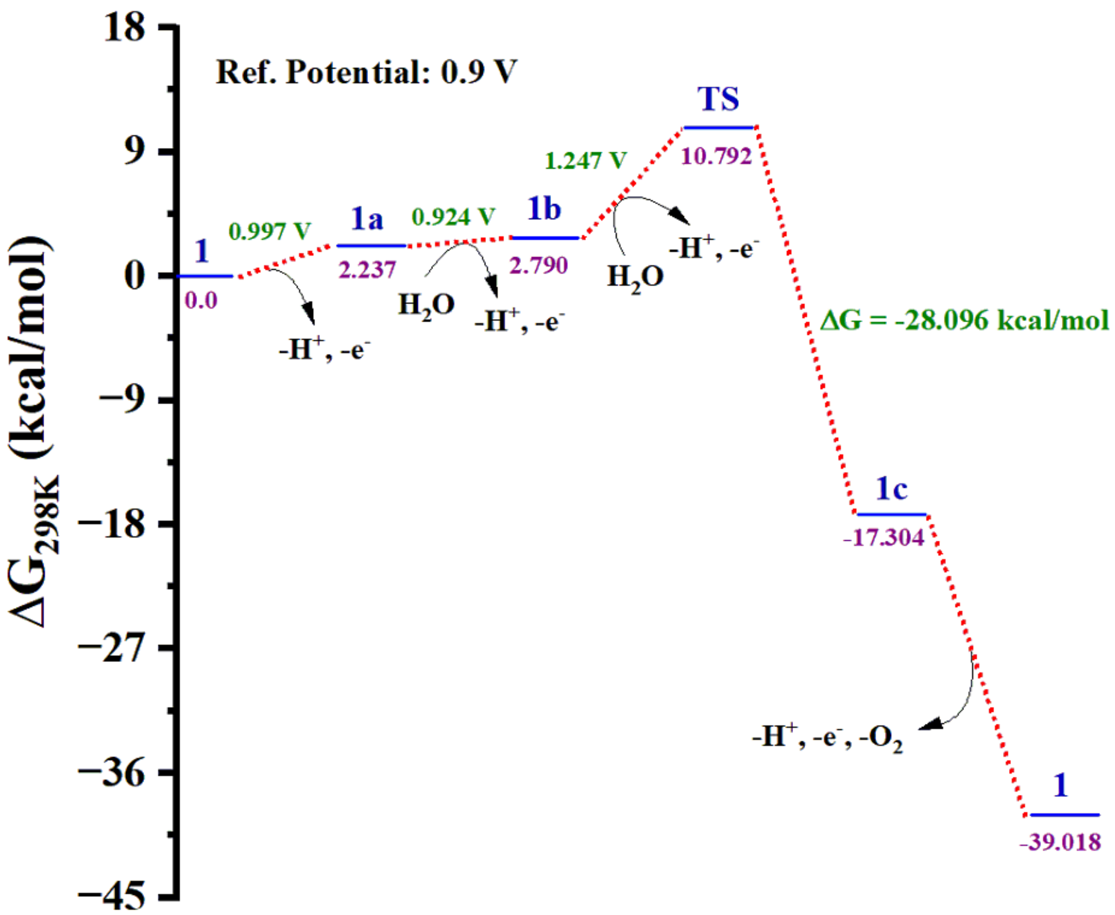

Fig. S26 Energy diagram (ΔG_{298K} in kcal/mol) for water oxidation catalysed by complex **1**. The reference potential of 0.9 V is used to set up the thermodynamics. To construct the energy diagram for the full catalytic cycle, the total exergonicity of 39.018 kcalmol^{-1} derived from the experimental over-potential (onset potential of 1.24 V, over-potential 0.423 V at pH 7) was used.

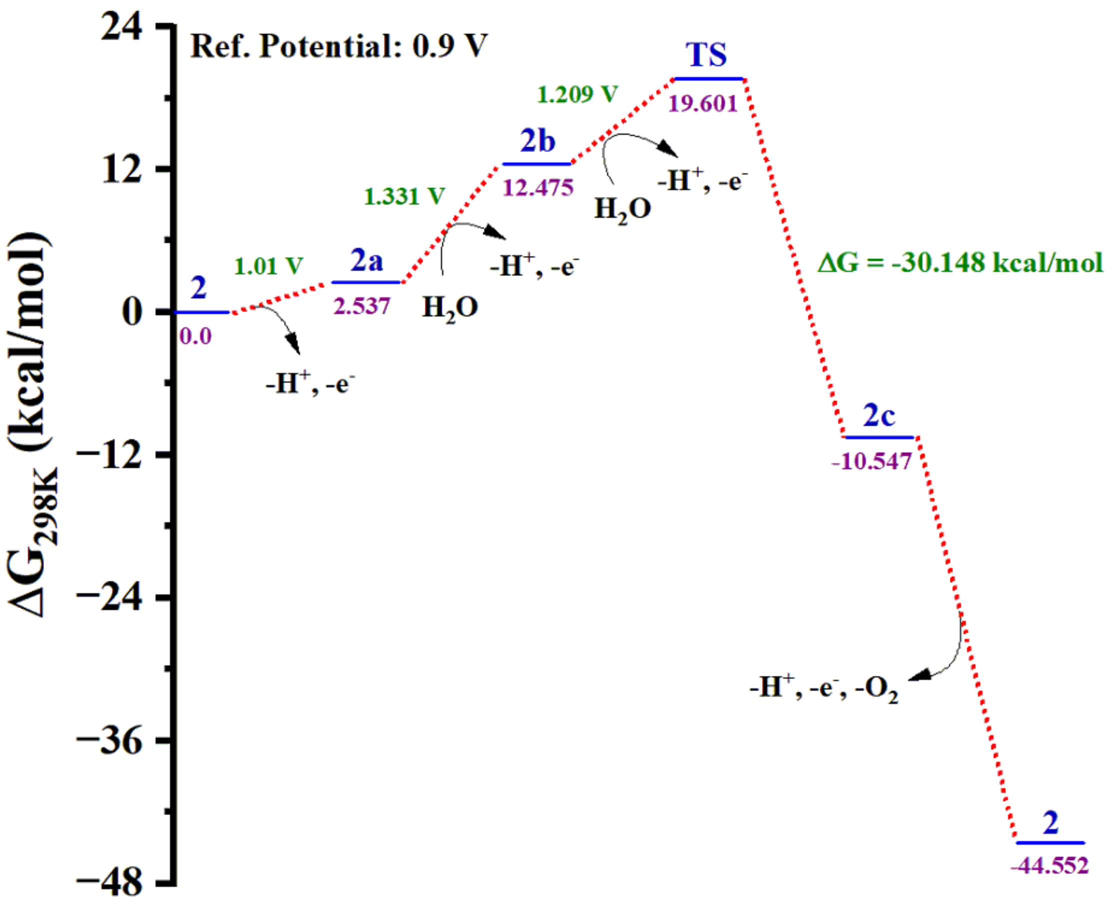

Fig. S27 Energy diagram (ΔG_{298K} in kcal/mol) for water oxidation catalysed by complex **2**. The reference potential of 0.9 V is used to set up the thermodynamics. To construct the energy diagram for the full catalytic cycle, the total exergonicity of 44.552 kcalmol^{-1} derived from the experimental over-potential (onsite potential of 1.30 V, over-potential 0.483 V at pH 7) was used.

(a)

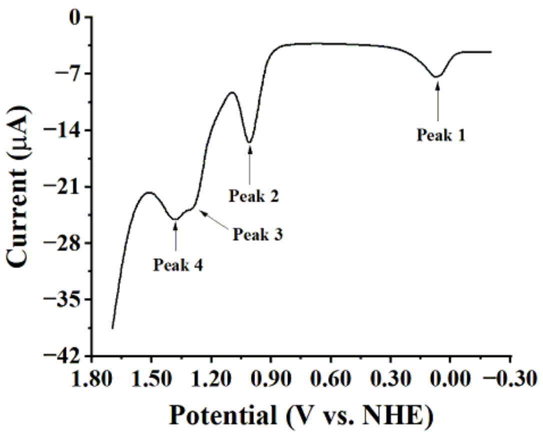

(b)

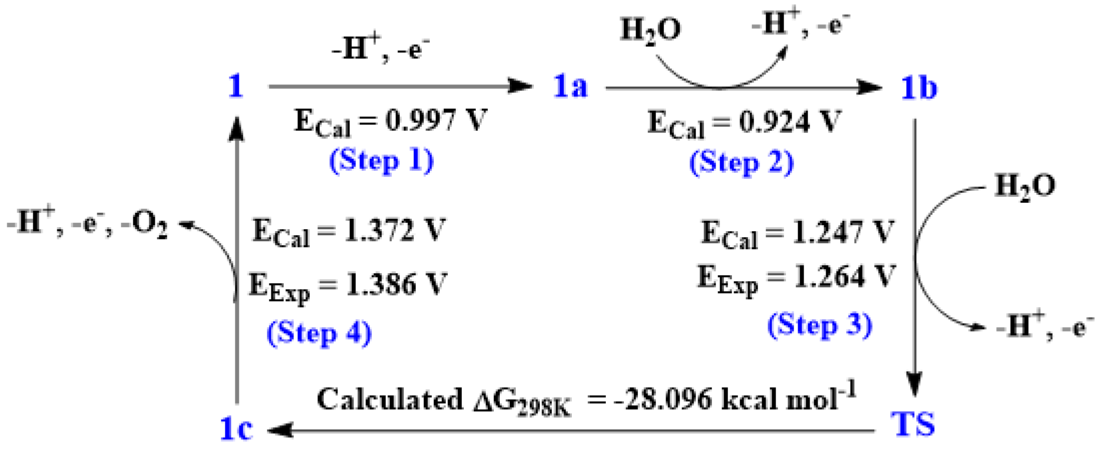

(c)

	Experimental peak position (V vs. NHE) and Assignment	Theoretically calculated redox potential (V vs. NHE) and assignment
**Peak 2**	**1.009 V**<br>Complex **1** ⟶ Complex **1b** + $2H^+$ + $2e^-$	**0.997 V**<br>Complex **1** ⟶ Complex **1a** + H^+ + e^-<br>**0.924 V**<br>Complex **1a** + H_2O ⟶ Complex **1b** + H^+ + e^-
**Peak 3**	**1.264 V**<br>Complex **1b** + H_2O ⟶ **TS** + H^+ + e^-	**1.247 V**<br>Complex **1b** + H_2O ⟶ **TS** + H^+ + e^-
**Peak 4**	**1.386 V**<br>Complex **1c** ⟶ Complex **1** + H^+ + e^- + O_2	**1.372 V**<br>Complex **1c** ⟶ Complex **1** + H^+ + e^- + O_2

Fig. S28 **(a)** Differential pulse voltammogram (DPV) of complex **1** in 0.1 M neutral phosphate buffer, **(b)** Proposed catalytic cycle of complex **1**, **(c)** Assignment of experimental and theoretical redox potentials to the proposed electrochemical steps in the catalytic cycle of complex **1**.

(a)

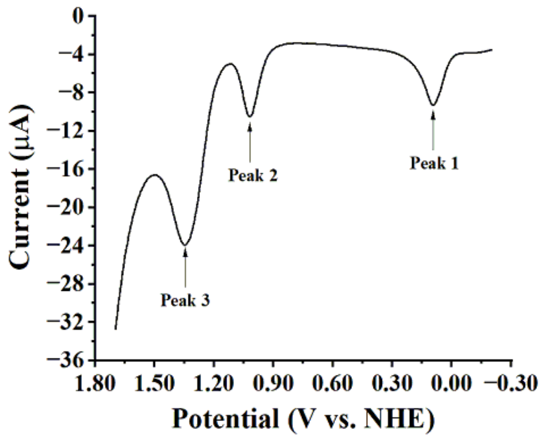

(b)

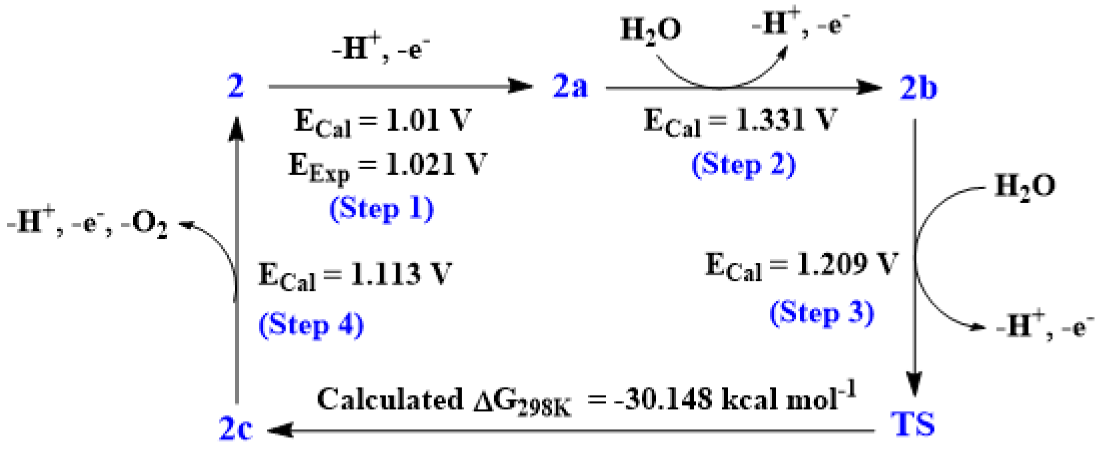

(c)

	**Experimental peak position (V vs. NHE) and Assignment**	**Theoretically calculated redox potential (V vs. NHE) and assignment**
**Peak 2**	**1.021 V** Complex **2** ⟶ Complex **2a** + H^+ + e^-	**1.01 V** Complex **2** ⟶ Complex **2a** + H^+ + e^-
**Peak 3**	**1.349 V** Complex **2a** ⟶ Complex **2** + $3H^+$ + $3e^-$ + O_2	**1.331 V** Complex **2a** + H_2O ⟶ Complex **2b** + H^+ + e^-
		**1.209 V** Complex **2b** + H_2O ⟶ **TS** + H^+ + e^-
		**1.113 V** Complex **2c** ⟶ Complex **2** + H^+ + e^- + O_2

Fig. S29 **(a)** Differential pulse voltammogram (DPV) of complex **2** in 0.1 M neutral phosphate buffer, **(b)** Proposed catalytic cycle of complex **2**, **(c)** Assignment of experimental and theoretical redox potentials to the proposed electrochemical steps in the catalytic cycle of complex **2**.

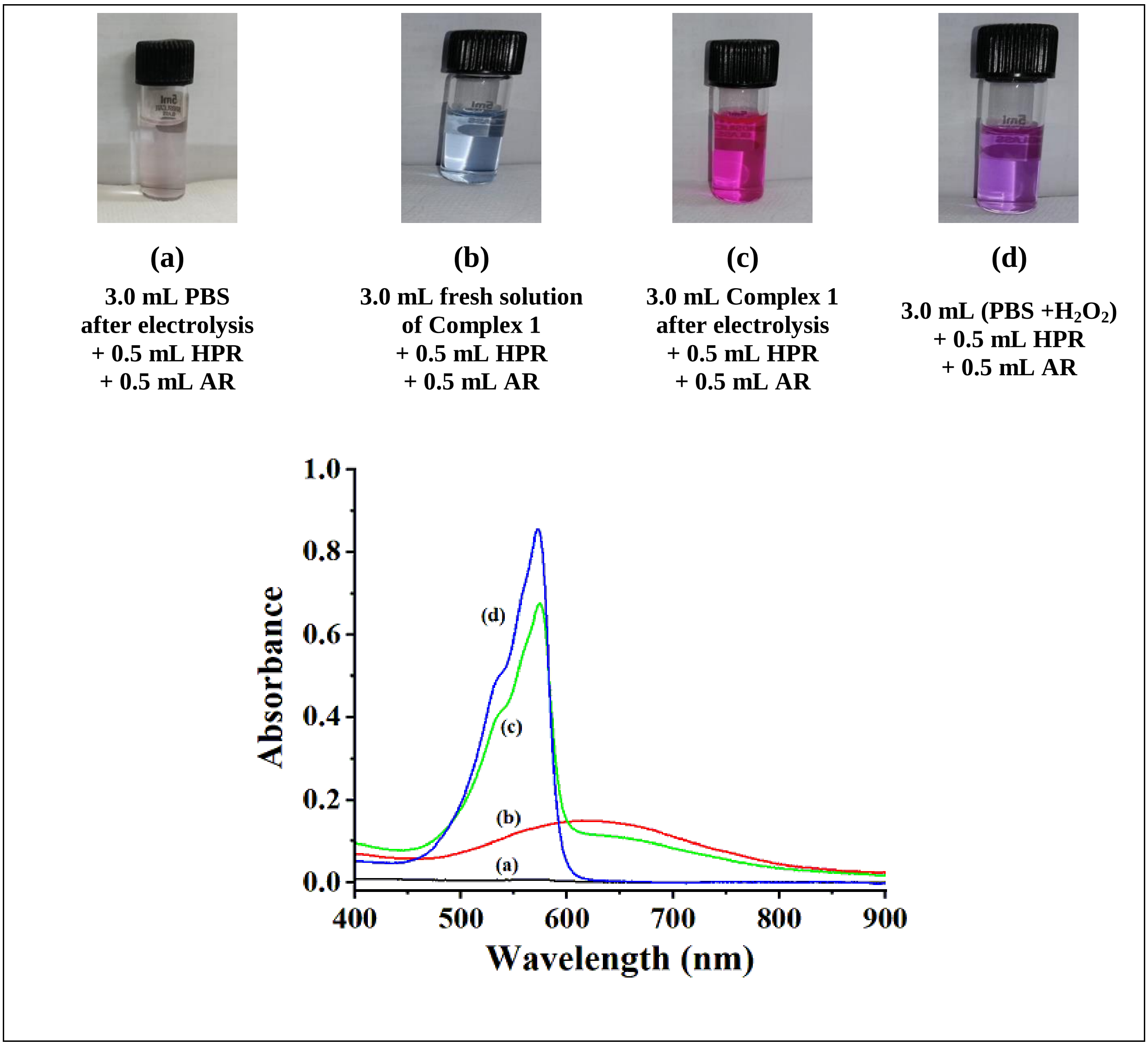

Fig. S30 UV-visible spectra obtained after **(a)** addition of HPR and AR on buffer solution after electrolysis, **(b)** addition of HPR and AR on fresh solution of complex **1**, **(c)** addition of HPR and AR on solution of complex **1** after electrolysis, **(d)** addition of HPR and AR on buffer solution containing 100 µL of H_2O_2.

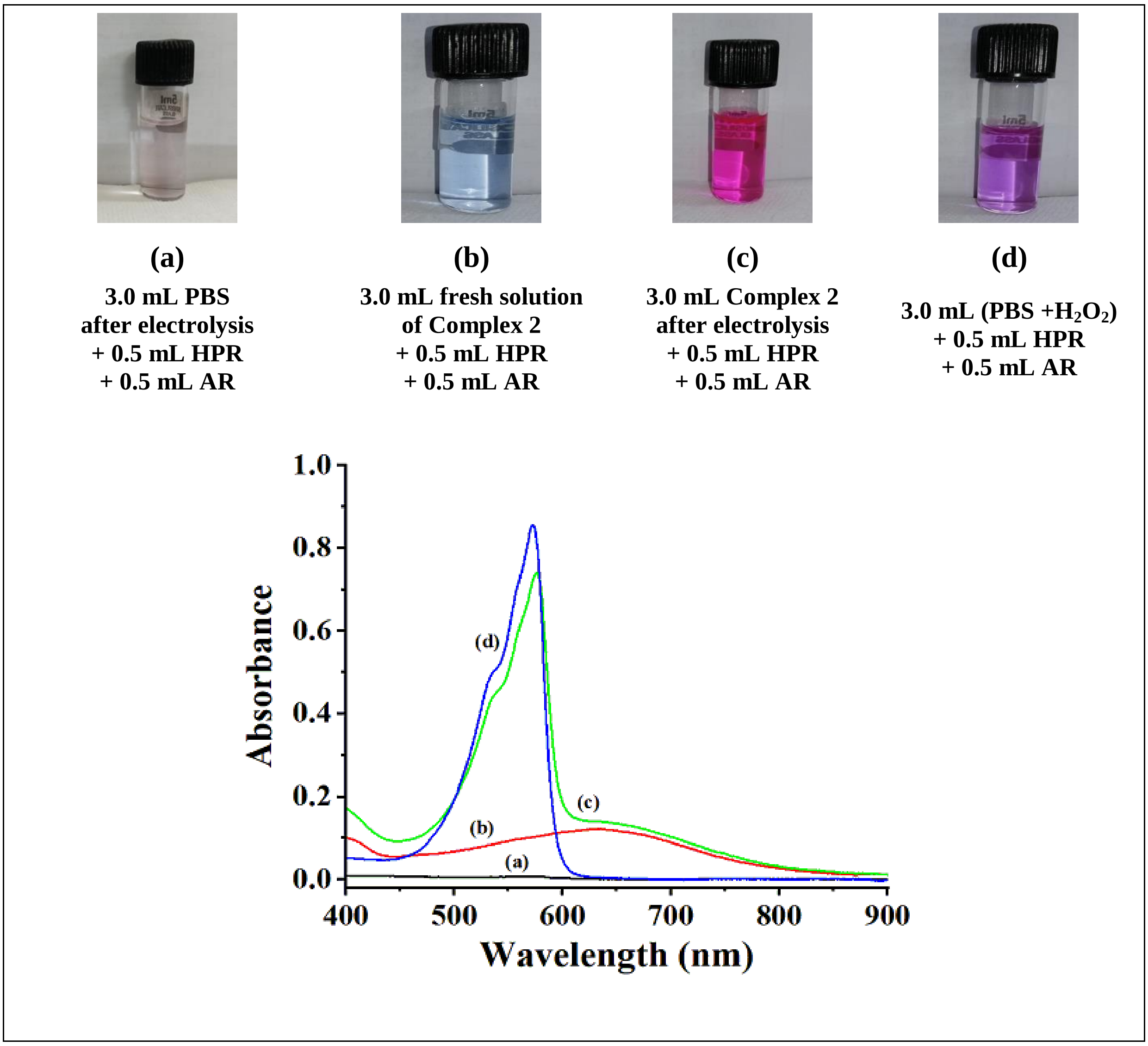

Fig. S31 UV-visible spectra obtained after **(a)** addition of HPR and AR on buffer solution after electrolysis, **(b)** addition of HPR and AR on fresh solution of complex **2**, **(c)** addition of HPR and AR on solution of complex **2** after electrolysis, **(d)** addition of HPR and AR on buffer solution containing 100 μL of H_2O_2.

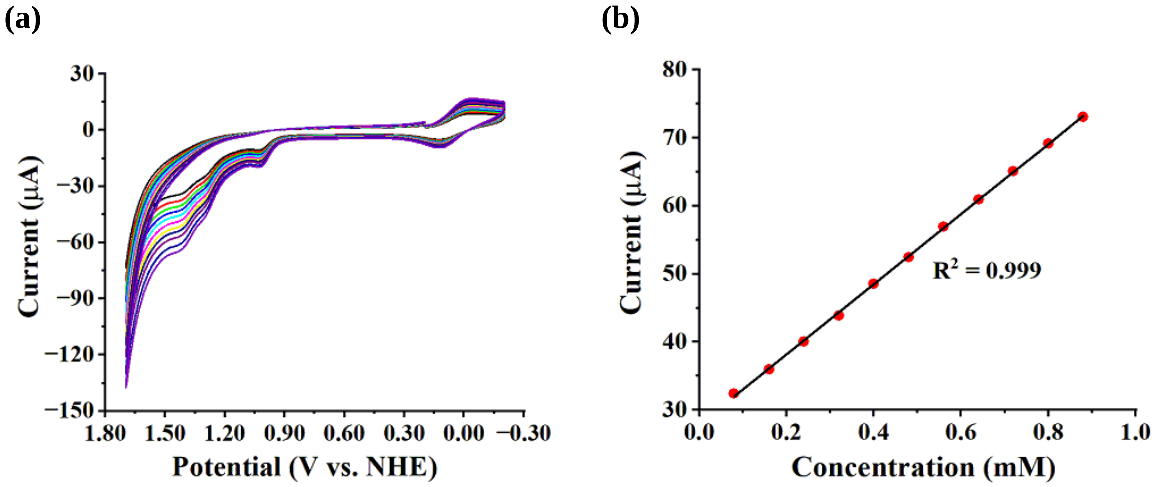

Fig. S32 **(a)** Cyclic voltammograms of complex **1** at 0.08 (black), 0.16 (red), 0.24 (green), 0.32 (blue), 0.40 (cyan), 0.48 (magenta), 0.56 (yellow), 0.64 (navy), 0.72 (purple), 0.80 (royal) and 0.88 mM (violet) concentration in 0.1 M neutral phosphate buffer. **(b)** Catalytic current at 1.38 V vs. NHE for complex **1** as a function of the catalyst concentration from 0.08 mM to 0.88 mM in 0.1 M neutral phosphate buffer.

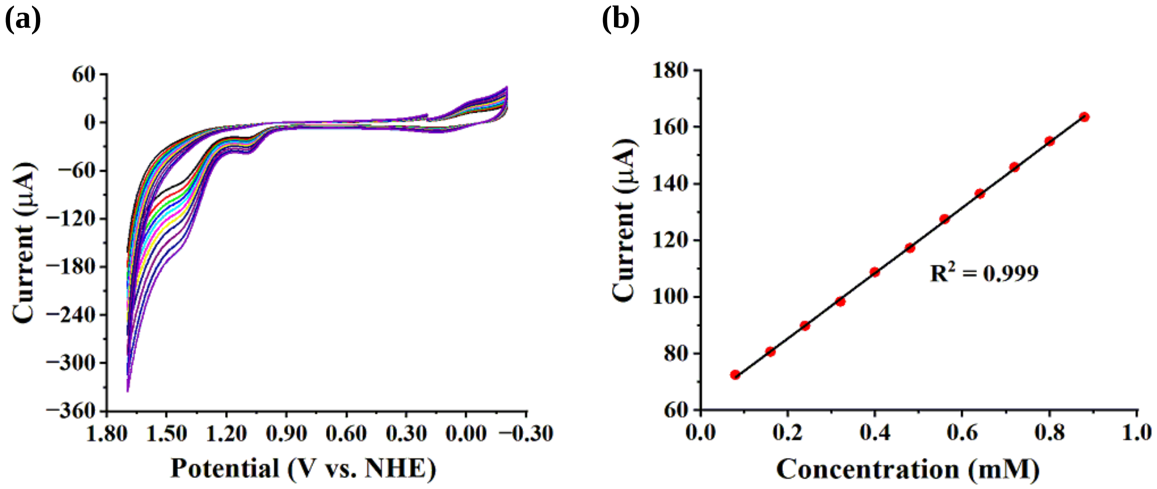

Fig. S33 **(a)** Cyclic voltammograms of complex **2** at 0.08 (black), 0.16 (red), 0.24 (green), 0.32 (blue), 0.40 (cyan), 0.48 (magenta), 0.56 (yellow), 0.64 (navy), 0.72 (purple), 0.80 (royal) and 0.88 mM (violet) concentration in 0.1 M neutral phosphate buffer. **(b)** Catalytic current at 1.35 V vs. NHE for complex **2** as a function of the catalyst concentration from 0.08 mM to 0.88 mM in 0.1 M neutral phosphate buffer.

(a) (b)

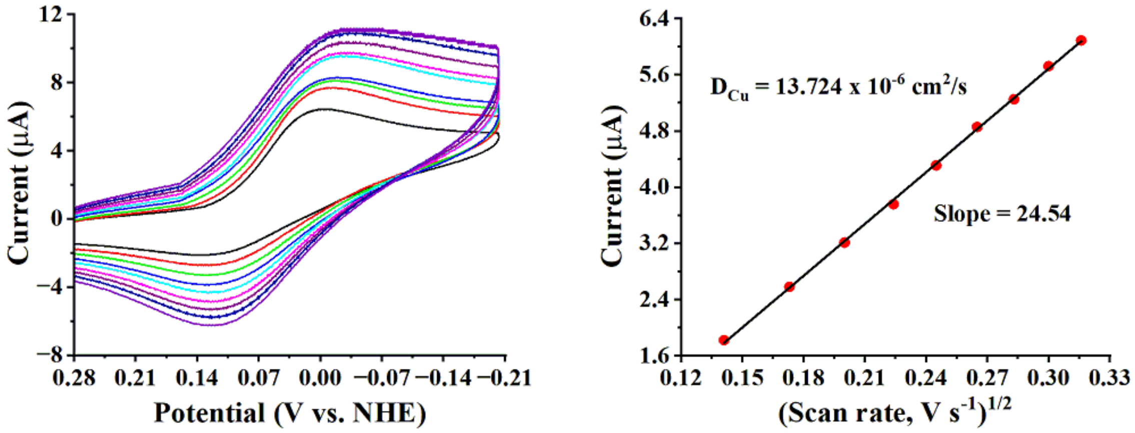

Fig. S34 **(a)** Cyclic voltammograms of complex **1** in the range 0.28 to -0.21 V vs. NHE at 20 (black), 30 (red), 40 (green), 50 (blue), 60 (cyan), 70 (magenta), 80 (purple), 90 (royal) and 100 (violet) mVs^{-1} scan rate. **(b)** Dependence of the peak current for the Cu^{II}/Cu^{I} couple of complex **1** on the square root of scan rate with standard three electrode system in 0.1 M neutral phosphate buffer. D_{Cu} = 13.724 x 10^{-6} cm^2/s.

(a) (b)

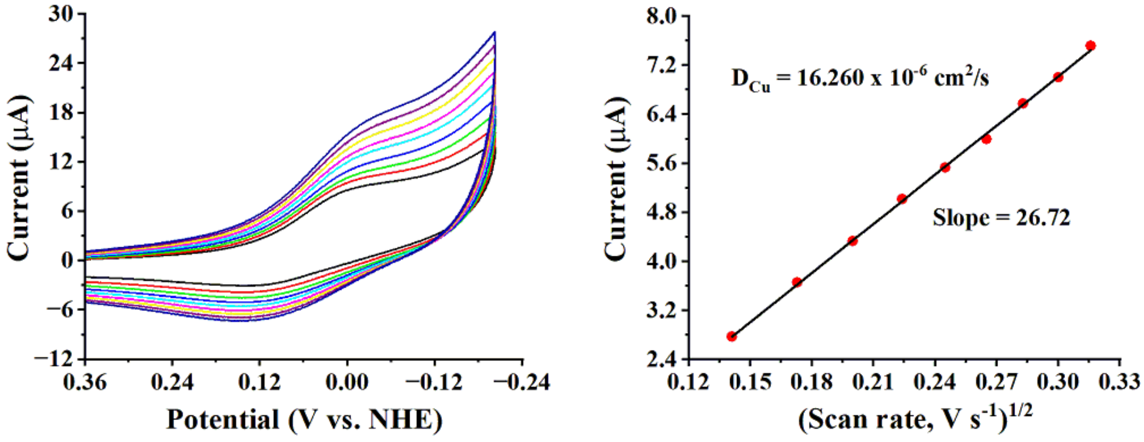

Fig. S35 **(a)** Cyclic voltammograms of complex **2** in the range 0.36 to -0.24 V vs. NHE at 20 (black), 30 (red), 40 (green), 50 (blue), 60 (cyan), 70 (magenta), 80 (purple), 90 (royal) and 100 (violet) mVs^{-1} scan rate. **(b)** Dependence of the peak current for the Cu^{II}/Cu^{I} couple of complex **2** on the square root of scan rate with standard three electrode system in 0.1 M neutral phosphate buffer. D_{Cu} = 16.260 x 10^{-6} cm^2/s.

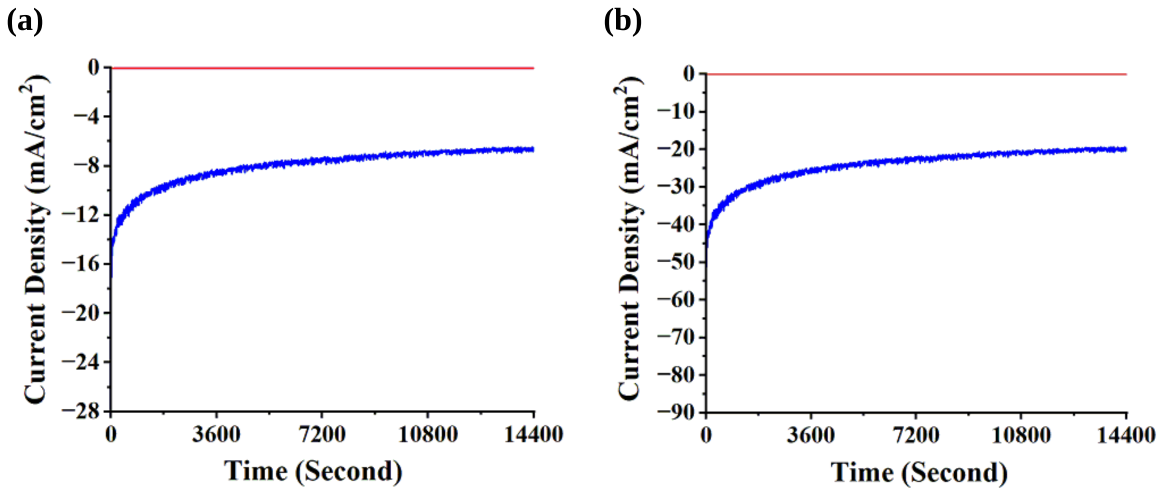

Fig. S36 Plot of current density vs. time recorded during 4 hour of bulk electrolysis with (blue line) and without (red line) **(a)** complex **1** and **(b)** complex **2** in 0.1 M neutral phosphate buffer using ITO working electrode (area 4 cm^2), Ag/AgCl reference electrode and Pt counter electrode at 1.38 V vs. NHE and 1.35 V vs. NHE respectively.

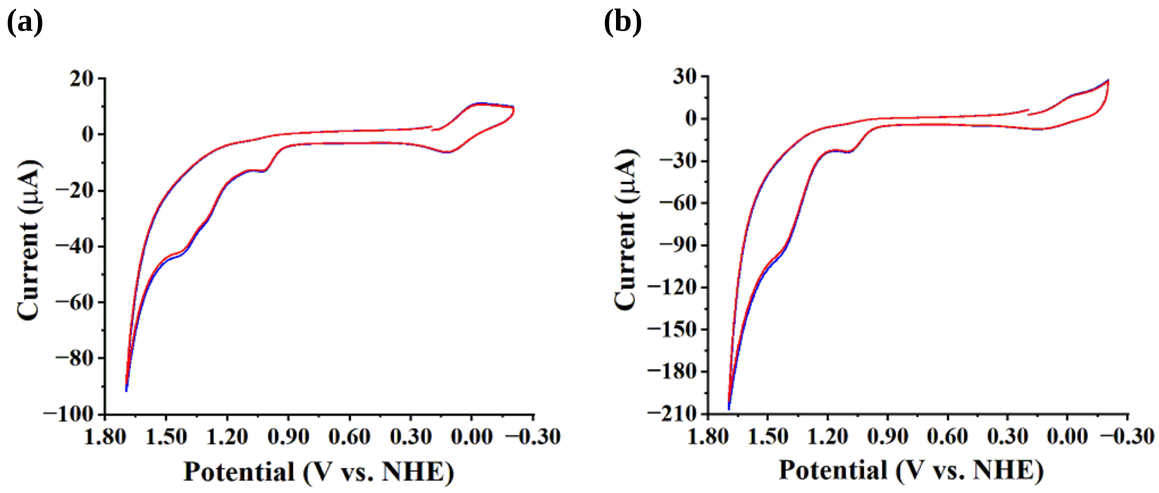

Fig. S37 Cyclic voltammograms of **(a)** complex **1** and **(b)** complex **2** recorded before (blue) and after (red) 4 hour of bulk electrolysis at 1.38 V and 1.35 V vs. NHE respectively in 0.1 M neutral phosphate buffer using glassy carbon (GC) as working electrode (area 0.07 cm^2), Ag/AgCl as reference electrode and Pt as counter electrode. Scan rate, 100 mVs^{-1}.

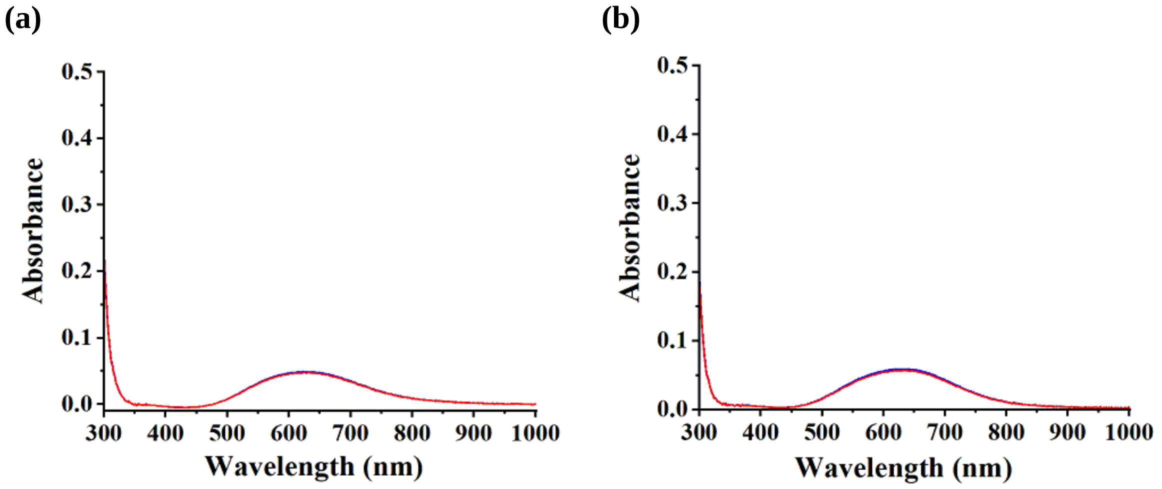

Fig. S38 UV-visible spectra of **(a)** complex **1** and **(b)** complex **2** recorded before (blue) and after (red) 4 hour of bulk electrolysis at 1.38 V and 1.35 V vs. NHE respectively in 0.1 M neutral phosphate buffer.

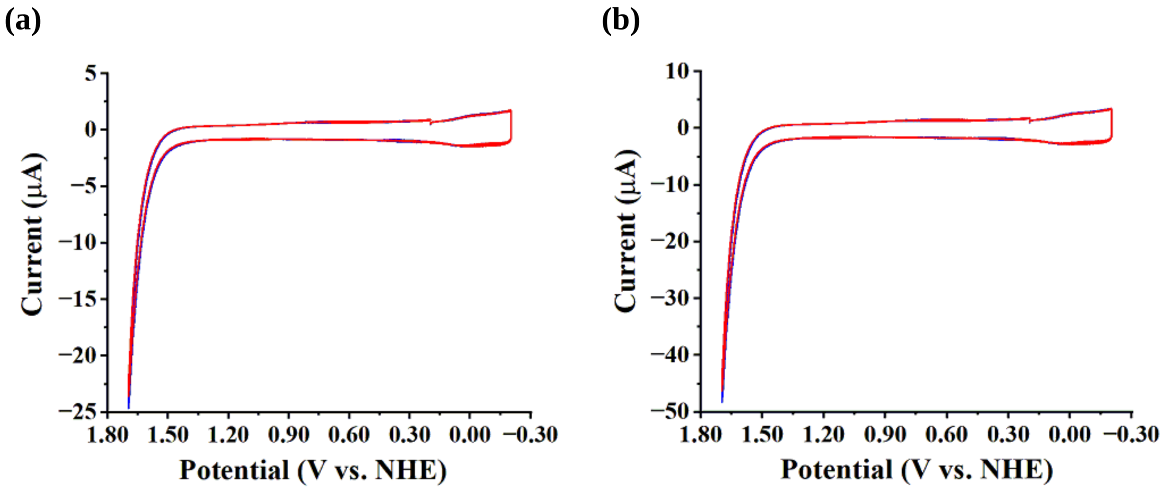

Fig. S39 Cyclic voltammograms recorded in 0.1 M neutral phosphate buffer in the absence of **(a)** complex **1** and **(b)** complex **2** with fresh (blue) and used (red) ITO working electrode (area 4 cm^2), a Ag/AgCl reference electrode and a Pt counter electrode, scan rate. 100 mVs^{-1}.

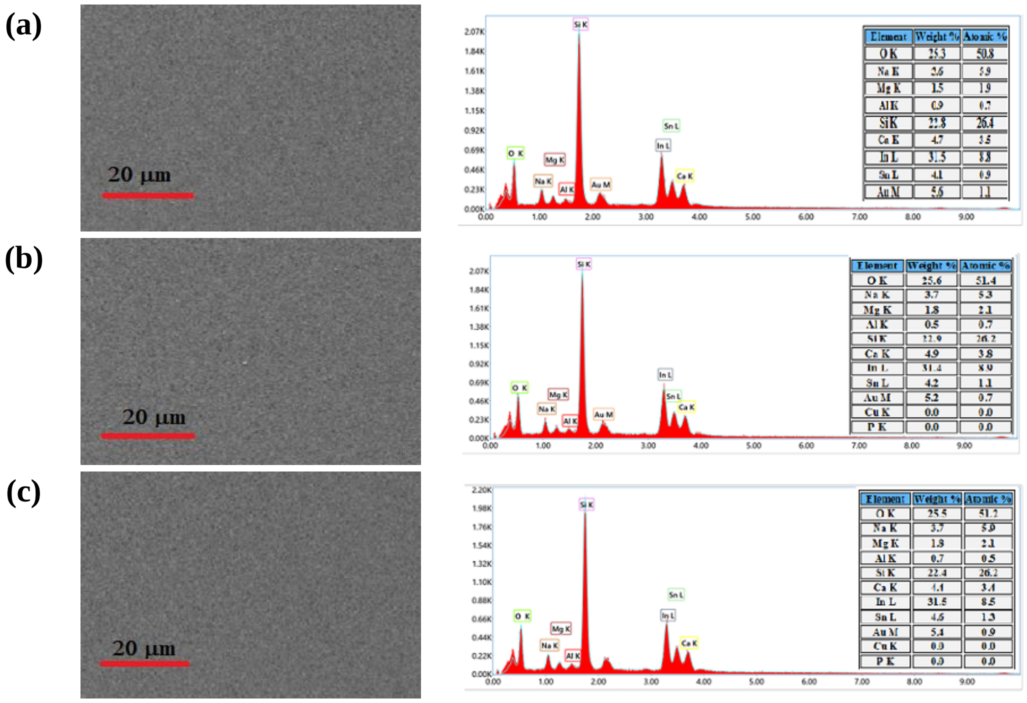

Fig. S40 FE-SEM and EDX plot of **(a)** fresh ITO working electrode **(b)** ITO working electrode after 4 hour of bulk electrolysis of complex **1** and **(c)** ITO working electrode after 4 hour of bulk electrolysis of complex **2** in 0.1 M neutral phosphate buffer.

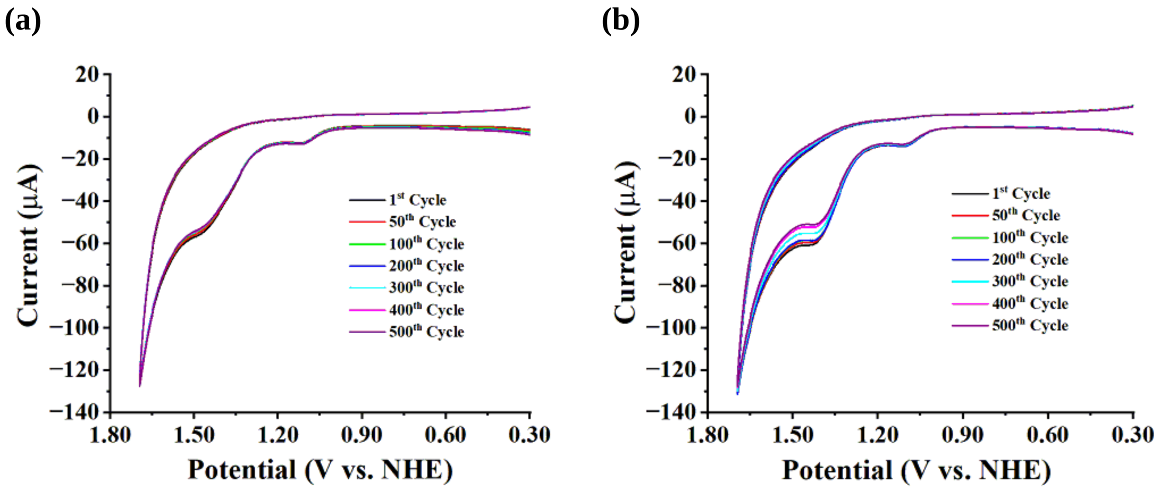

Fig. S41 Consecutive cyclic voltamograms of **(a)** complex **1** and **(b)** complex **2** recorded using glassy carbon working electrode (area 0.07 cm^2) at 100 mVs^{-1} scan rates in 0.1 M neutral phosphate buffer.

(a)

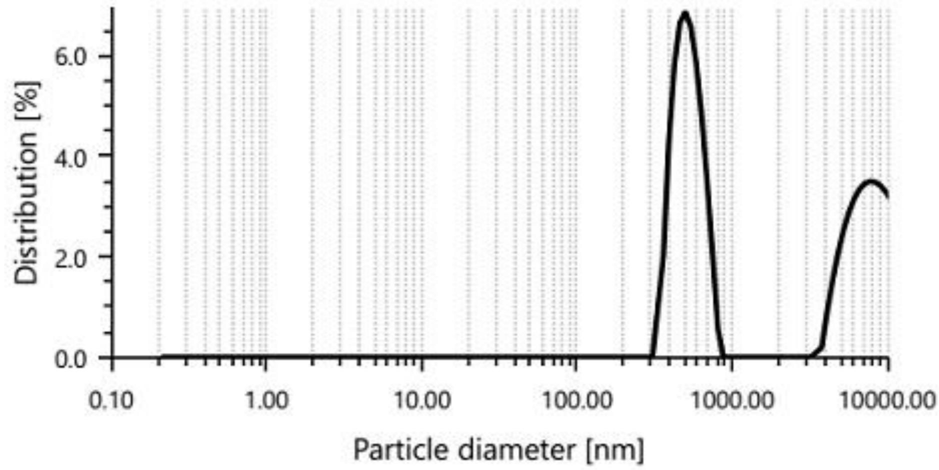

(b)

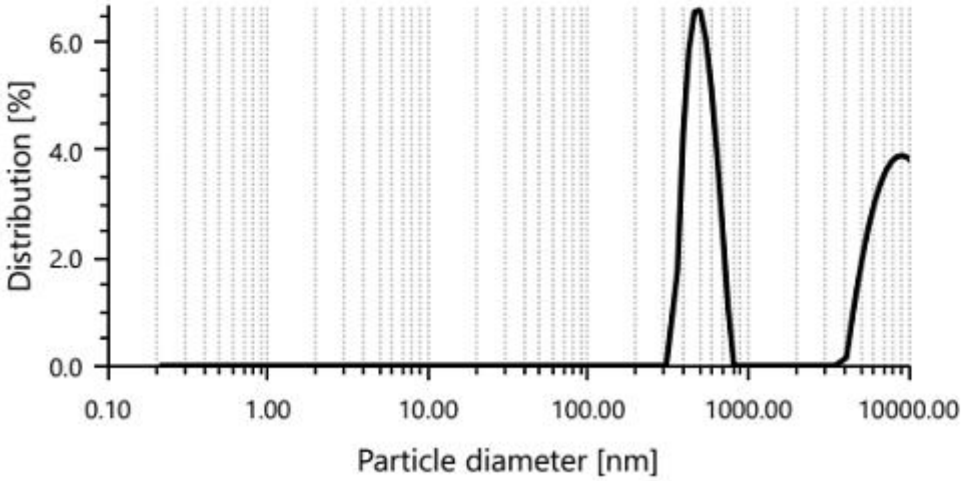

Fig. S42 Dynamic Light Scattering (DLS) size distribution of complex **1** measured in 0.1 M neutral phosphate buffer **(a)** before and **(b)** after 500 consecutive cycle cyclic voltammetry experiment.

(a)

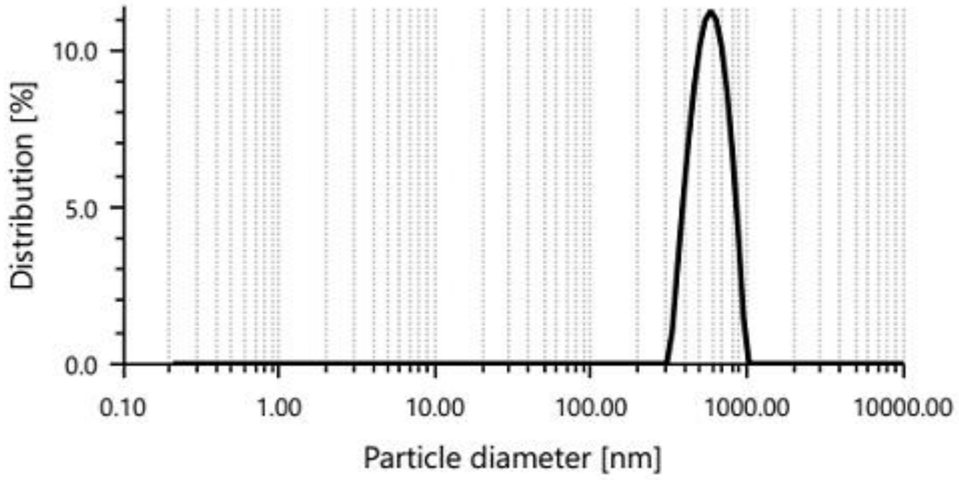

(b)

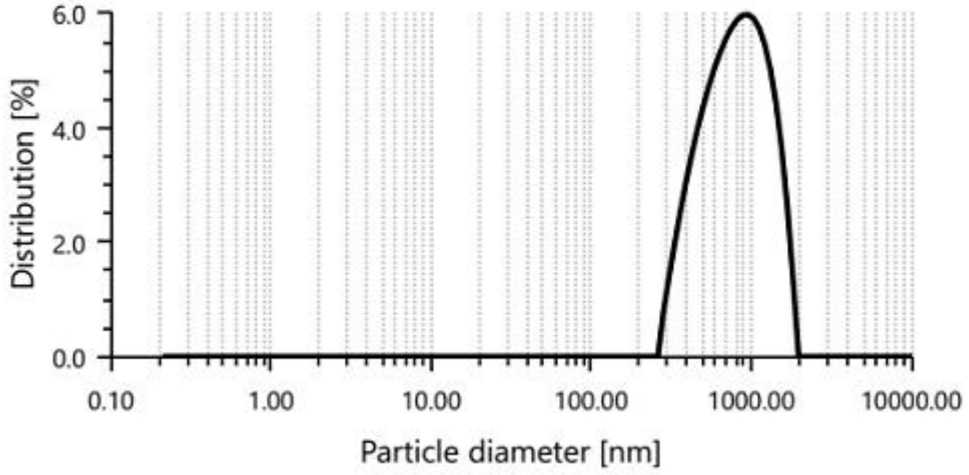

Fig. S43 Dynamic Light Scattering (DLS) size distribution of complex **2** measured in 0.1 M neutral phosphate buffer **(a)** before and **(b)** after 500 consecutive cycle cyclic voltammetry experiment.

Table S1 Crystal data and structure refinement parameters for complexes **1** and **2**.

	**Complex 1**	**Complex 2**
Empirical formula	$CuC_{10}H_{21}N_5Cl_2O_8$	$CuC_{11}H_{23}N_5Cl_2O_8$
Formula weight	473.758	487.78
Wavelength(λ)	0.71073 Å	0.71073 Å
Temperature (K)	296 (2)	296 (2)
Crystal system	Orthorhombic	Triclinic
Space group	Pbca	P-1
a[Å]	14.876(3)	7.9739(14)
b[Å]	14.641(3)	10.6041(17)
c[Å]	16.601(3)	11.6419(19)
α[°]	90	103.174(4)
β[°]	90	104.500(4)
γ[°]	90	92.334(4)
Volume[$Å^3$]	3615.7(11)	922.9(3)
Z	8	2
Density [g/cm^3]	1.741	1.755
Abs. coeff. [mm^{-1}]	1.553	1.524
F(000)	1950.6	502.0
Reflections collected	97232	17041
R_{int}	0.0819	0.0343
Data / restraints / parameters	3077/198/299	3253/0/244
Min. 2θ/°	4.908	3.966
Max. 2θ/°	49.544	49.998
Ranges (h, k, l)	$-17 \leq h \leq 17$, $-17 \leq k \leq 17$, $-19 \leq l \leq 19$	$-9 \leq h \leq 9$, $-12 \leq k \leq 12$, $-13 \leq l \leq 13$
Complete to 2θ (%)	98.9	100
Goof (F^2)	1.097	1.031
Final R indices [I>=2σ(I)]	R_1= 0.0376 wR_2= 0.0791	R_1= 0.0260 wR_2= 0.0578
R indices (all data)	R_1= 0.0490 wR_2= 0.0878	R_1= 0.0320 wR_2= 0.0605

Table S2 Crystal data and structure refinement parameters for complexes **$[Zn(L_1H)(L_2)](ClO_4)_2$** and **$[Zn(L_1H)(L_3)](ClO_4)_2$**.

	**$[Zn(L_1H)(L_2)](ClO_4)_2$**	**$[Zn(L_1H)(L_3)](ClO_4)_2$**
Empirical formula	$ZnC_{10}H_{21}N_5Cl_2O_8$	$ZnC_{11}H_{23}N_5Cl_2O_8$
Formula weight	475.59	489.61
Wavelength(λ)	0.71073 Å	0.71073 Å
Temperature (K)	293 (2)	296 (2)
Crystal system	Triclinic	Triclinic
Space group	P-1	P-1
a[Å]	8.1369(15)	8.0483(11)
b[Å]	10.5865(19)	10.7274(14)
c[Å]	11.620(2)	11.8081(15)
α[°]	105.230(4)	104.152(3)
β[°]	98.575(4)	103.590(3)
γ[°]	91.591(5)	92.363(3)
Volume[$Å^3$]	952.7(3)	955.6(2)
Z	2	2
Density [g/cm^3]	1.658	1.702
Abs. coeff. [mm^{-1}]	1.617	1.615
F(000)	488.0	504.0
Reflections collected	33598	30955
R_{int}	0.1012	0.0477
Data / restraints / parameters	3346/0/263	3364/0/245
Min. 2θ/°	3.682	3.676
Max. 2θ/°	49.986	49.990
Ranges (h, k, l)	$-9 \leq h \leq 9$, $-12 \leq k \leq 12$, $-13 \leq l \leq 13$	$-9 \leq h \leq 9$, $-12 \leq k \leq 12$, $-14 \leq l \leq 14$
Complete to 2θ (%)	100	100
Goof (F^2)	1.045	1.035
Final R indices [I>=2σ(I)]	R_1= 0.0619 wR_2= 0.1623	R_1= 0.0337 wR_2= 0.0844
R indices (all data)	R_1= 0.0871 wR_2= 0.1885	R_1= 0.0416 wR_2= 0.0904

Table S3 Gibbs free energies of intermediates and transition state of complex **1** in aqueous solution.

**Species**	**Charge**	**Spin Multiplicity**	**Gibbs free energy in aqueous solution (in kcalmol^{-1})**
Complex **1**	+2	Doublet	-541656.4326
		Quartet	-541661.9967
Complex **1a**	+2	Singlet	-541235.6028
		Triplet	-541258.2025
Complex **1b**	+2	Doublet	-588809.404
		Quartet	-588825.917
Transition State **(TS)**	+2	Singlet	-636624.5348
		Triplet	-636665.4943
Complex **1c**	+2	Singlet	-636633.559
		Triplet	-636693.591

Table S4 Gibbs free energies of intermediates and transition state of complex **2** in aqueous solution.

**Species**	**Charge**	**Spin Multiplicity**	**Gibbs free energy in aqueous solution (in $kcalmol^{-1}$)**
Complex **2**	+2	Doublet	-566308.1251
		Quartet	-566315.4657
Complex **2a**	+2	Singlet	-565887.5663
		Triplet	-565911.3935
Complex **2b**	+2	Doublet	-613460.8424
		Quartet	-613483.1014
Transition State **(TS)**	+2	Singlet	-661291.0077
		Triplet	-661326.6647
Complex **2c**	+2	Singlet	-661244.786
		Triplet	-661356.8127

Table S5 Coordinate file of the DFT optimized structure of complex **1** in doublet state.

Cu	-0.460763000000	-0.038993000000	0.110170000000
C	3.414000000000	1.027982000000	-1.449278000000
H	3.785760000000	1.636072000000	-2.266403000000
C	4.282332000000	0.371334000000	-0.573501000000
H	5.357519000000	0.460084000000	-0.692850000000
C	3.750659000000	-0.396188000000	0.461526000000
H	4.400187000000	-0.913472000000	1.160302000000
C	2.366066000000	-0.498415000000	0.596881000000
C	1.731913000000	-1.372948000000	1.656675000000
H	1.660917000000	-2.398904000000	1.276563000000
H	2.368489000000	-1.410490000000	2.546236000000
C	-2.292022000000	2.202737000000	0.806357000000
H	-2.720645000000	3.188714000000	0.601801000000
H	-2.258316000000	2.079562000000	1.893478000000
C	-3.143666000000	1.110664000000	0.172157000000
H	-4.168537000000	1.137839000000	0.558525000000
H	-3.196778000000	1.251594000000	-0.911896000000
C	-3.057054000000	-1.342953000000	-0.382339000000
H	-4.141709000000	-1.249663000000	-0.505738000000
H	-2.868515000000	-2.271530000000	0.165598000000
C	-2.359393000000	-1.382490000000	-1.737833000000
H	-2.692499000000	-2.256283000000	-2.306672000000
H	-2.601942000000	-0.496780000000	-2.331387000000
N	1.532874000000	0.146031000000	-0.250562000000
N	0.355127000000	-0.909141000000	1.977322000000
H	0.403782000000	-0.173542000000	2.682838000000
H	-0.160474000000	-1.670930000000	2.415572000000
N	-0.884979000000	-1.389721000000	-1.514397000000
H	-0.391338000000	-1.164487000000	-2.377472000000
H	-0.573526000000	-2.329076000000	-1.263756000000
N	-2.514424000000	-0.217969000000	0.439890000000
H	-2.667006000000	-0.438799000000	1.424264000000
N	-0.898855000000	2.079720000000	0.286687000000
H	-0.246129000000	2.578809000000	0.890278000000
H	-0.829131000000	2.538631000000	-0.622372000000
C	2.046790000000	0.893497000000	-1.250844000000
H	1.336070000000	1.391695000000	-1.903216000000

Table S6 Coordinate file of the DFT optimized structure of complex **1** in quartet state.

Cu	-0.460763000000	-0.038993000000	0.110170000000
C	3.414000000000	1.027982000000	-1.449278000000
H	3.785760000000	1.636072000000	-2.266403000000
C	4.282332000000	0.371334000000	-0.573501000000
H	5.357519000000	0.460084000000	-0.692850000000
C	3.750659000000	-0.396188000000	0.461526000000
H	4.400187000000	-0.913472000000	1.160302000000
C	2.366066000000	-0.498415000000	0.596881000000
C	1.731913000000	-1.372948000000	1.656675000000
H	1.660917000000	-2.398904000000	1.276563000000
H	2.368489000000	-1.410490000000	2.546236000000
C	-2.292022000000	2.202737000000	0.806357000000
H	-2.720645000000	3.188714000000	0.601801000000
H	-2.258316000000	2.079562000000	1.893478000000
C	-3.143666000000	1.110664000000	0.172157000000
H	-4.168537000000	1.137839000000	0.558525000000
H	-3.196778000000	1.251594000000	-0.911896000000
C	-3.057054000000	-1.342953000000	-0.382339000000
H	-4.141709000000	-1.249663000000	-0.505738000000
H	-2.868515000000	-2.271530000000	0.165598000000
C	-2.359393000000	-1.382490000000	-1.737833000000
H	-2.692499000000	-2.256283000000	-2.306672000000
H	-2.601942000000	-0.496780000000	-2.331387000000
N	1.532874000000	0.146031000000	-0.250562000000
N	0.355127000000	-0.909141000000	1.977322000000
H	0.403782000000	-0.173542000000	2.682838000000
H	-0.160474000000	-1.670930000000	2.415572000000
N	-0.884979000000	-1.389721000000	-1.514397000000
H	-0.391338000000	-1.164487000000	-2.377472000000
H	-0.573526000000	-2.329076000000	-1.263756000000
N	-2.514424000000	-0.217969000000	0.439890000000
H	-2.667006000000	-0.438799000000	1.424264000000
N	-0.898855000000	2.079720000000	0.286687000000
H	-0.246129000000	2.578809000000	0.890278000000
H	-0.829131000000	2.538631000000	-0.622372000000
C	2.046790000000	0.893497000000	-1.250844000000
H	1.336070000000	1.391695000000	-1.903216000000

Table S7 Coordinate file of the DFT optimized structure of complex **1a** in singlet state.

Cu	0.492257000000	0.002730000000	-0.038776000000
C	-3.435910000000	1.173293000000	1.240222000000
H	-3.839788000000	1.879389000000	1.957232000000
C	-4.268070000000	0.380672000000	0.445099000000
H	-5.347568000000	0.460100000000	0.526598000000
C	-3.695104000000	-0.511776000000	-0.458612000000
H	-4.316124000000	-1.136990000000	-1.092199000000
C	-2.305715000000	-0.601696000000	-0.549897000000
C	-1.628424000000	-1.600185000000	-1.461998000000
H	-1.612173000000	-2.578875000000	-0.967947000000
H	-2.204962000000	-1.726170000000	-2.384583000000
C	2.248678000000	2.089769000000	-1.065440000000
H	2.691403000000	3.087458000000	-0.995291000000
H	2.128468000000	1.832179000000	-2.119835000000
C	3.075778000000	1.033823000000	-0.359552000000
H	4.059770000000	0.893052000000	-0.839806000000
H	3.295125000000	1.328688000000	0.681381000000
C	2.960334000000	-1.311451000000	0.339183000000
H	4.055593000000	-1.239990000000	0.308143000000
H	2.671281000000	-2.272074000000	-0.100361000000
C	2.457562000000	-1.213931000000	1.776063000000
H	2.775457000000	-2.060915000000	2.391736000000
H	2.812412000000	-0.298402000000	2.255048000000
N	-1.507402000000	0.177057000000	0.218848000000
N	-0.230207000000	-1.191987000000	-1.728788000000
H	-0.184226000000	-0.574151000000	-2.538849000000
H	0.350590000000	-1.994860000000	-1.962885000000
N	0.960161000000	-1.126794000000	1.672828000000
H	0.547964000000	-0.809581000000	2.551146000000
H	0.567876000000	-2.055628000000	1.508515000000
N	2.374786000000	-0.235679000000	-0.481909000000
N	0.899449000000	2.035997000000	-0.421781000000
H	0.187317000000	2.474697000000	-1.006592000000
H	0.905552000000	2.558902000000	0.456270000000
C	-2.062034000000	1.044838000000	1.093833000000
H	-1.379499000000	1.643709000000	1.689000000000

Table S8 Coordinate file of the DFT optimized structure of complex **1a** in triplet state.

Cu	-0.496294000000	0.002897000000	0.006129000000
C	3.425230000000	0.209842000000	-1.750729000000
H	3.823792000000	0.343614000000	-2.750179000000
C	4.263623000000	0.076654000000	-0.641205000000
H	5.342330000000	0.107831000000	-0.757924000000
C	3.697563000000	-0.090975000000	0.621642000000
H	4.323907000000	-0.191492000000	1.502167000000
C	2.309191000000	-0.130374000000	0.752012000000
C	1.646954000000	-0.382764000000	2.091662000000
H	1.576569000000	-1.465500000000	2.251430000000
H	2.276241000000	0.009447000000	2.897061000000
C	-2.228430000000	2.387523000000	0.067920000000
H	-2.631464000000	3.324352000000	-0.328206000000
H	-2.079360000000	2.515672000000	1.143132000000
C	-3.193250000000	1.226012000000	-0.190762000000
H	-4.080524000000	1.280213000000	0.457303000000
H	-3.579134000000	1.247493000000	-1.226092000000
C	-3.159388000000	-1.292513000000	-0.073866000000
H	-4.137392000000	-1.227938000000	-0.565502000000
H	-3.344770000000	-1.615469000000	0.965277000000
C	-2.233999000000	-2.289460000000	-0.789946000000
H	-2.588291000000	-3.314545000000	-0.646686000000
H	-2.231553000000	-2.080742000000	-1.862739000000
N	1.504623000000	0.006462000000	-0.326466000000
N	0.271045000000	0.174956000000	2.129922000000
H	0.314089000000	1.157500000000	2.399436000000
H	-0.259529000000	-0.281121000000	2.870232000000
N	-0.838142000000	-2.106862000000	-0.291885000000
H	-0.174838000000	-2.532172000000	-0.938949000000
H	-0.712589000000	-2.596116000000	0.595069000000
N	-2.484855000000	-0.016468000000	-0.013756000000
N	-0.903973000000	2.050808000000	-0.534164000000
H	-0.189108000000	2.702080000000	-0.210970000000
H	-0.938204000000	2.161646000000	-1.549012000000
C	2.052147000000	0.174132000000	-1.550797000000
H	1.363612000000	0.280499000000	-2.383717000000

Table S9 Coordinate file of the DFT optimized structure of complex **1b** in doublet state.

Cu	-0.450344000000	0.011237000000	-0.064356000000
C	3.608301000000	0.339775000000	-1.518096000000
H	4.072572000000	0.557870000000	-2.473360000000
C	4.370693000000	0.109230000000	-0.372232000000
H	5.454712000000	0.147966000000	-0.413441000000
C	3.723756000000	-0.169090000000	0.833113000000
H	4.290235000000	-0.350933000000	1.740812000000
C	2.331879000000	-0.213967000000	0.861275000000
C	1.562304000000	-0.582823000000	2.110434000000
H	1.468059000000	-1.673543000000	2.169726000000
H	2.108828000000	-0.266697000000	3.004753000000
C	-2.171395000000	2.369763000000	0.520206000000
H	-2.571647000000	3.348954000000	0.237371000000
H	-2.023892000000	2.372427000000	1.603401000000
C	-3.153570000000	1.253896000000	0.131272000000
H	-4.025562000000	1.231452000000	0.799668000000
H	-3.554240000000	1.420572000000	-0.885085000000
C	-3.113639000000	-1.288218000000	0.126317000000
H	-4.121481000000	-1.239880000000	-0.306467000000
H	-3.242978000000	-1.583443000000	1.182719000000
C	-2.227891000000	-2.299723000000	-0.620391000000
H	-2.555134000000	-3.323276000000	-0.415354000000
H	-2.305254000000	-2.133465000000	-1.696405000000
N	1.605178000000	0.020284000000	-0.253482000000
N	0.192372000000	-0.006654000000	2.071216000000
H	0.223381000000	0.962044000000	2.387840000000
H	-0.409832000000	-0.494300000000	2.732849000000
N	-0.797920000000	-2.096496000000	-0.243327000000
H	-0.184252000000	-2.421824000000	-0.989735000000
H	-0.558519000000	-2.622896000000	0.596602000000
N	-2.452855000000	-0.005179000000	0.114866000000
N	-0.854284000000	2.113098000000	-0.117941000000
H	-0.130700000000	2.733752000000	0.242800000000
H	-0.878347000000	2.221043000000	-1.134577000000
C	2.221654000000	0.289756000000	-1.420086000000
H	1.572063000000	0.456375000000	-2.273048000000
O	-0.605633000000	0.159265000000	-2.084124000000
H	-1.482982000000	0.030840000000	-2.481969000000

Table S10 Coordinate file of the DFT optimized structure of complex **1b** in quartet state.

Cu	-0.450344000000	0.011237000000	-0.064356000000
C	3.608301000000	0.339775000000	-1.518096000000
H	4.072572000000	0.557870000000	-2.473360000000
C	4.370693000000	0.109230000000	-0.372232000000
H	5.454712000000	0.147966000000	-0.413441000000
C	3.723756000000	-0.169090000000	0.833113000000
H	4.290235000000	-0.350933000000	1.740812000000
C	2.331879000000	-0.213967000000	0.861275000000
C	1.562304000000	-0.582823000000	2.110434000000
H	1.468059000000	-1.673543000000	2.169726000000
H	2.108828000000	-0.266697000000	3.004753000000
C	-2.171395000000	2.369763000000	0.520206000000
H	-2.571647000000	3.348954000000	0.237371000000
H	-2.023892000000	2.372427000000	1.603401000000
C	-3.153570000000	1.253896000000	0.131272000000
H	-4.025562000000	1.231452000000	0.799668000000
H	-3.554240000000	1.420572000000	-0.885085000000
C	-3.113639000000	-1.288218000000	0.126317000000
H	-4.121481000000	-1.239880000000	-0.306467000000
H	-3.242978000000	-1.583443000000	1.182719000000
C	-2.227891000000	-2.299723000000	-0.620391000000
H	-2.555134000000	-3.323276000000	-0.415354000000
H	-2.305254000000	-2.133465000000	-1.696405000000
N	1.605178000000	0.020284000000	-0.253482000000
N	0.192372000000	-0.006654000000	2.071216000000
H	0.223381000000	0.962044000000	2.387840000000
H	-0.409832000000	-0.494300000000	2.732849000000
N	-0.797920000000	-2.096496000000	-0.243327000000
H	-0.184252000000	-2.421824000000	-0.989735000000
H	-0.558519000000	-2.622896000000	0.596602000000
N	-2.452855000000	-0.005179000000	0.114866000000
N	-0.854284000000	2.113098000000	-0.117941000000
H	-0.130700000000	2.733752000000	0.242800000000
H	-0.878347000000	2.221043000000	-1.134577000000
C	2.221654000000	0.289756000000	-1.420086000000
H	1.572063000000	0.456375000000	-2.273048000000
O	-0.605633000000	0.159265000000	-2.084124000000
H	-1.482982000000	0.030840000000	-2.481969000000

Table S11 Coordinate file of the DFT optimized structure of Transition State (**TS**) of complex **1** in singlet state.

Cu	-0.285783000000	-0.016740000000	0.039582000000
C	3.740826000000	-0.487886000000	1.354418000000
H	4.239071000000	-0.807770000000	2.262575000000
C	4.458717000000	-0.117357000000	0.216912000000
H	5.543943000000	-0.144963000000	0.219389000000
C	3.771321000000	0.280521000000	-0.933162000000
H	4.306150000000	0.562766000000	-1.834201000000
C	2.381086000000	0.306933000000	-0.912300000000
C	1.521339000000	0.754147000000	-2.063496000000
H	1.368223000000	1.837242000000	-2.018102000000
H	1.982151000000	0.526033000000	-3.029639000000
C	-2.098049000000	-2.358368000000	-1.080881000000
H	-2.761845000000	-3.223858000000	-0.980885000000
H	-1.691364000000	-2.402449000000	-2.095821000000
C	-2.946643000000	-1.062730000000	-0.932663000000
H	-3.154304000000	-0.633966000000	-1.916047000000
H	-3.918426000000	-1.292944000000	-0.480860000000
C	-2.867794000000	1.357671000000	-0.345591000000
H	-3.946633000000	1.353351000000	-0.143513000000
H	-2.741758000000	1.576438000000	-1.411198000000
C	-2.172469000000	2.415022000000	0.510620000000
H	-2.658864000000	3.381304000000	0.335927000000
H	-2.311998000000	2.184717000000	1.571869000000
N	1.704271000000	-0.053825000000	0.203806000000
N	0.185159000000	0.099264000000	-1.939855000000
H	0.234030000000	-0.865143000000	-2.270979000000
H	-0.502643000000	0.580155000000	-2.518438000000
N	-0.722769000000	2.428344000000	0.217642000000
H	-0.209312000000	2.809979000000	1.009459000000
H	-0.538154000000	3.065824000000	-0.555558000000
N	-2.317098000000	-0.001738000000	-0.089719000000
N	-0.959680000000	-2.386309000000	-0.144352000000
H	-0.308278000000	-3.116992000000	-0.424948000000
H	-1.265201000000	-2.652082000000	0.791776000000
C	2.349229000000	-0.447574000000	1.308329000000
H	1.728591000000	-0.727868000000	2.156722000000
O	-0.331762000000	-0.210521000000	1.933211000000
H	-2.467503000000	-0.240995000000	0.899461000000
O	-1.540596000000	-0.532767000000	2.528201000000
H	-1.713876000000	0.220536000000	3.128063000000

Table S12 Coordinate file of the DFT optimized structure of Transition State (**TS**) of complex **1** in triplet state.

Cu	-0.285783000000	-0.016740000000	0.039582000000
C	3.740826000000	-0.487886000000	1.354418000000
H	4.239071000000	-0.807770000000	2.262575000000
C	4.458717000000	-0.117357000000	0.216912000000
H	5.543943000000	-0.144963000000	0.219389000000
C	3.771321000000	0.280521000000	-0.933162000000
H	4.306150000000	0.562766000000	-1.834201000000
C	2.381086000000	0.306933000000	-0.912300000000
C	1.521339000000	0.754147000000	-2.063496000000
H	1.368223000000	1.837242000000	-2.018102000000
H	1.982151000000	0.526033000000	-3.029639000000
C	-2.098049000000	-2.358368000000	-1.080881000000
H	-2.761845000000	-3.223858000000	-0.980885000000
H	-1.691364000000	-2.402449000000	-2.095821000000
C	-2.946643000000	-1.062730000000	-0.932663000000
H	-3.154304000000	-0.633966000000	-1.916047000000
H	-3.918426000000	-1.292944000000	-0.480860000000
C	-2.867794000000	1.357671000000	-0.345591000000
H	-3.946633000000	1.353351000000	-0.143513000000
H	-2.741758000000	1.576438000000	-1.411198000000
C	-2.172469000000	2.415022000000	0.510620000000
H	-2.658864000000	3.381304000000	0.335927000000
H	-2.311998000000	2.184717000000	1.571869000000
N	1.704271000000	-0.053825000000	0.203806000000
N	0.185159000000	0.099264000000	-1.939855000000
H	0.234030000000	-0.865143000000	-2.270979000000
H	-0.502643000000	0.580155000000	-2.518438000000
N	-0.722769000000	2.428344000000	0.217642000000
H	-0.209312000000	2.809979000000	1.009459000000
H	-0.538154000000	3.065824000000	-0.555558000000
N	-2.317098000000	-0.001738000000	-0.089719000000
N	-0.959680000000	-2.386309000000	-0.144352000000
H	-0.308278000000	-3.116992000000	-0.424948000000
H	-1.265201000000	-2.652082000000	0.791776000000
C	2.349229000000	-0.447574000000	1.308329000000
H	1.728591000000	-0.727868000000	2.156722000000
O	-0.331762000000	-0.210521000000	1.933211000000
H	-2.467503000000	-0.240995000000	0.899461000000
O	-1.540596000000	-0.532767000000	2.528201000000
H	-1.713876000000	0.220536000000	3.128063000000

Table S13 Coordinate file of the DFT optimized structure of complex **1c** in singlet state.

Cu	-0.289308000000	-0.097970000000	0.055133000000
C	3.773377000000	-0.901870000000	1.100141000000
H	4.286907000000	-1.493452000000	1.849541000000
C	4.471575000000	-0.156811000000	0.149501000000
H	5.557087000000	-0.158794000000	0.138826000000
C	3.762861000000	0.592907000000	-0.792453000000
H	4.281203000000	1.182347000000	-1.541815000000
C	2.372289000000	0.579666000000	-0.759016000000
C	1.498497000000	1.398676000000	-1.668966000000
H	1.363510000000	2.400642000000	-1.249390000000
H	1.941639000000	1.516762000000	-2.662818000000
C	-2.199198000000	-1.642190000000	-1.850637000000
H	-2.795556000000	-2.425968000000	-2.333707000000
H	-1.997703000000	-0.885728000000	-2.617443000000
C	-3.046726000000	-1.041034000000	-0.726756000000
H	-3.962269000000	-0.613006000000	-1.145057000000
H	-3.346170000000	-1.838408000000	-0.039205000000
C	-2.831200000000	1.395276000000	-0.063844000000
H	-3.923042000000	1.400418000000	0.047089000000
H	-2.613298000000	1.740487000000	-1.080458000000
C	-2.200022000000	2.292917000000	1.002004000000
H	-2.583361000000	3.310975000000	0.877236000000
H	-2.527894000000	1.954609000000	1.990417000000
N	1.713434000000	-0.158031000000	0.165680000000
N	0.157658000000	0.751846000000	-1.754844000000
H	0.186454000000	-0.020111000000	-2.420361000000
H	-0.536898000000	1.415954000000	-2.093731000000
N	-0.720676000000	2.223954000000	0.940268000000
H	-0.333572000000	2.259054000000	1.880912000000
H	-0.356335000000	3.050934000000	0.471791000000
N	-2.337368000000	-0.000982000000	0.086899000000
N	-0.913686000000	-2.119765000000	-1.309059000000
H	-0.320274000000	-2.464064000000	-2.063033000000
H	-1.066924000000	-2.933284000000	-0.713122000000
C	2.381230000000	-0.878709000000	1.075059000000
H	1.781675000000	-1.429961000000	1.795496000000
O	-0.277154000000	-0.922718000000	1.762046000000
H	-2.429979000000	-0.254429000000	1.077940000000
O	-1.445956000000	-1.026852000000	2.490278000000
H	-1.622032000000	-1.989425000000	2.520617000000

Table S14 Coordinate file of the DFT optimized structure of complex **1c** in triplet state.

Cu	-0.289308000000	-0.097970000000	0.055133000000
C	3.773377000000	-0.901870000000	1.100141000000
H	4.286907000000	-1.493452000000	1.849541000000
C	4.471575000000	-0.156811000000	0.149501000000
H	5.557087000000	-0.158794000000	0.138826000000
C	3.762861000000	0.592907000000	-0.792453000000
H	4.281203000000	1.182347000000	-1.541815000000
C	2.372289000000	0.579666000000	-0.759016000000
C	1.498497000000	1.398676000000	-1.668966000000
H	1.363510000000	2.400642000000	-1.249390000000
H	1.941639000000	1.516762000000	-2.662818000000
C	-2.199198000000	-1.642190000000	-1.850637000000
H	-2.795556000000	-2.425968000000	-2.333707000000
H	-1.997703000000	-0.885728000000	-2.617443000000
C	-3.046726000000	-1.041034000000	-0.726756000000
H	-3.962269000000	-0.613006000000	-1.145057000000
H	-3.346170000000	-1.838408000000	-0.039205000000
C	-2.831200000000	1.395276000000	-0.063844000000
H	-3.923042000000	1.400418000000	0.047089000000
H	-2.613298000000	1.740487000000	-1.080458000000
C	-2.200022000000	2.292917000000	1.002004000000
H	-2.583361000000	3.310975000000	0.877236000000
H	-2.527894000000	1.954609000000	1.990417000000
N	1.713434000000	-0.158031000000	0.165680000000
N	0.157658000000	0.751846000000	-1.754844000000
H	0.186454000000	-0.020111000000	-2.420361000000
H	-0.536898000000	1.415954000000	-2.093731000000
N	-0.720676000000	2.223954000000	0.940268000000
H	-0.333572000000	2.259054000000	1.880912000000
H	-0.356335000000	3.050934000000	0.471791000000
N	-2.337368000000	-0.000982000000	0.086899000000
N	-0.913686000000	-2.119765000000	-1.309059000000
H	-0.320274000000	-2.464064000000	-2.063033000000
H	-1.066924000000	-2.933284000000	-0.713122000000
C	2.381230000000	-0.878709000000	1.075059000000
H	1.781675000000	-1.429961000000	1.795496000000
O	-0.277154000000	-0.922718000000	1.762046000000
H	-2.429979000000	-0.254429000000	1.077940000000
O	-1.445956000000	-1.026852000000	2.490278000000
H	-1.622032000000	-1.989425000000	2.520617000000

Table S15 Coordinate file of the DFT optimized structure of complex **2** in doublet state.

Cu	-0.512375000000	0.134177000000	-0.019763000000
N	1.475782000000	-0.362397000000	0.042422000000
N	-0.142420000000	2.284989000000	0.034428000000
H	-0.702756000000	2.738317000000	0.756783000000
H	-0.462821000000	2.695131000000	-0.844499000000
N	-0.999388000000	-0.501042000000	2.038554000000
H	-0.398813000000	-0.064070000000	2.736832000000
H	-0.876185000000	-1.504745000000	2.172372000000
N	-2.613471000000	0.343525000000	-0.127190000000
H	-2.858590000000	1.325415000000	-0.001810000000
N	-0.837161000000	-0.968619000000	-1.844577000000
H	-0.275757000000	-1.816910000000	-1.909096000000
H	-0.544470000000	-0.392426000000	-2.635151000000
C	1.743217000000	-1.637794000000	0.407018000000
H	0.888077000000	-2.264602000000	0.641471000000
C	3.029525000000	-2.149490000000	0.480696000000
H	3.188069000000	-3.179932000000	0.778559000000
C	4.094483000000	-1.303974000000	0.164198000000
H	5.118636000000	-1.660600000000	0.210726000000
C	3.821457000000	0.007771000000	-0.212989000000
H	4.629038000000	0.686365000000	-0.465551000000
C	2.500485000000	0.466652000000	-0.274588000000
C	2.209576000000	1.887324000000	-0.703720000000
H	1.791419000000	1.892780000000	-1.720952000000
H	3.159647000000	2.422238000000	-0.776375000000
C	1.286238000000	2.672067000000	0.232855000000
H	1.545500000000	2.481277000000	1.277733000000
H	1.411428000000	3.744480000000	0.052649000000
C	-2.424677000000	-0.140527000000	2.278903000000
H	-2.467946000000	0.923591000000	2.532719000000
H	-2.854729000000	-0.691980000000	3.121065000000
C	-3.217718000000	-0.416382000000	1.008071000000
H	-3.181316000000	-1.482571000000	0.763697000000
H	-4.271519000000	-0.144614000000	1.136328000000
C	-3.088142000000	-0.082110000000	-1.479005000000
H	-2.938640000000	0.760692000000	-2.161439000000
H	-4.160541000000	-0.306946000000	-1.465853000000
C	-2.288282000000	-1.289057000000	-1.955458000000
H	-2.484858000000	-2.163552000000	-1.329285000000
H	-2.574369000000	-1.550539000000	-2.979128000000

Table S16 Coordinate file of the DFT optimized structure of complex **2** in quartet state.

Cu	-0.512375000000	0.134177000000	-0.019763000000
N	1.475782000000	-0.362397000000	0.042422000000
N	-0.142420000000	2.284989000000	0.034428000000
H	-0.702756000000	2.738317000000	0.756783000000
H	-0.462821000000	2.695131000000	-0.844499000000
N	-0.999388000000	-0.501042000000	2.038554000000
H	-0.398813000000	-0.064070000000	2.736832000000
H	-0.876185000000	-1.504745000000	2.172372000000
N	-2.613471000000	0.343525000000	-0.127190000000
H	-2.858590000000	1.325415000000	-0.001810000000
N	-0.837161000000	-0.968619000000	-1.844577000000
H	-0.275757000000	-1.816910000000	-1.909096000000
H	-0.544470000000	-0.392426000000	-2.635151000000
C	1.743217000000	-1.637794000000	0.407018000000
H	0.888077000000	-2.264602000000	0.641471000000
C	3.029525000000	-2.149490000000	0.480696000000
H	3.188069000000	-3.179932000000	0.778559000000
C	4.094483000000	-1.303974000000	0.164198000000
H	5.118636000000	-1.660600000000	0.210726000000
C	3.821457000000	0.007771000000	-0.212989000000
H	4.629038000000	0.686365000000	-0.465551000000
C	2.500485000000	0.466652000000	-0.274588000000
C	2.209576000000	1.887324000000	-0.703720000000
H	1.791419000000	1.892780000000	-1.720952000000
H	3.159647000000	2.422238000000	-0.776375000000
C	1.286238000000	2.672067000000	0.232855000000
H	1.545500000000	2.481277000000	1.277733000000
H	1.411428000000	3.744480000000	0.052649000000
C	-2.424677000000	-0.140527000000	2.278903000000
H	-2.467946000000	0.923591000000	2.532719000000
H	-2.854729000000	-0.691980000000	3.121065000000
C	-3.217718000000	-0.416382000000	1.008071000000
H	-3.181316000000	-1.482571000000	0.763697000000
H	-4.271519000000	-0.144614000000	1.136328000000
C	-3.088142000000	-0.082110000000	-1.479005000000
H	-2.938640000000	0.760692000000	-2.161439000000
H	-4.160541000000	-0.306946000000	-1.465853000000
C	-2.288282000000	-1.289057000000	-1.955458000000
H	-2.484858000000	-2.163552000000	-1.329285000000
H	-2.574369000000	-1.550539000000	-2.979128000000

Table S17 Coordinate file of the DFT optimized structure of complex **2a** in singlet state.

Cu	-0.539336000000	0.051492000000	-0.007791000000
N	1.464658000000	-0.334213000000	-0.039498000000
N	-0.272240000000	2.160217000000	0.068282000000
H	-1.021679000000	2.460811000000	0.691585000000
H	-0.486483000000	2.570621000000	-0.841568000000
N	-0.904453000000	-0.880371000000	1.885622000000
H	-0.229606000000	-0.600668000000	2.597885000000
H	-0.823130000000	-1.895408000000	1.809286000000
N	-2.477017000000	0.424629000000	0.062720000000
N	-0.992836000000	-0.829989000000	-1.881747000000
H	-0.569319000000	-1.736632000000	-2.081410000000
H	-0.595888000000	-0.191745000000	-2.573627000000
C	1.767735000000	-1.650615000000	0.070376000000
H	0.930881000000	-2.337170000000	0.153196000000
C	3.067258000000	-2.130745000000	0.077550000000
H	3.251301000000	-3.195508000000	0.168251000000
C	4.110753000000	-1.208882000000	-0.027745000000
H	5.145303000000	-1.537531000000	-0.020051000000
C	3.801473000000	0.142145000000	-0.142740000000
H	4.590974000000	0.880696000000	-0.231105000000
C	2.467195000000	0.570800000000	-0.155075000000
C	2.169158000000	2.043885000000	-0.342336000000
H	1.939417000000	2.233388000000	-1.401584000000
H	3.087273000000	2.601661000000	-0.141866000000
C	1.056429000000	2.625205000000	0.529298000000
H	1.179793000000	2.327263000000	1.574402000000
H	1.106074000000	3.719432000000	0.490742000000
C	-2.297613000000	-0.506800000000	2.276851000000
H	-2.272074000000	0.499232000000	2.701321000000
C	-3.099284000000	-0.512406000000	0.990742000000
H	-3.208185000000	-1.537798000000	0.599377000000
H	-4.127538000000	-0.143985000000	1.151148000000
C	-3.021200000000	0.340300000000	-1.304389000000
H	-2.744563000000	1.253551000000	-1.842041000000
H	-4.118558000000	0.312681000000	-1.246114000000
C	-2.486661000000	-0.887153000000	-2.034741000000
H	-2.835351000000	-1.809925000000	-1.566188000000
H	-2.788158000000	-0.912580000000	-3.086321000000
H	-2.707974000000	-1.189973000000	3.026234000000

Table S18 Coordinate file of the DFT optimized structure of complex **2a** in triplet state.

Cu	-0.532765000000	0.013365000000	-0.028505000000
N	1.455315000000	-0.372086000000	0.120794000000
N	-0.279451000000	2.162441000000	-0.531241000000
H	-0.883977000000	2.760988000000	0.031307000000
H	-0.584820000000	2.323918000000	-1.491983000000
N	-0.957942000000	0.079349000000	2.116633000000
H	-0.273657000000	0.633133000000	2.630903000000
H	-0.937340000000	-0.847839000000	2.543596000000
N	-2.529671000000	-0.120489000000	-0.026461000000
N	-0.844078000000	-1.087649000000	-1.878909000000
H	-0.158304000000	-1.828865000000	-2.018443000000
H	-0.730486000000	-0.456763000000	-2.673065000000
C	1.772768000000	-1.522808000000	0.760350000000
H	0.942169000000	-2.122695000000	1.121236000000
C	3.079314000000	-1.943270000000	0.954935000000
H	3.278563000000	-2.875646000000	1.471239000000
C	4.110235000000	-1.135405000000	0.471760000000
H	5.148534000000	-1.422551000000	0.605018000000
C	3.785594000000	0.048085000000	-0.185856000000
H	4.567117000000	0.693333000000	-0.572311000000
C	2.447357000000	0.418095000000	-0.361422000000
C	2.105954000000	1.690693000000	-1.106316000000
H	1.715852000000	1.442000000000	-2.104179000000
H	3.036638000000	2.234137000000	-1.286957000000
C	1.128193000000	2.637890000000	-0.400617000000
H	1.365505000000	2.708650000000	0.664773000000
H	1.232028000000	3.641789000000	-0.825086000000
C	-2.317487000000	0.676543000000	2.251752000000
H	-2.229754000000	1.748370000000	2.055955000000
C	-3.242774000000	0.029182000000	1.217336000000
H	-3.565513000000	-0.976203000000	1.543267000000
H	-4.170037000000	0.605718000000	1.080634000000
C	-3.179523000000	-0.648784000000	-1.204949000000
H	-3.368451000000	0.198516000000	-1.885580000000
H	-4.150573000000	-1.106079000000	-0.982698000000
C	-2.223163000000	-1.656945000000	-1.864707000000
H	-2.196432000000	-2.575301000000	-1.272636000000
H	-2.572820000000	-1.914043000000	-2.869104000000
H	-2.722061000000	0.557212000000	3.261445000000

Table S19 Coordinate file of the DFT optimized structure of complex **2b** in doublet state.

Cu	0.507650000000	-0.080455000000	-0.001572000000
N	-1.608544000000	-0.545295000000	-0.050740000000
N	0.127125000000	1.970272000000	0.172398000000
H	0.877759000000	2.467998000000	-0.308794000000
H	0.188137000000	2.255468000000	1.151092000000
N	0.955889000000	0.263785000000	-2.125114000000
H	0.435247000000	0.997534000000	-2.592296000000
H	0.691227000000	-0.646479000000	-2.502948000000
N	2.591181000000	-0.290079000000	0.045697000000
N	0.993549000000	-0.280089000000	2.184839000000
H	0.234271000000	-0.647473000000	2.754230000000
H	1.163096000000	0.678822000000	2.478654000000
C	-1.863425000000	-1.841644000000	-0.312201000000
H	-0.991817000000	-2.476477000000	-0.460440000000
C	-3.153297000000	-2.354833000000	-0.399694000000
H	-3.306043000000	-3.407782000000	-0.608710000000
C	-4.223142000000	-1.480148000000	-0.217051000000
H	-5.247480000000	-1.834244000000	-0.280604000000
C	-3.960366000000	-0.135202000000	0.044095000000
H	-4.776572000000	0.565748000000	0.184311000000
C	-2.639803000000	0.314059000000	0.128889000000
C	-2.348458000000	1.766414000000	0.449078000000
H	-2.149186000000	1.878136000000	1.525678000000
H	-3.254899000000	2.348464000000	0.264299000000
C	-1.208101000000	2.407179000000	-0.342367000000
H	-1.274931000000	2.139735000000	-1.400051000000
H	-1.274127000000	3.497940000000	-0.271276000000
C	2.421443000000	0.487824000000	-2.248995000000
H	2.609917000000	1.544951000000	-2.042475000000
H	2.768177000000	0.280151000000	-3.267091000000
C	3.198461000000	-0.400751000000	-1.265303000000
H	3.256426000000	-1.438842000000	-1.604960000000
H	4.226945000000	-0.021832000000	-1.165019000000
C	3.181788000000	-0.981138000000	1.155425000000
H	4.077115000000	-0.369254000000	1.384445000000
H	3.601945000000	-1.961039000000	0.876938000000
C	2.234084000000	-1.084307000000	2.376832000000
H	1.948371000000	-2.128496000000	2.525522000000
H	2.751059000000	-0.768207000000	3.287842000000
O	1.190809000000	-1.840215000000	-0.414868000000
H	1.527379000000	-2.407513000000	0.292709000000

Table S20 Coordinate file of the DFT optimized structure of complex **2b** in quartet state.

Cu	0.497894000000	-0.086626000000	0.007371000000
N	-1.587638000000	-0.337955000000	0.033947000000
N	0.339651000000	2.042274000000	-0.590834000000
H	0.925631000000	2.227060000000	-1.404419000000
H	0.748187000000	2.599390000000	0.160246000000
N	0.945982000000	-0.764764000000	-1.970469000000
H	0.262099000000	-0.543354000000	-2.693038000000
H	0.904005000000	-1.769970000000	-1.791298000000
N	2.516631000000	-0.000001000000	0.050066000000
N	0.782466000000	0.274029000000	2.101564000000
H	0.137073000000	-0.371886000000	2.555225000000
H	0.543642000000	1.213281000000	2.417736000000
C	-2.030230000000	-1.611561000000	-0.056555000000
H	-1.267309000000	-2.378570000000	-0.112913000000
C	-3.380740000000	-1.937583000000	-0.051457000000
H	-3.681147000000	-2.976785000000	-0.125077000000
C	-4.314304000000	-0.909059000000	0.054252000000
H	-5.378851000000	-1.121266000000	0.063673000000
C	-3.856323000000	0.403410000000	0.149351000000
H	-4.557716000000	1.226389000000	0.235399000000
C	-2.485582000000	0.675493000000	0.139421000000
C	-2.006178000000	2.105462000000	0.246799000000
H	-1.546016000000	2.282406000000	1.229748000000
H	-2.880153000000	2.760283000000	0.215081000000
C	-1.039048000000	2.541808000000	-0.856830000000
H	-1.361199000000	2.151454000000	-1.825909000000
H	-1.029310000000	3.634642000000	-0.929002000000
C	2.311248000000	-0.315828000000	-2.350397000000
H	2.239860000000	0.716507000000	-2.702549000000
H	2.713716000000	-0.912645000000	-3.175530000000
C	3.244594000000	-0.392049000000	-1.130027000000
H	3.599251000000	-1.426483000000	-0.973039000000
H	4.148915000000	0.215183000000	-1.277184000000
C	3.139110000000	0.335745000000	1.308354000000
H	3.308987000000	1.426978000000	1.315172000000
H	4.127217000000	-0.129527000000	1.421281000000
C	2.191558000000	-0.079123000000	2.448107000000
H	2.227544000000	-1.161690000000	2.577424000000
H	2.499427000000	0.383749000000	3.390170000000
O	0.705799000000	-2.066302000000	0.476623000000
H	1.606563000000	-2.397941000000	0.626027000000

Table S21 Coordinate file of the DFT optimized structure of Transition State (**TS**) complex **2** in singlet state.

Cu	-0.332748000000	-0.069373000000	-0.000526000000
N	1.687291000000	-0.270292000000	-0.075542000000
N	-0.260028000000	1.433154000000	1.365907000000
H	-0.673945000000	1.031717000000	2.206503000000
H	-0.898854000000	2.154083000000	1.033831000000
N	-0.939685000000	-1.691054000000	1.854041000000
H	-0.877780000000	-1.518206000000	2.856766000000
H	-0.380285000000	-2.526036000000	1.694444000000
N	-2.404279000000	-0.008271000000	-0.072102000000
N	-0.945323000000	1.443114000000	-1.870075000000
H	-1.193878000000	0.862887000000	-2.669610000000
H	-0.272173000000	2.112463000000	-2.237590000000
C	2.178908000000	-1.497134000000	-0.326037000000
H	1.460308000000	-2.302048000000	-0.441685000000
C	3.542217000000	-1.737086000000	-0.448429000000
H	3.893465000000	-2.744129000000	-0.642891000000
C	4.420468000000	-0.663456000000	-0.311345000000
H	5.492144000000	-0.807931000000	-0.407872000000
C	3.902893000000	0.600668000000	-0.037907000000
H	4.560248000000	1.455203000000	0.081326000000
C	2.522703000000	0.784118000000	0.083922000000
C	1.952520000000	2.132123000000	0.447725000000
H	1.393521000000	2.564229000000	-0.391796000000
H	2.775846000000	2.819707000000	0.653987000000
C	1.057857000000	2.052946000000	1.688085000000
H	1.534681000000	1.452756000000	2.465988000000
H	0.886560000000	3.055347000000	2.093828000000
C	-2.344334000000	-1.963625000000	1.477361000000
H	-2.916904000000	-2.447116000000	2.276729000000
H	-2.348082000000	-2.645282000000	0.622435000000
C	-3.048807000000	-0.660383000000	1.100390000000
H	-4.103535000000	-0.855466000000	0.867038000000
H	-3.031768000000	0.050571000000	1.934340000000
C	-3.007806000000	1.306037000000	-0.421604000000
H	-3.197653000000	1.847885000000	0.509481000000
H	-3.991806000000	1.112207000000	-0.864154000000
C	-2.141417000000	2.168764000000	-1.390216000000
H	-2.773181000000	2.526740000000	-2.209201000000
H	-1.794455000000	3.064212000000	-0.865642000000
O	-1.431929000000	-1.791976000000	-1.972916000000
H	-2.491823000000	-0.634692000000	-0.880157000000
O	-0.388735000000	-1.635688000000	-1.078235000000
H	-1.012692000000	-1.680372000000	-2.848917000000

Table S22 Coordinate file of the DFT optimized structure of Transition State (**TS**) complex **2** in triplet state.

Cu	-0.332748000000	-0.069373000000	-0.000526000000
N	1.687291000000	-0.270292000000	-0.075542000000
N	-0.260028000000	1.433154000000	1.365907000000
H	-0.673945000000	1.031717000000	2.206503000000
H	-0.898854000000	2.154083000000	1.033831000000
N	-0.939685000000	-1.691054000000	1.854041000000
H	-0.877780000000	-1.518206000000	2.856766000000
H	-0.380285000000	-2.526036000000	1.694444000000
N	-2.404279000000	-0.008271000000	-0.072102000000
N	-0.945323000000	1.443114000000	-1.870075000000
H	-1.193878000000	0.862887000000	-2.669610000000
H	-0.272173000000	2.112463000000	-2.237590000000
C	2.178908000000	-1.497134000000	-0.326037000000
H	1.460308000000	-2.302048000000	-0.441685000000
C	3.542217000000	-1.737086000000	-0.448429000000
H	3.893465000000	-2.744129000000	-0.642891000000
C	4.420468000000	-0.663456000000	-0.311345000000
H	5.492144000000	-0.807931000000	-0.407872000000
C	3.902893000000	0.600668000000	-0.037907000000
H	4.560248000000	1.455203000000	0.081326000000
C	2.522703000000	0.784118000000	0.083922000000
C	1.952520000000	2.132123000000	0.447725000000
H	1.393521000000	2.564229000000	-0.391796000000
H	2.775846000000	2.819707000000	0.653987000000
C	1.057857000000	2.052946000000	1.688085000000
H	1.534681000000	1.452756000000	2.465988000000
H	0.886560000000	3.055347000000	2.093828000000
C	-2.344334000000	-1.963625000000	1.477361000000
H	-2.916904000000	-2.447116000000	2.276729000000
H	-2.348082000000	-2.645282000000	0.622435000000
C	-3.048807000000	-0.660383000000	1.100390000000
H	-4.103535000000	-0.855466000000	0.867038000000
H	-3.031768000000	0.050571000000	1.934340000000
C	-3.007806000000	1.306037000000	-0.421604000000
H	-3.197653000000	1.847885000000	0.509481000000
H	-3.991806000000	1.112207000000	-0.864154000000
C	-2.141417000000	2.168764000000	-1.390216000000
H	-2.773181000000	2.526740000000	-2.209201000000
H	-1.794455000000	3.064212000000	-0.865642000000
O	-1.431929000000	-1.791976000000	-1.972916000000
H	-2.491823000000	-0.634692000000	-0.880157000000
O	-0.388735000000	-1.635688000000	-1.078235000000
H	-1.012692000000	-1.680372000000	-2.848917000000

Table S23 Coordinate file of the DFT optimized structure of complex **2c** in singlet state.

Cu	-0.334939000000	-0.039356000000	0.003667000000
N	1.687699000000	-0.273725000000	-0.099475000000
N	-0.212001000000	1.454184000000	1.380952000000
H	-0.669889000000	1.089914000000	2.215159000000
H	-0.801486000000	2.200914000000	1.015119000000
N	-0.928409000000	-1.608943000000	1.931539000000
H	-0.719062000000	-1.417316000000	2.910099000000
H	-0.470847000000	-2.495541000000	1.732760000000
N	-2.401106000000	0.006083000000	-0.009495000000
N	-0.843686000000	1.679800000000	-1.791728000000
H	-0.396819000000	1.420226000000	-2.668674000000
H	-0.578166000000	2.652370000000	-1.644619000000
C	2.139274000000	-1.502627000000	-0.404317000000
H	1.394010000000	-2.281253000000	-0.536193000000
C	3.493234000000	-1.779158000000	-0.555387000000
H	3.810443000000	-2.787871000000	-0.794340000000
C	4.405448000000	-0.740337000000	-0.380246000000
H	5.471335000000	-0.913383000000	-0.492761000000
C	3.929424000000	0.525897000000	-0.047309000000
H	4.613793000000	1.354574000000	0.099369000000
C	2.556525000000	0.747101000000	0.094535000000
C	2.033708000000	2.099549000000	0.507392000000
H	1.502078000000	2.583092000000	-0.321300000000
H	2.880544000000	2.746180000000	0.747896000000
C	1.124326000000	2.009068000000	1.735726000000
H	1.569346000000	1.362587000000	2.495344000000
H	0.988431000000	3.002292000000	2.176699000000
C	-2.389169000000	-1.752908000000	1.743508000000
H	-2.908665000000	-2.076871000000	2.651867000000
H	-2.570526000000	-2.520490000000	0.984881000000
C	-3.002899000000	-0.431563000000	1.276850000000
H	-4.085988000000	-0.549634000000	1.141998000000
H	-2.863250000000	0.365621000000	2.016359000000
C	-2.954586000000	1.262823000000	-0.569218000000
H	-2.797478000000	2.071637000000	0.154136000000
H	-4.041085000000	1.158645000000	-0.685756000000
C	-2.316163000000	1.584053000000	-1.922269000000
H	-2.554448000000	0.784094000000	-2.630646000000
H	-2.771740000000	2.497325000000	-2.320103000000
O	-1.565626000000	-1.910852000000	-1.784332000000
H	-2.535131000000	-0.742400000000	-0.702605000000
O	-0.380407000000	-1.536629000000	-1.175439000000
H	-1.416135000000	-1.716715000000	-2.730998000000

Table S24 Coordinate file of the DFT optimized structure of complex **2c** in triplet state.

Cu	-0.334939000000	-0.039356000000	0.003667000000
N	1.687699000000	-0.273725000000	-0.099475000000
N	-0.212001000000	1.454184000000	1.380952000000
H	-0.669889000000	1.089914000000	2.215159000000
H	-0.801486000000	2.200914000000	1.015119000000
N	-0.928409000000	-1.608943000000	1.931539000000
H	-0.719062000000	-1.417316000000	2.910099000000
H	-0.470847000000	-2.495541000000	1.732760000000
N	-2.401106000000	0.006083000000	-0.009495000000
N	-0.843686000000	1.679800000000	-1.791728000000
H	-0.396819000000	1.420226000000	-2.668674000000
H	-0.578166000000	2.652370000000	-1.644619000000
C	2.139274000000	-1.502627000000	-0.404317000000
H	1.394010000000	-2.281253000000	-0.536193000000
C	3.493234000000	-1.779158000000	-0.555387000000
H	3.810443000000	-2.787871000000	-0.794340000000
C	4.405448000000	-0.740337000000	-0.380246000000
H	5.471335000000	-0.913383000000	-0.492761000000
C	3.929424000000	0.525897000000	-0.047309000000
H	4.613793000000	1.354574000000	0.099369000000
C	2.556525000000	0.747101000000	0.094535000000
C	2.033708000000	2.099549000000	0.507392000000
H	1.502078000000	2.583092000000	-0.321300000000
H	2.880544000000	2.746180000000	0.747896000000
C	1.124326000000	2.009068000000	1.735726000000
H	1.569346000000	1.362587000000	2.495344000000
H	0.988431000000	3.002292000000	2.176699000000
C	-2.389169000000	-1.752908000000	1.743508000000
H	-2.908665000000	-2.076871000000	2.651867000000
H	-2.570526000000	-2.520490000000	0.984881000000
C	-3.002899000000	-0.431563000000	1.276850000000
H	-4.085988000000	-0.549634000000	1.141998000000
H	-2.863250000000	0.365621000000	2.016359000000
C	-2.954586000000	1.262823000000	-0.569218000000
H	-2.797478000000	2.071637000000	0.154136000000
H	-4.041085000000	1.158645000000	-0.685756000000
C	-2.316163000000	1.584053000000	-1.922269000000
H	-2.554448000000	0.784094000000	-2.630646000000
H	-2.771740000000	2.497325000000	-2.320103000000
O	-1.565626000000	-1.910852000000	-1.784332000000
H	-2.535131000000	-0.742400000000	-0.702605000000
O	-0.380407000000	-1.536629000000	-1.175439000000
H	-1.416135000000	-1.716715000000	-2.730998000000

Table S25 Catalytic performance comparison between ternary complexes in this work and the reported pyridine based and aliphatic amine ligand containing copper complexes for water oxidation.

Catalyst	Electrolyte	pH	η (mV)	TOF (s^{-1})	FE (%)	Ref.
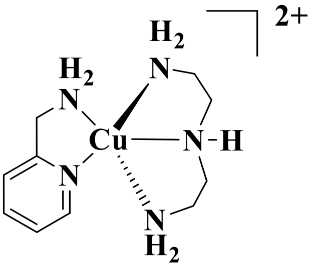	0.1 M PBS	7	423	157	94	This Work
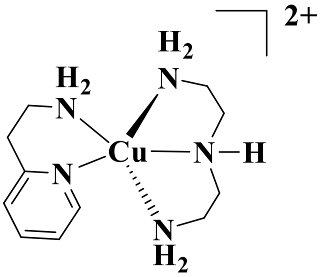	0.1 M PBS	7	483	704	97	This Work
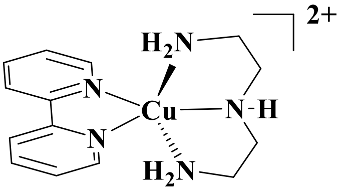	0.1 M PBS	7	453	483	98	[S6]
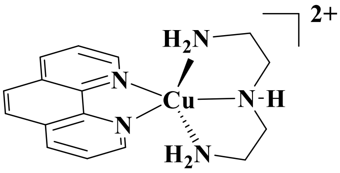	0.1 M PBS	7	493	445	96	[S6]
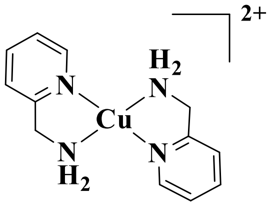	0.1 M PBS	7	503	5.81	90	[S7]
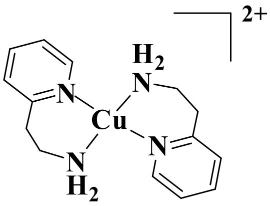	0.1 M PBS	7	413	70.39	97	[S7]
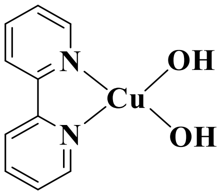	0.1 M NaOAc/NaOH	12.5	750	100	90	[S8]

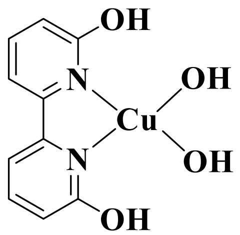	0.1 M NaOAc/NaOH	12.4	640	0.4	85	[S9]
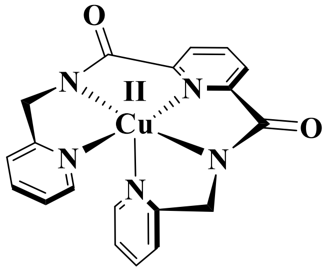	0.1 M PBS	8	N/A	38	84	[S10]
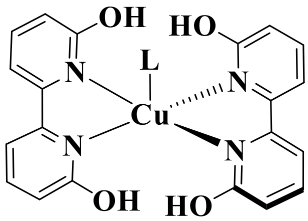	0.1 M NaOAc/NaOH	12.6	477	0.356	N/A	[S11]
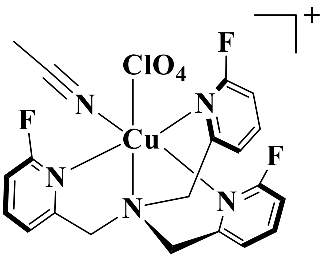	0.1 M borate solution	8.5	610	0.38	93	[S12]
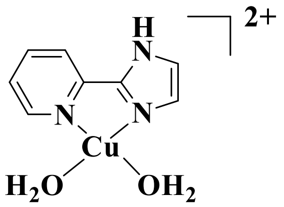	0.1 M NaOAc	12	300	35	N/A	[S13]
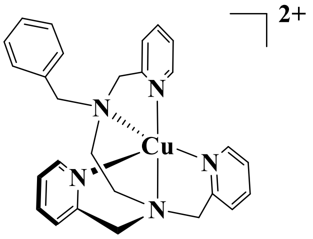	0.1 PBS	11.5	440	13.1	91	[S14]
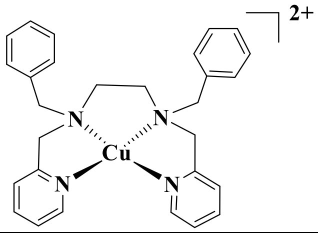	0.1 PBS	11.5	570	18.7	94	[S14]

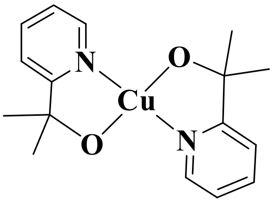	0.1 M KNO_3/NaOH	10.4	520	0.7	75	[S15]
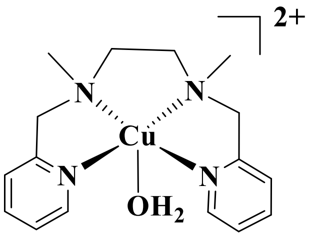	0.1 M PBS	7	874	4.1	91	[S16]
	0.1 M PBS	9	1007	13.5	69	[S17]
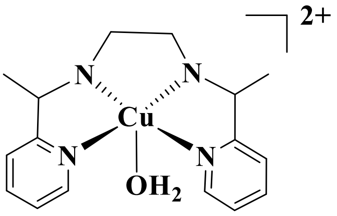	0.1 M PBS	9	700	50.4	94	[S17]
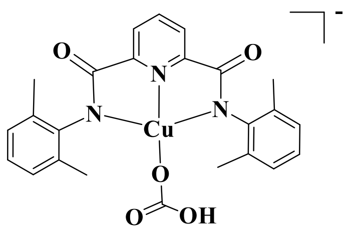	0.1 M carbonate solution	10	650	20.1	95	[S18]
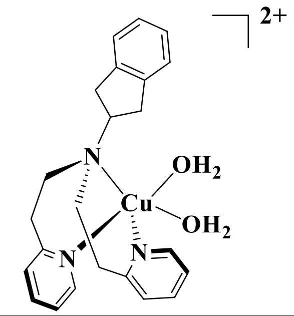	0.1 M $NaNO_3$	7	280	0.02	N/A	[S19]
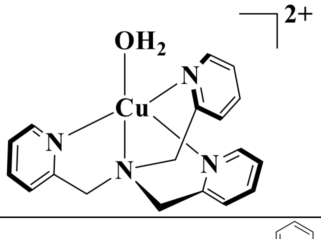	0.1 M PBS	7	970	0.1	>90	[S20]
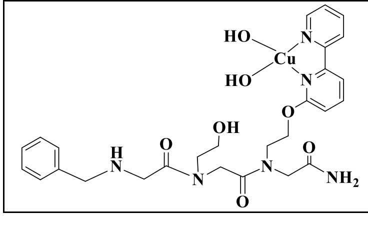	0.1 M PBS	11.5	800	5.8	91	[S21]

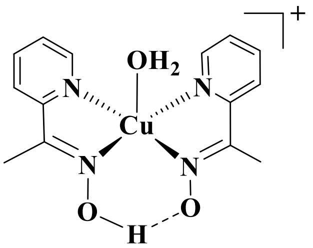	0.1 M PBS	7	650	100	94	[S22]
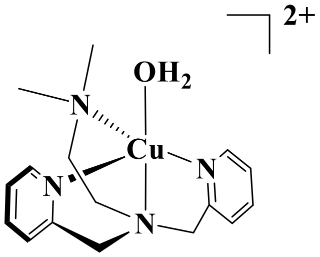	0.1M PBS	12	870	4.86	N/A	[S23]
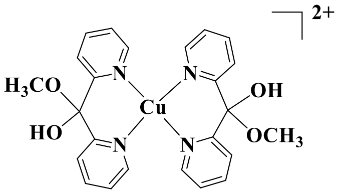	0.1 M NaOAc	7.81	602	0.28	84	[S24]
		11.84	909	9.20	86	
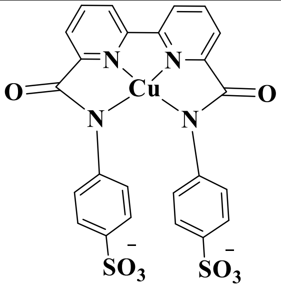	0.1 M PBS	11.6	830	10.5	76	[S25]
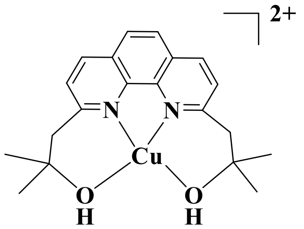	1 M bicarbonate	8.4	1100	131.6	N/A	[S26]
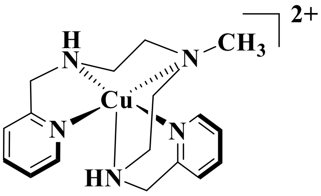	0.1 M PBS	7	640	0.04	88	[S27]
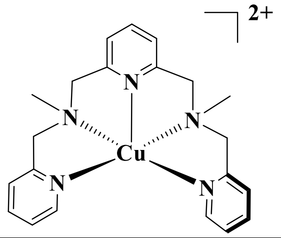	0.1 M PBS	11	831	0.81	90.7	[S28]

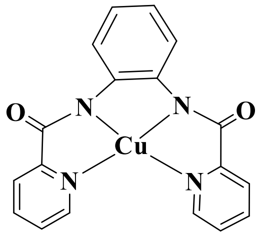	0.1 M PBS	13	697	88	79	[S29]
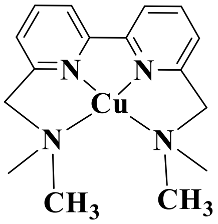	0.1M PBS	9	600	0.11	91	[S30]
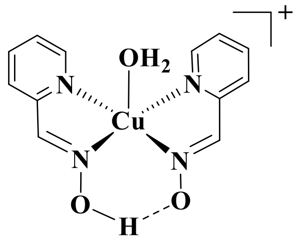	0.1 M PBS	7	693	32	90	[S31]
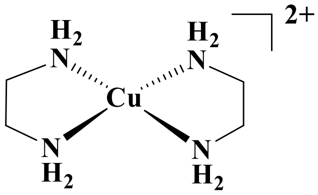	0.2 M PBS	8	540	0.4	75	[S32]
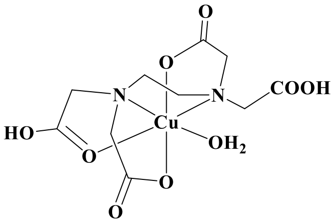	0.1 M PBS	7	684	8.03	95	[S33]
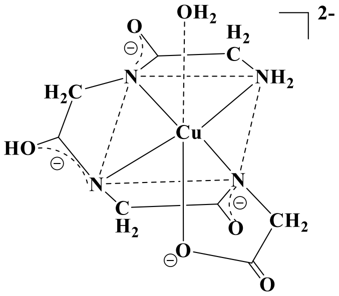	0.25 M PBS	11	520	33	99	[S34]
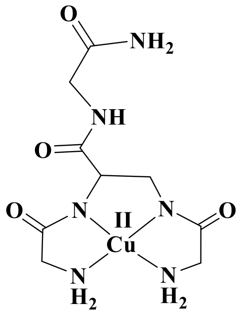	0.15 M PBS	11	620	53	95	[S35]

References:

S1 G. M. Sheldrick, A short history of *SHELX*, *Acta Cryst. A*, 2008, **64**, 112-122.

S2 G. M. Sheldrick, Crystal structure refinement with *SHELXL*, *Acta Cryst. C*, 2015, **71**, 3-8.

S3 L. J. Bourhis, O. V. Dolomanov, R. J. Gildea, J. A. K. Howard and H. Puschmann, The anatomy of a comprehensive constrained, restrained refinement program for the modern computing environment – *Olex2* dissected, *Acta Cryst. A*, 2015, **71**, 59-75.

S4 O. V. Dolomanov, L. J. Bourhis, R. J. Gildea, J. A. K. Howard and H. Puschmann, *OLEX2*: a complete structure solution, refinement and analysis program, *J. Appl. Cryst.*, 2009, **42**, 339-341.

S5 M.J. Frisch, G. W. Trucks, H. B. Schlegel, G. E. Scuseria, M. A. Robb, J. R. Cheeseman, G. Scalmani, V. Barone, B. Mennucci, G. A. Petersson, H. Nakatsuji, M. Caricato, X. Li, H. P. Hratchian, A. F. Izmaylov, J. Bloino, G. Zheng, J. L. Sonnenberg, M. Hada, M. Ehara, K. Toyota, R. Fukuda, J. Hasegawa, M. Ishida, T. Nakajima, Y. Honda, O. Kitao, H. Nakai, T. Vreven, J. A. Montgomery, J. E. Peralta, F. Ogliaro, M. Bearpark, J. J. Heyd, E. Brothers, K. N. Kudin, V. N. Staroverov, T. Keith, R. Kobayashi, J. Normand, K. Raghavachari, A. Rendell, J. C. Burant, S. S. Iyengar, J. Tomasi, M. Cossi, N. Rega, J. M. Millam, M. Klene, J. E. Knox, J. B. Cross, V. Bakken, C. Adamo, J. Jaramillo, R. Gomperts, R. E. Stratmann, O. Yazyev, A. J. Austin, R. Cammi, C. Pomelli, J. W. Ochterski, R. L. Martin, K. Morokuma, V. G. Zakrzewski, G. A. Voth, P. Salvador, J. J. Dannenberg, S. Dapprich, A. D. Daniels, O. Farkas, J. B. Foresman, J. V. Ortiz, J. Cioslowski, D. J. Fox, Gaussian 09, Revision A.1, Gaussian, Inc., Wallingford, CT, 2009.

S6 P. Das, H. Kuilya, S. Basak, B. Hazarika, J. Ray, D. Sarma, D. Choudhury and A. Kalita, Enhancing electrocatalytic water oxidation: ligand complementarity in ternary Cu (II) complexes, *Dalton Trans.*, 2025, **54**, 9418-9426.

S7 P. Das, S. Basak, A. Kashyap, K. Choudhury, D. Choudhury and A. Kalita, Effect of chelating ring size and bite angle on the electrocatalytic water oxidation activity of copper (II) complexes, *Polyhedron,* 2025, **280**, 117673.

S8 S. M. Barnett, K. I. Goldberg and J. M. Mayer, A soluble copper-bipyridine water-oxidation electrocatalyst, *Nature Chem*, 2012, **4**, 498-502.

S9 T. Zhang, C. Wang, S. Liu, J.-L. Wang and W. Lin, A Biomimetic Copper Water Oxidation Catalyst with Low Over-potential, *J. Am. Chem. Soc*., 2014, **136**, 273-281.

S10 M. K. Coggins, M.-T. Zhang, Z. Chen, N. Song and T. J. Meyer, Single-Site Copper (II) Water Oxidation Electrocatalysis: Rate Enhancements with HPO_4^{2-} as a Proton Acceptor at pH 8, *Angew. Chem. Int. Ed.*, 2014, **53**, 12226-12230.

S11 D. L. Gerlach, S. Bhagan, A. A. Cruce, D. B. Burks, I. Nieto, H. T. Truong, S. P. Kelley, C. J. Herbst-Gervasoni, K. L. Jernigan, M. K. Bowman, S. Pan, M. Zeller and E. T. Papish, Studies of the Pathways Open to Copper Water Oxidation Catalysts Containing Proximal Hydroxy Groups During Basic Electrocatalysis, *Inorg. Chem.*, 2014, **53**, 12689-12698.

S12 R.-J. Xiang, H.-Y. Wang, Z.-J. Xin, C.-B. Li, Y.-X. Lu, X.-W. Gao, H.-M. Sun and R. Cao, A Water-Soluble Copper-Polypyridine Complex as a Homogeneous Catalyst for both Photo-Induced and Electrocatalytic O_2 Evolution, *Chem. Eur. J.*, 2016, **22**, 1602-1607.

S13 L. A. Stott, K. E. Prosser, E. K. Berdichevsky, C. J. Walsby and J. J. Warren, Lowering water oxidation overpotentials using the ionisable imidazole of copper (2-(20-pyridyl)imidazole), *Chem. Commun.*, 2017, **53**, 651-654.

S14 J. Shen, M. Wang, P. Zhang, J. Jiang and L. Sun, Electrocatalytic water oxidation by copper (II) complexes containing a tetra- or pentadentate amine-pyridine ligand, *Chem. Commun.*, 2017, **53**, 4374-4377.

S15 K. J. Fisher, K. L. Materna, B. Q. Mercado, R. H. Crabtree and G. W. Brudvig, Electrocatalytic Water Oxidation by a Copper(II) Complex of an Oxidation-Resistant Ligand, *ACS Catal*., 2017, **7**, 3384-3387.

S16 J. Shen, X. Zhang, M. Cheng, J. Jiang and M. Wang, Electrochemical Water Oxidation Catalyzed by N_4-Coordinate Copper Complexes with Different Backbones: Insight into the Structure-Activity Relationship of Copper Catalysts, *ChemCatChem*, 2020, **12**, 1302-1306.

S17 J. Shen, M. Wang, J. Gao, H. Han, H. Liu and L. Sun, Improvement of Electrochemical Water Oxidation by Fine-Tuning the Structure of Tetradentate N_4 Ligands of Molecular Copper Catalysts, *ChemSusChem*, 2017, **10**, 4581-4588.

S18 F. Chen, N. Wang, H. Lei, D. Guo, H. Liu, Z. Zhang, W. Zhang, W. Lai and R. Cao, Electrocatalytic Water Oxidation by a Water-Soluble Copper (II) Complex with a Copper-Bound Carbonate Group Acting as a Potential Proton Shuttle, *Inorg. Chem.*, 2017, **56**, 13368-13375.

S19 M.-C. Kafentzi, R. Papadakis, F. Gennarini, A. Kochem, O. Iranzo, Y. Le Mest, N. Le Poul, T. Tron, B. Faure, A. J. Simaan and M. Réglier, Electrochemical Water Oxidation and Stereoselective Oxygen Atom Transfer Mediated by a Copper Complex, *Chem. Eur. J.*, 2018, **24**, 5213-5224.

S20 X.-J. Su, C. Zheng, Q.-Q. Hu, H.-Y. Du, R.-Z. Liao and M.-T. Zhang, Bimetallic cooperative effect on O–O bond formation: copper polypyridyl complexes as water oxidation catalyst, *Dalton Trans.*, 2018, **47**, 8670-8675.

S21 T. Ghosh, P. Ghosh and G. Maayan, A Copper-Peptoid as a Highly Stable, Efficient, and Reusable Homogeneous Water Oxidation Electrocatalyst, *ACS Catal.*, 2018, **8**, 10631-10640.

S22 H. Kuilya, N. Alam, D. Sarma, D. Choudhury and A. Kalita, Ligand assisted electrocatalytic water oxidation by a copper (II) complex in neutral phosphate buffer, *Chem. Commun.*, 2019, **55**, 5483-5486.

S23 X. Zhang, Y.-Y. Li, J. Jiang, R. Zhang, R.-Z. Liao and M. Wang, A Dinuclear Copper Complex Featuring a Flexible Linker as Water Oxidation Catalyst with an Activity Far Superior to Its Mononuclear Counterpart, *Inorg. Chem.*, 2020, **59**, 5424-5432.

S24 N. Shi, W. Xie, D. Zhang, Y.-H. Fan, L.-S. Cui and M. Wang, A mononuclear copper complex as bifunctional electrocatalyst for CO_2 reduction and water oxidation, *Journal of Electroanalytical Chemistry*, 2021, **886**, 115106.

S25 M. Gil-Sepulcre, P. Garrido-Barros, J. Oldengott, I. Funes-Ardoiz, R. Bofill, X. Sala, J. Benet-Buchholz and A. Llobet, Consecutive Ligand-Based Electron Transfer in New Molecular Copper-Based Water Oxidation Catalysts, *Angew. Chem. Int. Ed.*, 2021, **60**, 18639-18644.

S26 Q.-F. Chen, Z.-Y. Cheng, R.-Z. Liao and M.-T. Zhang, Bioinspired Trinuclear Copper Catalyst for Water Oxidation with a Turnover Frequency up to 20000 s^{-1}, *J. Am. Chem. Soc.*, 2021, **143**, 19761-19768.

S27 J. Lin, N. Wang, X. Chen, X. Yang, L. Hong, Z. Ruan, H. Ye, Y. Chen and X. Liang, Electrocatalytic water oxidation by copper (II) complexes with a pentadentate amine-pyridine ligand, *Sustainable Energy Fuels*, 2022, **6**, 1312-1318.

S28 K. Yu, T. Wang, Y. Sun, M. Kang, X. Wang, D. Zhu, S. Xue, J. Shen, Q. Zhang and J. Liu, Impact of the hybridization form of the coordinated nitrogen atom on the

electrocatalytic water oxidation performance of copper complexes with pentadentate amine-pyridine ligands, *Dalton Trans.*, 2024, **53**, 612-618.

S29 S. Khan, S. Sengupta, Md. A. Khan, Md. P. Sk, N. Ch. Jana and S. Naskar, Electrocatalytic Water Oxidation by Mononuclear Copper Complexes of Bis-amide Ligands with N4 Donor: Experimental and Theoretical Investigation, *Inorg. Chem.*, 2024, **63**, 1888-1897.

S30 J. Wang, Y. Ping, Y. Chen, S. Liu, J. Dong, Z. Ruan, X. Liang and J. Lin, Improvement of electrocatalytic water oxidation activity of novel copper complex by modulating the axial coordination of phosphate on metal center, *Dalton Trans.*, 2024, **53**, 5222-5229.

S31 H. Kuilya, P. Das, S. Basak, D. Sarma, P. Mazumdar, D. Choudhury and A. Kalita, Effect of ligand substituents on the reactivity pathways of copper (II) complexes towards electrocatalytic water oxidation, *Dalton Trans.*, 2024, **53**, 17547-17553.

S32 C. Lu, J. Du, X.-J. Su, M.-T. Zhang, X. Xu, T. J. Meyer and Z. Chen, Cu (II) Aliphatic Diamine Complexes for Both Heterogeneous and Homogeneous Water Oxidation Catalysis in Basic and Neutral Solutions, *ACS Catal.*, 2016, **6**, 77-83.

S33 K. Yu, Y. Sun, D. Zhu, Z. Xu, J. Wang, J. Shen, Q. Zhang and W. Zhao, A low-cost commercial Cu (II)–EDTA complex for electrocatalytic water oxidation in neutral aqueous solution, *Chem. Commun.*, 2022, **58**, 12835-12838.

S34 M.-T. Zhang, Z. Chen, P. Kang and T. J. Meyer, Electrocatalytic Water Oxidation with a Copper (II) Polypeptide Complex, *J. Am. Chem. Soc.*, 2013, **135**, 2048-2051.

S35 J. S. Pap, Ł. Szyrwiel, D. Srankó, Z. Kerner, B. Setner, Z. Szewczuk and W. Malinka, Electrocatalytic water oxidation by Cu^{II} complexes with branched peptides, *Chem. Commun.*, 2015, **51**, 6322-6324.
